# On‐Demand Ligate and Release Strategy Based on Photoclick Reaction in Tandem with Pd‐Mediated Deallylation

**DOI:** 10.1002/anie.202425479

**Published:** 2025-05-12

**Authors:** Boon Shing Loh, Hanqing Pang, Soh Wah Yong, Cheng Weng, Wee Han Ang

**Affiliations:** ^1^ Department of Chemistry National University of Singapore 3 Science Drive 3 Singapore 117543; ^2^ NUS Graduate School of Integrative Sciences and Engineering National University of Singapore 28 Medical Drive Singapore 117456

**Keywords:** Bioorthogonal chemistry, Deallylation, Labeling, Ligate‐and‐release, Photoclick reaction

## Abstract

The development of chemoselective tools that can conjugate, modify, and decouple chemical groups from biomacromolecules has enabled the study of biological processes at increasing levels of fidelity. Until recently, these tools can either couple chemical entities to biomacromolecules or decouple them, but not both. A method that can perform these functions in distinct steps on demand would be highly useful. To that end, we devised a new‐to‐nature strategy by bringing together and modifying two biocompatible transformations. In this new strategy, ligation is accomplished via the photoclick reaction between an allenyl motif with 9,10‐phenanthrenequinone which installs an allyl group at the site of conjugation. This allyl group can then be selectively utilized as a handle for phenolic release via Pd‐mediated deallylation. As a proof of concept, we demonstrated its utility in the selective labeling and delabeling of model protein scaffolds and cellular matrix. This multifunctional method paves the way for a controllable “ligate and release” strategy that enables on‐demand visualization of biological entities but with an in built release mechanism to restore their original state.

## Graphical abstract

A consecutive, controllable, and revertible new‐to‐nature ligation and cleavage strategy has been rationally devised to selectively visualize and manipulate biomolecules of interest. The photoclick cycloaddition between allenyl phenyl ether motifs and 9,10‐phenanthrenequinone analogues installed a terminal allyl handle at the exocyclic site, thereby enabling on‐demand Pd‐mediated deallylation to release phenolic payloads (copyright permission obtained from BioRender).

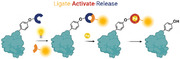

Since its advent two and a half decades ago, bioorthogonal chemistry has fundamentally transformed our approach to intervene and dissect biosystems.^[^ ^1^, ^2^, ^3^, ^4^, ^5^, ^6^ ^]^ The development of biocompatible moieties that can effect singular but highly selective transformation has availed the chemical tools needed to manipulate diverse biological entities at the molecular level. In the context of biomacromolecular transformations, bioorthogonal chemistry can be broadly categorised as ligation or cleavage reactions. While there are numerous examples of synthetic approaches designed to efficiently carry out either of the aforementioned transformations, few attempts have been made to develop methods that can do both. Such an approach would be highly useful to interface two bioorthogonal chemistry techniques for selective labeling and remodeling of biomolecules and subsequent on‐demand regeneration of their original state. Kim and co‐workers demonstrated a revertible conjugation approach using retro‐Cope elimination with cyclooctynes and *N,N*‐dialkylhydroxylamines to generate an enamine *N*‐oxide intermediate that was readily cleaved by diboron reagents to release the caged amine motif tracelessly.^[^ ^7^ ^]^ The strategy adopted organic small molecules as triggers for both ligation and cleavage.^[^ ^8^, ^9^ ^]^ To the best of our knowledge, this is the only example of a biocompatible “ligate and release” strategy. We envisioned that expanding the toolbox of these multifunctional reaction systems could unlock new possibilities for varied on‐demand spatiotemporal manipulations within biological milieu via devising and steering “ligate and release” transformation modalities.

In the design of caging chemistry for bond cleavage reactions, functional groups are purposefully encased within protecting groups (PGs) for downstream regeneration by specific triggers. One important functional group commonly found in medicine and biology is the phenolic moiety (Figure S1). Phenolic groups are crucial pharmacophores of anticancer drugs and antibiotics such as irinotecan, duocarmycin, etoposide, triclosan and are often caged by PGs as prodrugs.^[^ ^10^, ^11^, ^12^, ^13^, ^14^ ^]^ Phenol‐caged tyrosine residues are also used to probe phosphorylation events for cell signaling and protein–protein interactions.^[^ ^15^, ^16^, ^17^, ^18^ ^]^ Despite their prevalence, there are few techniques for the tagging and visualization, followed by regeneration of the phenolic motif within living systems in situ. Pre‐installing imaging and diagnostic tags directly on the substrate of interest, particularly sterically encumbered motifs, may hamper the intrinsic properties of parent bioactive species.^[^ ^19^ ^]^ Herein, we report a “ligate and release” strategy that would enable directed installation as well as removal of functional groups in distinct steps within biological settings, using a rationally designed photoclickable latch with an in built release mechanism.

Electron‐rich aliphatic vinyl ether was first reported to efficiently photoligate with 9,10‐phenanthrenequinone (**PQ**) under mild irradiation conditions via radical cycloaddition (Scheme 1a) by Zhang and co workers.^[^ ^20^, ^21^, ^22^ ^]^ Feringa and co‐workers extended this work to demonstrate that the photoligation efficacy could be influenced by changing the nature of adjacent functional groups on vinyl ether.^[^ ^23^, ^24^ ^]^ Separately, Chen and co‐workers showed that tyrosine residues in proteins of interest could be caged using allenyl phenyl ether (**APE**) and activated via bioorthogonal cleavage reactions.^[^ ^25^ ^]^ We reasoned that **APE** would also be amenable to photoligation given its structural similarity to electron‐rich vinyl ethers. We further rationalized that photoligation on **APE** would afford a terminal double bond as **PQ** should preferentially cyclize at the 1‐ene position adjacent to the electron‐donating oxygen moiety. The resulting product would yield a terminal allyl handle that could be exploited for subsequent phenolic release via deallylation reactions (Scheme 1b).^[^ ^26^, ^27^, ^28^, ^29^, ^30^, ^31^, ^32^ ^]^

**Scheme 1:**
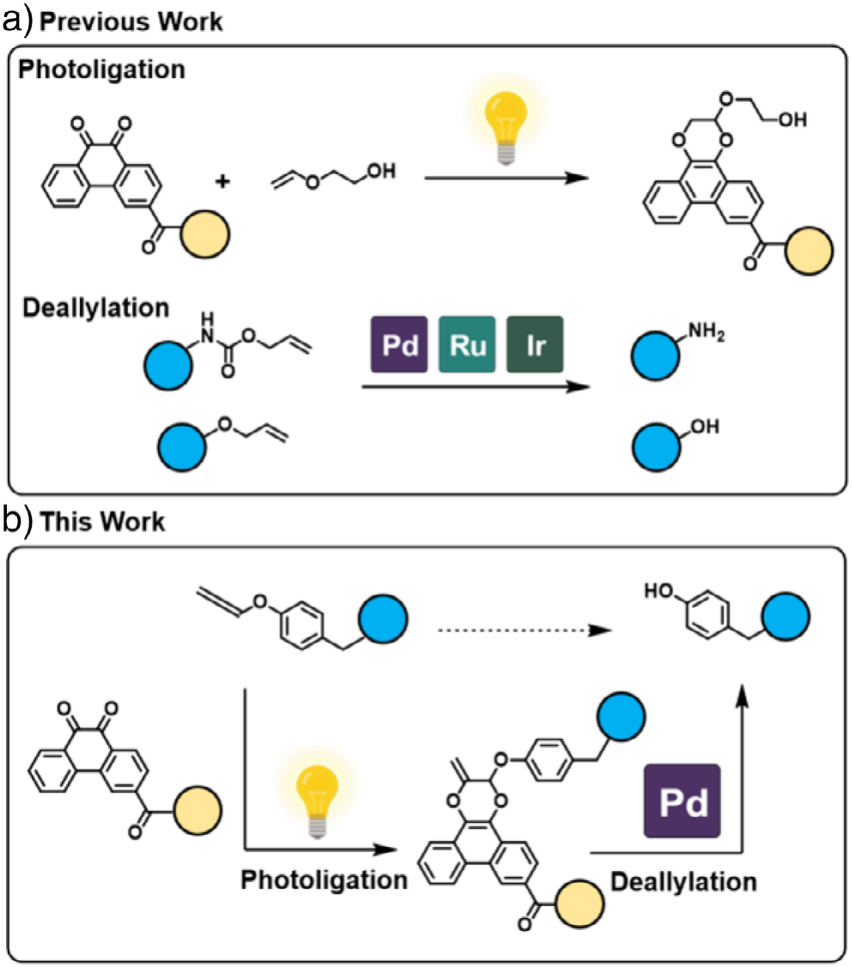
a) Previous work on bioorthogonal photocycloaddition between **PQ** and alkyl vinyl ethers, and biocompatible deallylation in cellular context. b) Photoclick between **PQ** and **APE** yielding stable **allyl‐PDO** for subsequent phenolic release when treated with Pd species (copyright permission obtained from BioRender).

As a proof of concept, we investigated whether the phenyl vinyl ether was sufficiently electron‐rich to undergo photoligation with **PQ** because of its commercial availability and similar electronic and reactive properties to **APE**. Gratifyingly, the desired 2‐phenoxyphenanthrodioxine (**PDO**) cycloadduct, characterized by HPLC and ^1^H‐NMR, was formed, indicating that phenyl vinyl ether could be a suitable PG (Figure S2). Extending vinyl to allenyl should not affect its photoligation to **PQ** as the π orbitals of the allene are perpendicular to each other and thus would not delocalize across one another. Indeed, photoirradiation of **PQ** with **APE** in 1:1 v/v acetonitrile‐d_3_:D_2_O by 18 W blue LED strips (425–525 nm, 7 mW cm^−2^) at ambient conditions yielded exclusively 2‐methylene‐3‐phenoxyphen‐anthrodioxine (**allyl‐PDO**) adduct, which was characterized using ^1^H NMR, ESI‐MS and single‐crystal X‐ray diffraction analyses (Figures 1a and S3). Furthermore, the photoligation proceeded efficaciously with either excess **PQ** or **APE**, in contrast to previous reports that excess aliphatic olefin was required to drive the reaction to completion (Figures 1b and S4).^[^ ^20^ ^]^ Complete consumption of **APE** was observed within 15 min as evidenced by the disappearance of its characteristic peak at 5.91 ppm (Figures 1c and S5). No 2‐ene cycloadduct was observed on ^1^H‐NMR. X‐ray crystallography also corroborated our hypothesis that cycloaddition with **PQ** occurred regioselectively at the internal olefin, affording the **allyl‐PDO** product in a half‐chair conformation with C7‐atom lying out‐of‐plane (Figure 1d and Table S1). The olefin C8‐C9 bond length of 1.3148(19) Å and acetal C7‐O1/C7‐O2 of 1.4241(15)/1.4171(15) Å were in keeping with a reported dihydrodibenzodioxinone natural product derived from *hypericum* plant, which shared a similar core structure, with olefin and acetal bond lengths of 1.337 Å and 1.408/1.413 Å, respectively.^[^ ^33^ ^]^ The rate constant of photoligation between **PQ** and **APE** initiated by blue LED strips was determined as 0.50 M^−1^ s^−1^ using ^1^H NMR (Figure S6), comparable to that of photoligation using **PQ** and electron‐rich aliphatic vinyl ether.^[^ ^20^ ^]^

**Figure 1:**
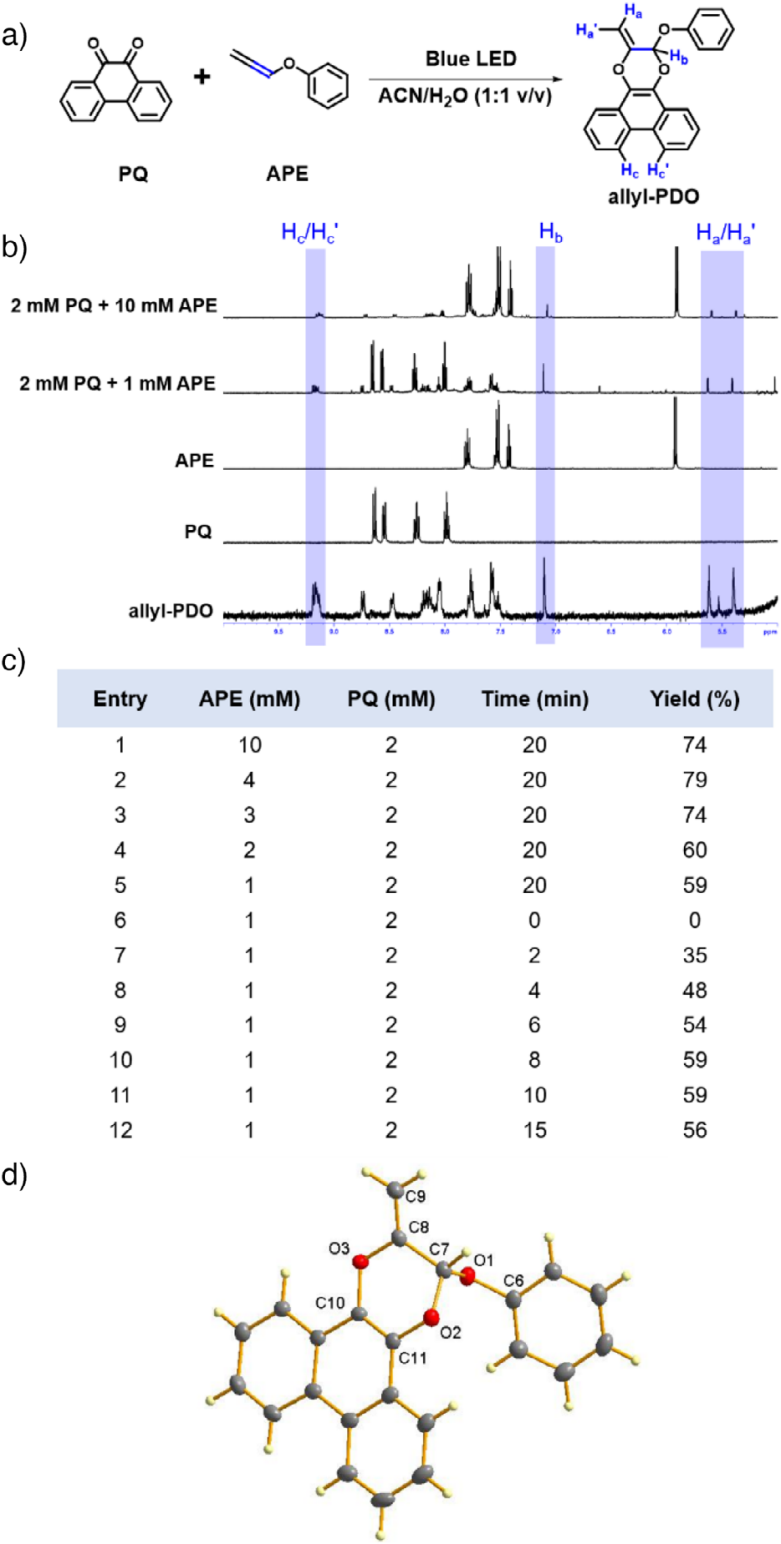
a) Reaction conditions for photoligation between **PQ** and **APE**. b) ^1^H NMR spectra showing the formation of **allyl‐PDO** (characteristic peaks highlighted in blue) in 2 mM PQ with 1 or 10 mM APE c) Photoligation optimization for **allyl‐PDO** product yield. d) Solid‐state structure of **allyl‐PDO** (CCDC deposition number 2337359).

Metal‐mediated deallylation in biological milieu was first demonstrated by Meggers in 2006 using piano‐stool Ru complexes.^[^ ^34^ ^]^ Since then, there has been a surge in interest to harness other transition metals such as Ir and Pd for this class of reaction.^[^ ^35^, ^36^, ^37^, ^38^, ^39^, ^40^ ^]^ The reaction typically proceeds via the Tsuji–Trost mechanism with Pd(0) as the catalytic priming species. Coordination of Pd(0) to the allyl group and subsequent formation of the π‐allyl Pd intermediate expels an anionic RO^−^ leaving group hence achieving bond cleavage across a C─O bond.^[^ ^27^, ^35^, ^41^ ^]^ Ru and Ir catalysts have been postulated to mediate deallylation via a similar mechanism. Alternatively, the deallylation can also be mediated by Pd(II) via Wacker–Tsuji oxidation pathway.^[^ ^27^ ^]^ Upon complexation with allyl, Pd(II) could facilitate allylic hydration to generate hydroxyl moieties in situ, thereby allowing hydroxyl oxidation by Pd(II) via hydride transfer to yield a carbonyl intermediate as well as Pd(0). The subsequent β‐elimination enabled by carbonyl would cleave the C─O bond to release the phenolic payload. Pd(0)‐mediated catalysis can proceed with either Tsuji–Trost pathway on allyl caged substrate or oxidation by media components to regenerate Pd(II) for second substrate turnover. To date, only simple terminal allyl phenyl ether groups were studied while sterically encumbered allyl scaffolds remain unexplored for the purpose of metal‐mediated decaging.

We therefore subjected **allyl‐PDO** to a panel of Pd catalysts (100 µM **allyl‐PDO**, 1:1 v/v THF/PBS) at 37 °C without additives and determined the corresponding yield of released phenol using HPLC and GC‐MS (Figures 2a,b and S7). Remarkably, Pd_2_(dba)_3_ and Na_2_PdCl_4_ demonstrated moderate yields at 48 ± 0.3% and 58 ± 4.6%, respectively, at high reaction dilutions (Figures 2b and S8). Bernardes and coworkers demonstrated that additives such as the reducing agents sodium ascorbate (NaAsc) and TPPTS, or nucleophiles such as morpholine, could increase deallylation yields of Na_2_PdCl_4_, through either in situ generation of Pd(0) species or facilitating the release of phenol.^[^ ^42^ ^]^ However, there was no significant change in yields when 1.0 equiv. of corresponding additives were co‐incubated with Na_2_PdCl_4_ (Figure S9), implying that Pd(0) and Pd(II) species presumably demonstrated similar efficiencies in the formulated deallylation regime in line with previous screening results. The rate constant of **allyl‐PDO** deallylation mediated by Na_2_PdCl_4_ was determined as 1.20 × 10^−4^ s^−1^ using HPLC (Figure S10). **Allyl‐PDO** was stable across pH 4 to 9, suggesting that phenol release was due to Pd‐mediated deallylation instead of acetal hydrolysis (Figure S11). Notably, **PDO** remained intact under the same reaction conditions, vindicating our assumption that the allyl handle in the cycloadduct was crucial for the deallylation reaction (Figure 2c).

**Figure 2:**
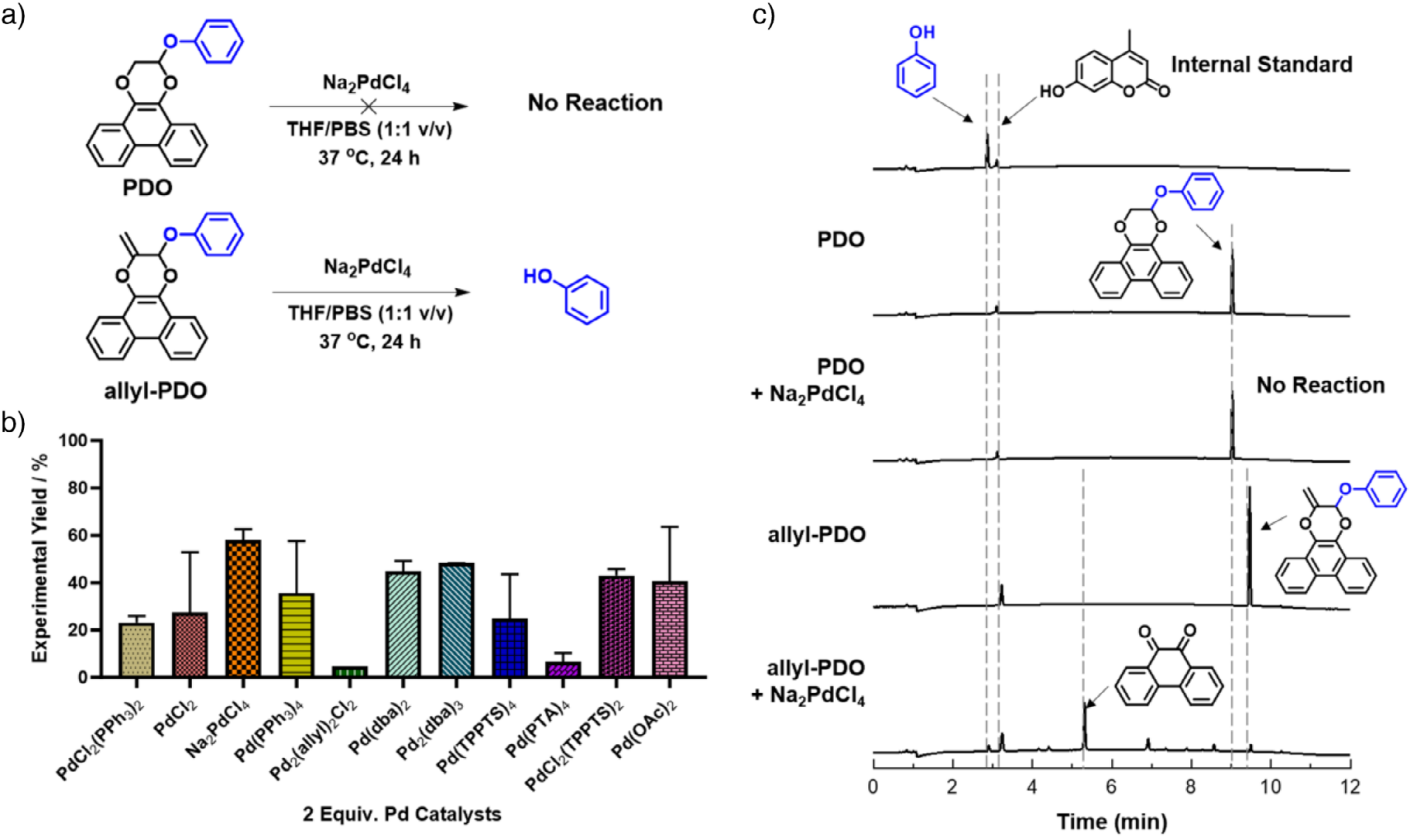
a) Deallylation of **allyl‐PDO** and **PDO** mediated by Na_2_PdCl_4_ incubated in 1:1 v/v THF/PBS at 37 °C for 24 h. b) Screening of deallylation efficacies using library of Pd‐based catalysts with **allyl‐PDO** as substrate. Experimental yields of phenol were determined by HPLC. The experiment was conducted with 3 replicates and error bars indicate Mean ± SD. c) HPLC chromatograms for Pd‐mediated deallylation of **PDO** and **allyl‐PDO** with phenol as positive control and 4‐methylumbelliferone as internal standard.

To demonstrate the biocompatibility and applicability of “ligate and release” strategy on biomacromolecular functionalization, lysozyme was selected as a model protein for modification using **APE** as a reaction handle for subsequent **PQ** coupling and decoupling (Figure 3a). Photoligation was first assessed using in‐gel fluorescence assays through the attachment of either **PQ** or rhodamine‐conjugated **PQ** (**Rho‐PQ**). In‐gel fluorescence could be observed with *λ*_ex _= 302 nm in **APE**‐lysozyme treated with **PQ** and light (Figures 3b, S12 and S13).^[^ ^20^ ^]^ Next, the photoligated **allyl‐PDO**‐lysozyme was exposed to different decaging conditions. Gratifyingly, Pd(II) species efficiently mediated the regeneration of phenolic scaffolds on lysozyme via deallylation to yield **phenol**‐lysozyme evidenced by the lack of fluorescence after excitation at 302 nm (Figures 3c and S14). Likewise, in‐gel fluorescence with *λ*_ex_ = 532 nm and Pd‐mediated deallylation was also observed using **Rho‐PQ** (Figure S15). We excluded fluorescence quenching due to Na_2_PdCl_4_ alone since fluorescence from the adduct **PDO** remained unperturbed when co‐incubated with Na_2_PdCl_4_ across different concentrations, indicating that the fluorescence disappearance was tied to **allyl‐PDO** decaging (Figure S16).

**Figure 3:**
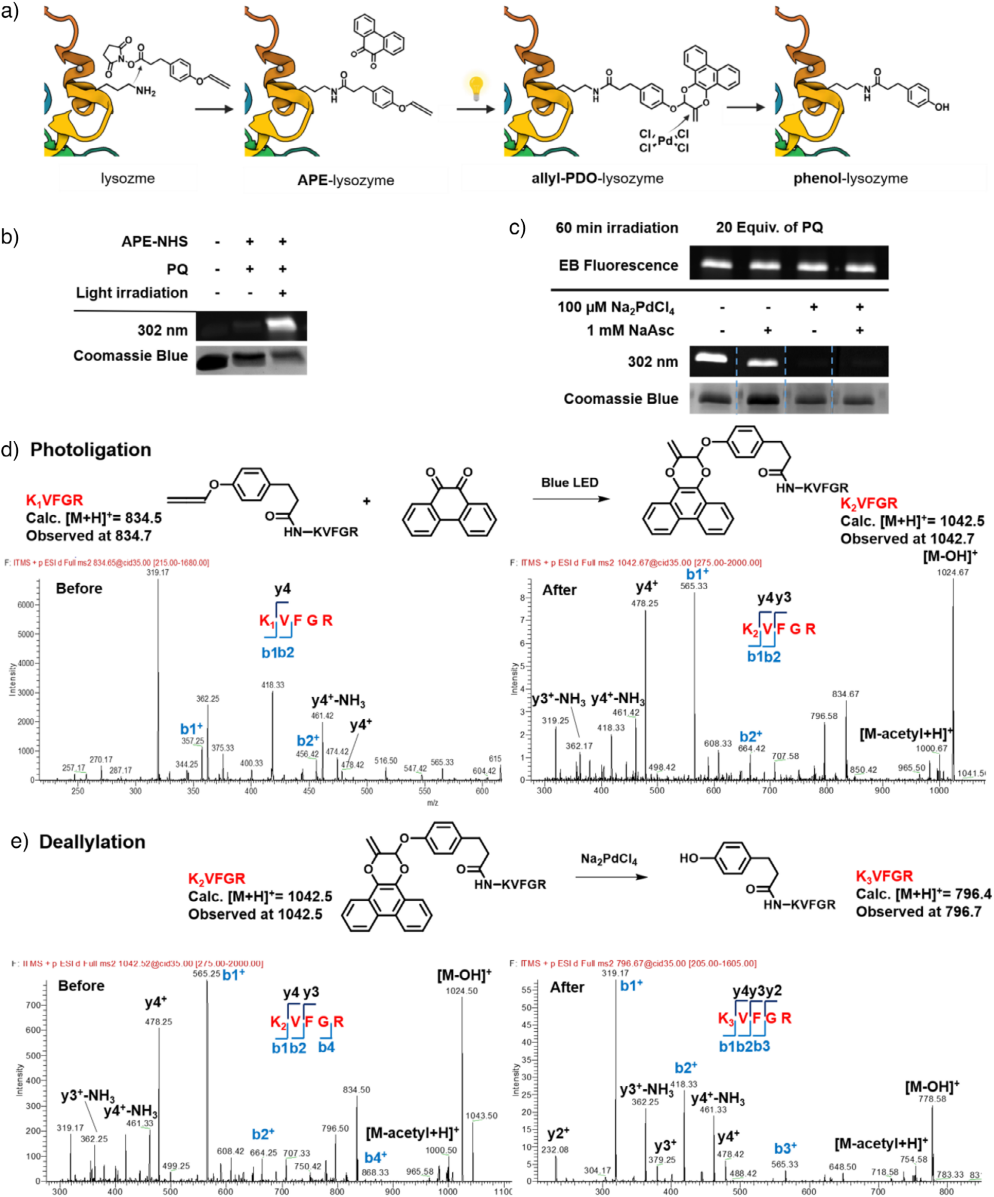
a) Schematic illustration of sequential photoligation between **APE** motif and **PQ** and Pd‐mediated deallylation on model protein lysozyme (copyright permission obtained from BioRender). b) In‐gel fluorescence of lysozyme treated with or without **APE** motif and **PQ** under dark or visible light irradiation. Uncropped gel images can be found in Figure S13. c) In‐gel fluorescence of **allyl**‐**PDO**‐lysozyme treated with/without Na_2_PdCl_4_ and/or NaAsc. The gel in (B) was cut into four pieces as indicated by the blue dotted line, each treated under different deallylation conditions, and then placed side by side for imaging. Uncropped gel images can be found in Figure S14. d) LC‐MS/MS analysis of photoligation reaction on *N*‐acetylated peptide fragment **KVFGR**. e) LC‐MS/MS analysis of decaging reaction on *N*‐acetylated peptide fragment **KVFGR**.

Encouraged by in‐gel fluorescence assays, we proceeded to validate the “ligate and release” reaction directly on model *N*‐acetylated peptide **KVFGR**, a fragment derived from lysozyme. **KVFGR** was first modified using 2,5‐dioxopyrrolidin‐1‐yl 3‐(4‐(propa‐1,2‐dien‐1‐yloxy)phenyl)propanoate (**APE‐NHS**) to form **APE‐KVFGR** (K_1_VFGR). After reacting with **PQ** under blue light irradiation for 1 h, the formation of **allyl‐PDO‐KVFGR** (K_2_VFGR) was confirmed on LC‐MS/MS (Figures 3d and S17). Likewise, **allyl‐PDO‐KVFGR** was prepared from **KVFGR** and characterized. Upon Na_2_PdCl_4_ treatment for 24 h at 37 °C, **allyl‐PDO‐KVFGR** underwent Pd‐mediated deallylation to regenerate **phenol‐KVFGR** (K_3_VFGR), as observed using LC‐MS/MS (Figures 3e and S17). These results validated the efficacy of the stepwise “ligate and release” for on‐demand manipulation of proteins of interest containing functionalized amino acids, e.g. allenyl‐caged tyrosine, including those generated through genetic encoding.^[^ ^25^ ^]^

To demonstrate the efficacy of complex cellular manipulation using these tools, fluorophore labeling, and decoupling on intracellular proteins were studied using confocal laser scanning microscopy (CLSM). **APE** motif was first conjugated to free lysine residues in HeLa cells through amide coupling with biocompatible **APE‐NHS** (Figure S18), followed by photo labeling with **Rho‐PQ** (Figure 4a). As expected, **Rho‐PQ** labeling only occurred in the presence of **APE** under visible light irradiation (380–800 nm, 15 mW cm^−2^), as seen from an intense red fluorescence from treated cells (Figure 4b,c), confirming the high fidelity and selectivity of the photoligation reaction within a cellular environment. Upon Na_2_PdCl_4_ treatment at nontoxic concentrations (Figure S18), fluorescence intensity declined significantly which we attributed to **Rho‐PQ** liberation from modified proteins after decoupling (Figure 4d). Likewise, the concurrent addition of Na_2_PdCl_4_ (100 µM) with NaAsc (1.0 mM) for in situ Pd(0) generation reduced fluorescence by 1.8‐fold compared to sole Na_2_PdCl_4_ treatment (100 µM) of 1.3‐fold (Figure 4e). This indicated that exogenous reductants could improve reactivities since Pd(II) salts could be more readily deactivated by biological nucleophiles.^[^ ^25^, ^43^ ^]^ The same trends were observed using TAMRA‐conjugated **PQ** (Figure S19). To rule out fluorescence quenching from the Pd reagents, rhodamine and TAMRA were incubated with up to 20 equiv. of Na_2_PdCl_4_ in the presence or absence of NaAsc for 24 h at ambient conditions. As expected, the fluorescence remained unchanged (Figure S20), which showed that Pd‐mediated intracellular deallylation was responsible for the diminished emission. Taken together, the sequential photoligation and Pd‐mediated cleavage reactions were demonstrated to proceed in the complex cellular environment with high fidelity and efficacy, providing a new platform for on‐demand intracellular labeling and visualization as well as restoration of cellular matrix.

**Figure 4:**
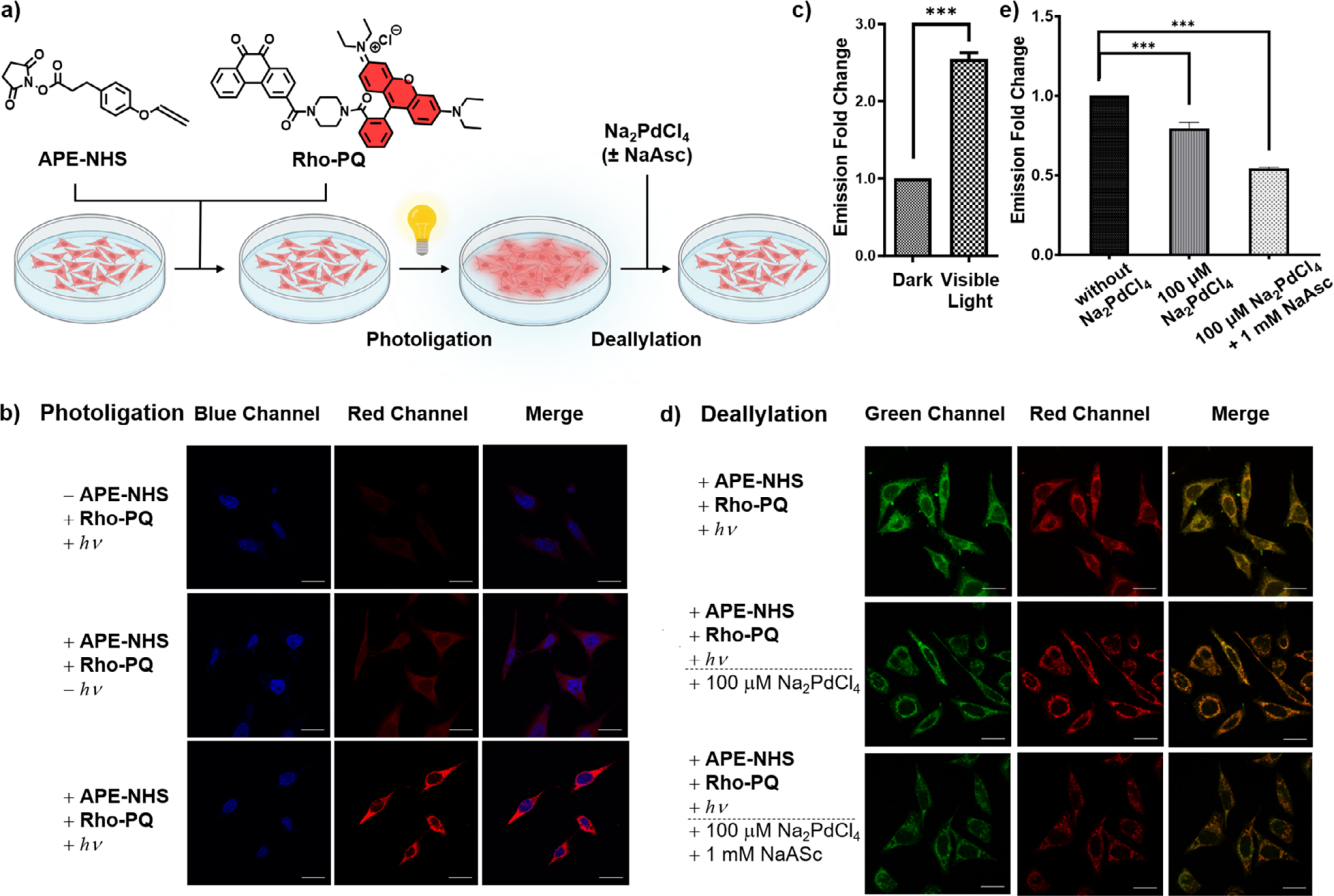
a) Schematic illustration of sequential photoligation between **APE** motif and **Rho‐PQ** and Pd‐mediated deallylation within HeLa cells (copyright permission obtained from BioRender). b) CLSM images of intracellular photoligation using **APE** motif and **Rho‐PQ** upon 1 h visible light irradiation. Control staining using Hoechst 33342 was viewed with the blue channel (*λ*_ex_ 405 nm/*λ*_em_ 461 nm). **Rho‐PQ** was viewed with the red channel (*λ*_ex_ 559 nm/*λ*_em_ 577 nm). c) Mean fold change in fluorescence intensities before and after photoligation. d) CLSM images of intracellular deallylation in cells treated with 100 µM Na₂PdCl₄, with or without 1 mM NaAsc, respectively. Control staining using rhodamine 123 was viewed with the green channel (*λ*_ex_ 473 nm/*λ*_em_ 519 nm). **Rho‐PQ** was viewed with the red channel (*λ*_ex_ 559 nm/*λ*_em_ 577 nm). e) Mean fold change in fluorescence intensities upon Na₂PdCl₄ administration to HeLa cells. Scale bar: 20 µm. Quantification of fluorescence intensity was performed using ImageJ. Unpaired T test was performed using GraphPad Prism 10 software with *p* < 0.05 considered as significant (*p < 0.05, **p < 0.01, ***p < 0.001).

In summary, we developed a novel “ligate and release” strategy based on photoclick reaction working in tandem with Pd‐mediated deallylation. The highly facile photoligation reaction was designed to chemoselectively label target biomatrices as well as to install an allyl handle that could be utilized to controllably unmask a terminally caged phenolic motif. Upon deallylation via Pd complexes, restoration of biologically important phenolic functional groups was accomplished. The devised new‐to‐nature reaction scheme was capable of spatiotemporally manipulating proteins of interest and cellular matrix using light and metal catalysts, potentially for the downstream remodeling, tracking, and gain‐of‐function studies in living systems. This strategy paves the way for interfacing bioorthogonal ligation and cleavage reactions, through rational functional group design, culminating in a new platform for consecutive on‐demand manipulation of biochemical groups.

## Conflict of Interests

The authors declare no conflict of interest.

## Supporting information



Supporting Information

Supporting Information

## Data Availability

The data that support the findings of this study are available in the Supporting Information of this article.
