# Supplementary material for: On‐Demand Ligate and Release Strategy Based on Photoclick Reaction in Tandem with Pd‐Mediated Deallylation
Source: Angew Chem Int Ed Engl. 2025 May 12;64(28):e202425479. doi: 10.1002/anie.202425479 (PMC12232882; doi:10.1002/anie.202425479)
Supplement: Supplementary file 1 — Supporting Information [file ANIE-64-e202425479-s002.pdf]

Supporting Information

On-Demand Ligate and Release Strategy Based on Photoclick Reaction in Tandem with Pd-Mediated Deallylation

Boon Shing Loh,[1,+] Hanqing Pang,[1,+] Soh Wah Yong,[1] Cheng Weng,[1,*] and Wee Han Ang[1,2,*]

[1]Department of Chemistry, National University of Singapore, 3 Science Drive 3, Singapore 117543, Singapore

[2]Integrative Sciences and Engineering Programme, National University of Singapore, 28 Medical Drive, Singapore 117456, Republic of Singapore

* All correspondence should be addressed to chmchew@nus.edu.sg and ang.weehan@nus.edu.sg

Table of Contents

Experimental sections	S2-17
Tables	S18
Figures	S19-30
^{1}H NMR and $^{13}C\{^{1}H\}$ NMR spectra of synthesized compounds	S31-42
HPLC chromatograms of synthesized compounds	S43
High resolution mass spectrometry spectra	S44-48
References	S49

Experimental Sections

Materials and Instrumentation. All experiments were carried out without exclusion of moisture and air unless otherwise specified. All chemical precursors and solvents were obtained from commercial sources without further treatment. All chemical precursors and solvents were of analytical grade or high-performance liquid chromatography (HPLC) grade purchased from commercially available sources. Lysozyme was obtained as lyophilized powder from chicken egg white source under Sigma Aldrich, L6876. Peptide KVFGR with N terminal acetylation was purchased from GenScript. 10X Phosphate Buffered Saline (PBS) and sodium dodecyl sulfate (10 %) were obtained from vivantis. HeLa cells were grown in RPMI 1640 nutrient media, Cytiva (cat no. SH30027.10). Trypsin for trypsin digestion was obtained as lyophilized powder extracted from porcine pancreas source under Sigma Aldrich, T4799.

^{1}H and $^{13}C\{^{1}H\}$ NMR spectra were obtained using either Bruker Avance III HD 400 or Bruker AVNEO 500 spectrometer, and the chemical shifts (δ) are reported in parts per million (ppm) with reference to residual solvent peaks. Electrospray-ionization mass spectrometry (ESI-MS) spectra were obtained using Thermo Scientific LC Fleet LCMS. Electron impact mass spectrometry (EI-MS) spectra were obtained using Agilent 7200 Q-TOF GCMS. Atmospheric pressure chemical ionization mass spectrometry (APCI-MS) spectra were obtained using Thermo Scientific LCQ Fleet LCMS. The HPLC was Shimadzu Prominence System equipped with a DGU-20A3 degasser, two LC-20AD liquid chromatography pumps, a SPD-20A UV/vis detector, and an Agilent, ZORBAX Eclipse Plus C18 column (4.6 × 150 mm, 5 μM) with flow rate of 1.0 mL/min with 254 and 280 nm detection wavelength. The gradient elution conditions were 20-80% of solvent B (ACN) and solvent A (H_2O) over a 30 min elution period. Reactions were analyzed using PerkinElmer LC300 Ultra High-Performance Liquid Chromatography coupled with photodiode array detector. Column used was PerkinElmer Brownlee SPP C18, 100 mm × 2.1 mm × 1.9 μm. The flow rate of 0.3 mL/min with 280 nm detection wavelength and reference wavelength at 400 nm with bandwidth of 1 nm and sampling rate of 5 Hz. A linear gradient elution of 10-100% solvent B (ACN) and solvent A (H_2O) was performed over a 12 min elution period. UV−vis spectra were obtained with Shimadzu UV-1800 UV spectrophotometer with a TCC-240A temperature-controlled cell holder. LC-MS spectra were obtained using Thermo Scientific Vanquish HPLC coupled to LTQ XL linear quadrupole ion trap MS.

ZORBAX Eclipse Plus C18 column (4.6 × 150 mm, 5 μm) was used with flow rate of 1.0 mL/min. The gradient elution conditions were 5-50% ACN in H_2O (0.1% FA) over 20 min, equilibration at 95% ACN in H_2O (0.1% FA) for 5 min to allow full elution. GCMS was conducted with a Single Quad GC-MS equipped with an Agilent 7890A gas chromatograph and an Agilent 5975C mass spectrometer. Blue LED strips (18 W, 425–525 nm, 7 mW cm^{-2}) were used for the chemical experiments, and visible light irradiation (380–800 nm, 15 mW cm^{-2}) was employed for the biological tests.

Synthesis of allenyl phenyl ether (APE)

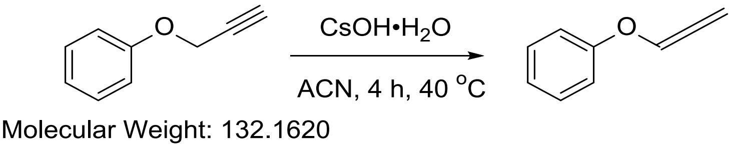

Slight modification was made to the following protocol.[1] Excess $CsOH \cdot H_2O$ and 20 mL ACN was charged to 100 mL round bottom flask. 2 mmol phenyl propargyl ether was added to the reaction mixture. The reaction was heated at 40 °C for 4 h with condenser in regard to the high volatility nature of phenyl propargyl ether. The reaction solvent was removed *in vacuo*. The reaction mixture was dissolved in H_2O and extracted with hexane thrice. The combined organic layers were concentrated under reduced pressure to 2 mL. The desired product was purified by flash column chromatography on silica gel by dry loading using eluent hexane only.

TLC (Hexane), R*f*: 0.43

Yield: 228.1 mg (86.3%). ^1H NMR (500 MHz, $CDCl_3$): δ 5.45 (2H, d, J=5.88 Hz), 6.85 (1H, t, J=5.92 Hz), 7.07 (3H, t, J=4.34 Hz), 7.32 (2H, q, J=5.34 Hz) ppm. $^{13}C\{^1H\}$-NMR (125 MHz, $CDCl_3$): δ 89.61, 116.87, 117.94, 122.85, 129.60, 157.26, 202.91 ppm. HRMS (EI): m/z calculated for C_9H_8O, $[M]^+$ = 132.057, found: 132.0565.

Photoligation between 9,10-phenanthrenequinone (PQ) and phenyl vinyl ether

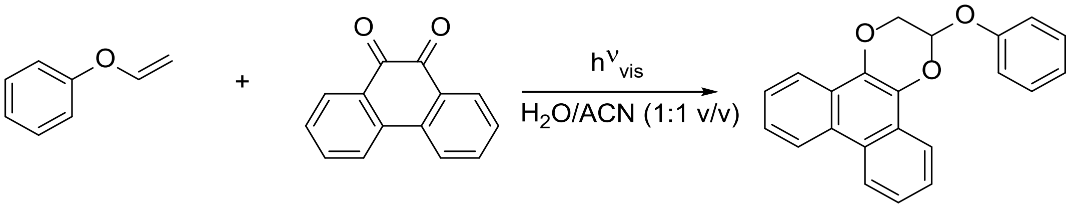

PQ (0.55 mmol) and phenyl vinyl ether (0.66 mmol, 1.20 equiv) were dissolved in 500 mL 1:1 v/v acetonitrile/water. The mixture was stirred and irradiated with blue light LED strips for 180 min. The solvent mixture was removed *in vacuo*. The desired product was purified by flash column chromatography on silica gel by dry loading using eluent hexane to 1:50 v/v ethyl acetate/hexane.

TLC (1:40 v/v ethyl acetate/hexane), R*f*: 0.34

Yield: 13.4 mg (7.4%). ^{1}H NMR (500 MHz, $CDCl_3$): δ 4.50 (1H, dd, J=1.70, 11.10 Hz), 4.70 (1H, dd, J=2.68, 11.13 Hz), 6.15 (1H, q, J=1.43 Hz), 7.08 (1H, m, J=2.40 Hz), 7.23 (2H, m, J=1.64 Hz), 7.34 (2H, q, J=5.35 Hz), 7.55 (1H, m, J=2.40 Hz), 7.59 (3H, m, J=2.49 Hz), 7.64 (1H, m, J=2.72 Hz), 8.14 (1H, m, J=2.29 Hz), 8.26 (1H, m, J=2.28 Hz), 8.61 (2H, m, J=1.71 Hz) ppm. $^{13}C\{^{1}H\}$-NMR (125 MHz, $CDCl_3$): δ 29.73, 66.29, 92.78, 117.09, 120.55, 121.03, 122.47, 122.53, 122.94, 125.13, 125.34, 125.91, 126.07, 126.78, 126.82, 126.98, 129.64, 130.47, 133.28, 156.58 ppm. HRMS (EI): m/z calculated for $C_{22}H_{16}O_3$, $[M]^+$ = 328.1094, found: 328.1092. Purity (HPLC): > 99%.

Photoligation between PQ and APE

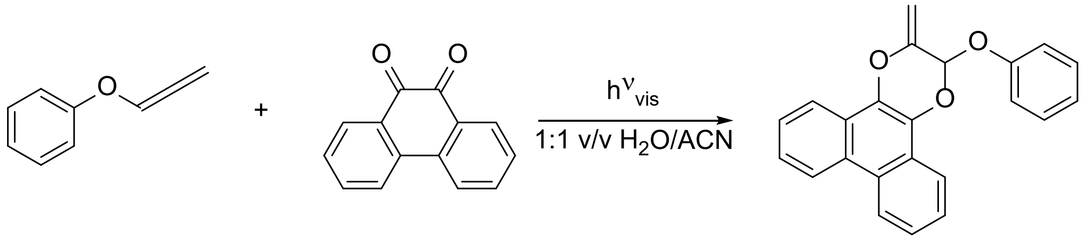

PQ (1.564 mmol, 1.20 equiv) and **APE** (1.304 mmol) were dissolved in 500 mL 1:1 v/v acetonitrile/water. The mixture was stirred and irradiated with blue light LED strips for 120 min. The solvent mixture was removed *in vacuo*. The desired product was purified by flash column chromatography on silica gel by dry loading using eluent hexane.

TLC (1:40 v/v ethyl acetate/hexane), R*f*: 0.75

Yield: 120.3mg (27.1%). ^{1}H NMR (500 MHz, CD_3CN): δ 4.94 (1H, d, J=2.36 Hz), 5.16 (1H, d, J=2.36 Hz), 6.63 (1H, s), 7.07 (1H, m, J=3.92 Hz), 7.14 (2H, m, J=1.94 Hz), 7.32 (2H, m, J=2.68 Hz), 7.59 (2H, m, J=1.91 Hz), 7.70 (2H, m, J=3.25 Hz), 8.04 (1H, m, J=2.35 Hz), 8.30 (1H, q, J=3.02 Hz), 8.70 (2H, m, J=4.34 Hz) ppm. $^{13}C\{^{1}H\}$-NMR (125 MHz, $CDCl_3$): δ 29.85, 93.14, 93.85, 117.50, 120.90, 120.97, 122.70, 122.71,

123.31, 124.77, 125.58, 125.81, 125.99, 127.07, 127.09, 127.37, 127.49, 129.75, 132.75, 148.84, 156.34 ppm. HRMS (EI): m/z calculated for $C_{23}H_{16}O_3$, $[M]^+$ = 340.1094, found: 340.1091. Purity (HPLC): 95%.

Synthesis of 3-(4-(prop-2-yn-1-yloxy)phenyl)propanoic acid[2]

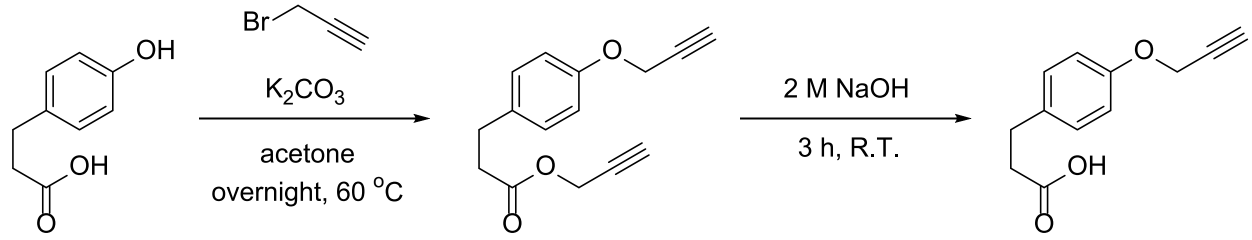

3-(4-hydroxyphenyl)propionic acid (12.0 mmol) and potassium carbonate (30 mmol, 2.5 equiv) was added to 50 mL round bottom flask. 15 mL acetone was added to the mixture. 80% wt propargyl bromide (30 mmol, 2.5 equiv) was added to the mixture dropwise and stirred at 60 °C overnight. The solvent mixture was removed *in vacuo*. The desired product was purified by flash column chromatography on silica gel by wet loading using 1:5 v/v ethyl acetate/hexane. The intermediate product was dissolved in MeOH and added with 2 M NaOH. The solution was stirred for 3 h at room temperature. Acidifying the solution with 37 % HCl would result in white precipitate. Addition of H_2O may aid in precipitating the desired product. The product was isolated using filtration.

TLC (1:5 v/v ethyl acetate/hexane), R*f*: 0.4

Yield: 1911.6mg (78.0%). ^{1}H NMR (500 MHz, $CDCl_3$): δ 2.51 (1H, t, J=2.40 Hz), 2.65 (2H, t, J=5.14 Hz), 2.91 (2H, t, J=7.72 Hz), 4.67 (2H, d, J=2.39 Hz), 6.91 (2H, m, J=8.72 Hz), 7.14 (2H, m, J=8.73 Hz) ppm. $^{13}C\{^1H\}$-NMR (100 MHz, $CDCl_3$): δ 29.85, 35.92, 55.98, 75.58, 78.79, 115.13, 129.39, 133.35, 156.24, 179.26 ppm.

Synthesis of 3-(4-(propa-1,2-dien-1-yloxy)phenyl)propanoic acid

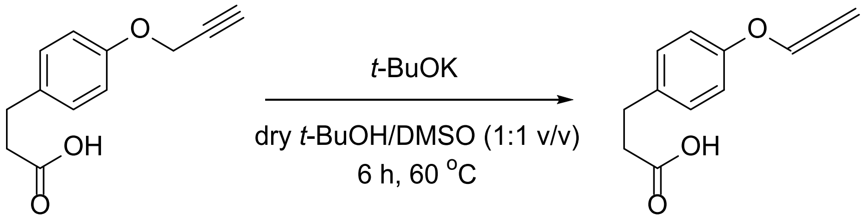

3-(4-(prop-2-yn-1-yloxy)phenyl)propanoic acid (2.5 mmol, 1.0 equiv), Potassium tert-butoxide (6.25 mmol, 5.5 equiv) were added to 50 mL two-neck round bottom flask. The reaction mixture was stirred at 60 °C for 6 h under the exclusion of moisture and air in anhydrous *t*-BuOH/DMSO (1:1 v/v, 10 mL). After the reaction, H_2O was added to the mixture and remaining *t*-BuOK will solubilize after 15 min. Quench the basic solution using saturated NH_4Cl. Perform liquid-liquid extraction using ethyl acetate with ample repeats. The combined organic phase was washed with brine twice to remove DMSO, dried with Na_2SO_4 drying agent and filtrate. The solvent mixture was removed *in vacuo* and resolubilized in ACN. The desired product was obtained after purification by HPLC using ACN and H_2O mobile phase. The collected HPLC fractions were combined and ACN was removed *in vacuo* (Heating above 42 °C may result in decomposition of the product). Perform liquid-liquid extraction using ethyl acetate with ample repeats again. The combined organic phase was washed with brine twice to remove DMSO, dried with Na_2SO_4 drying agent and filtrate. Hexane can be added sparingly to aid the precipitation of white solid product. The product was kept in -80 °C for long-term storage.

Yield: 209.8 mg (41.1 %). ^{1}H NMR (500 MHz, CD_3CN): δ 2.57 (2H, t, J=7.63 Hz), 2.85 (2H, t, J=7.62 Hz), 5.46 (2H, d, J=5.97 Hz), 6.95 (1H, t, J=5.97 Hz), 6.99 (2H, q, J=2.90 Hz), 7.19 (2H, d, J=8.75 Hz) ppm. ^{13}C{^{1}H}-NMR (75 MHz, D_2O): δ 31.00, 39.17, 90.14, 116.80, 117.78, 129.28, 136.71, 154.51, 182.45, 201.64 ppm. HRMS (EI): m/z calculated for $C_{12}H_{12}O_3$, $[M]^+$ = 204.0781, found: 204.0779.

Synthesis of 2,5-dioxopyrrolidin-1-yl 3-(4-(propa-1,2-dien-1-yloxy)phenyl)propanoate (APE-NHS)

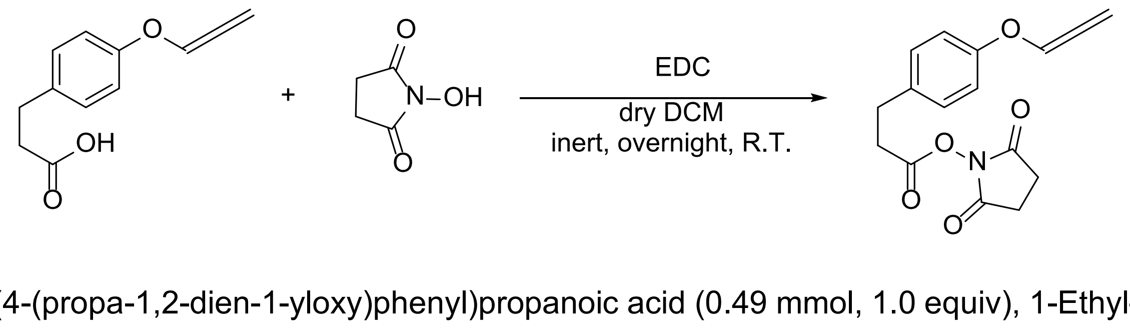

3-(4-(propa-1,2-dien-1-yloxy)phenyl)propanoic acid (0.49 mmol, 1.0 equiv), 1-Ethyl-3-(3-dimethylaminopropyl)carbodiimide (0.979 mmol, 2.0 equiv) and *N*-Hydroxysuccinimide (0.59 mmol, 1.2 equiv) were added into a 25 mL round bottom

flask. The reaction mixture was stirred overnight under the exclusion of moisture and air and anhydrous DCM (10 mL) was added, resulting in a clear solution. The solvent mixture was removed *in vacuo*. The desired product was obtained by flash chromatography using DCM as eluent. A white solid was obtained and kept in -80 °C for long-term storage.

TLC (1:5 v/v ethyl acetate/hexane), R*f*: 0.74

Yield: 112.1 mg (75.9 %). ^{1}H NMR (400 MHz, CD_3CN): δ 2.76 (4H, s), 2.93 (2H, t, J=3.28 Hz), 2.97 (2H, d, J=6.28 Hz), 5.47 (2H, d, J=5.96 Hz), 6.96 (1H, t, J=5.96 Hz), 7.01 (2H, m, J=4.31 Hz), 7.25 (2H, m, J=8.60 Hz) ppm. ^{13}C{^{1}H}-NMR (125 MHz, CD_3CN): δ 26.38, 30.14, 33.13, 41.30, 90.20, 117.60, 118.70, 130.64, 135.14, 156.74, 169.49, 171.10 ppm. HRMS (EI): m/z calculated for $C_{16}H_{15}NO_5$, $[M]^+$ = 301.0945, found: 301.0944. Purity (HPLC): > 99%.

Synthesis of 3-(4-((3-methylene-2,3-dihydrophenanthro[9,10-b][1,4]dioxin-2-yl)oxy)phenyl)propanoic acid

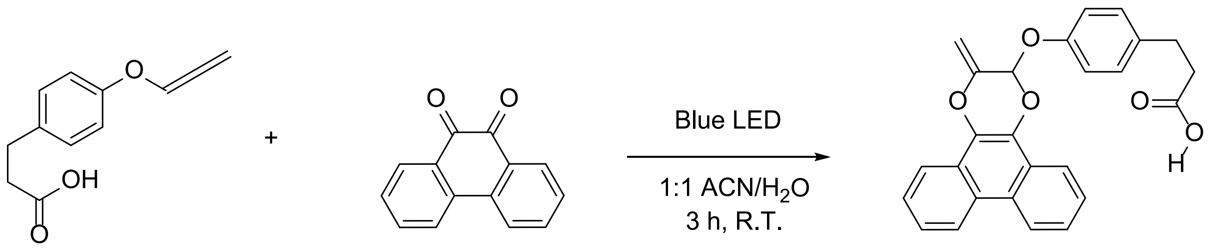

PQ (1.22 mmol, 1.20 equiv) and 3-(4-(propa-1,2-dien-1-yloxy)phenyl)propanoic acid (1.02 mmol) were dissolved in 500 mL 1:1 v/v acetonitrile/water. The mixture was stirred and irradiated with blue light LED strips for 180 min. The solvent mixture was removed *in vacuo*. The desired product was purified by flash column chromatography on silica gel by dry loading using eluent 1:4 v/v ethyl acetate/hexane.

TLC (1:2 v/v ethyl acetate/hexane), R*f*: 0.4

Yield: 91.3 mg (21.7%). ^{1}H NMR (400 MHz, CD_3CN) δ 2.59–2.54 (m, 2H), 2.85 (t, J = 8.0 Hz, 2H), 4.96 (d, J = 2.3 Hz, 1H), 5.17 (d, J = 2.3 Hz, 1H), 6.60 (s, 1H), 7.08 (d, J = 8.7 Hz, 2H), 7.20 (d, J = 8.7 Hz, 2H), 7.63 (dd, J = 6.2, 3.3 Hz, 2H), 7.73 (ddd, J = 14.7, 8.0, 1.3 Hz, 2H), 8.12–8.03 (m, 1H), 8.37–8.24 (m, 1H), 8.81–8.65 (m, 2H) ppm. ^{13}C{^{1}H}-NMR (100 MHz, CD_3CN) δ 30.34, 30.544, 35.806, 93.142, 94.884, 117.945,

121.235, 121.428, 123.865, 123.941, 125.319, 126.655, 126.823, 127.008, 128.066, 128.211, 128.264, 128.383, 130.42, 130.525, 133.43, 136.792, 149.729, 155.070, 174.270 ppm. HRMS (APCI): m/z calculated for $C_{26}H_{20}O_5$, $[M]^+$ = 412.1305, found: 412.1311.

Synthesis of 2,5-dioxocyclopentyl 3-(4-((3-methylene-2,3-dihydrophenanthro[9,10-b][1,4]dioxin-2-yl)oxy)phenyl)propanoate

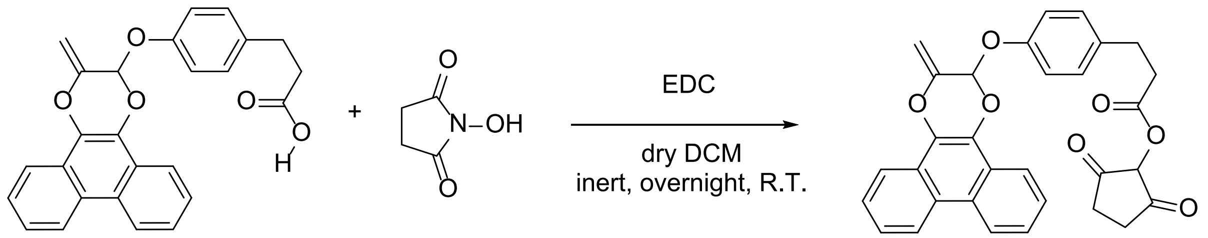

Active ester formation reaction with NHS is adapted from Staros et al.[3] 3-(4-((3-methylene-2,3-dihydrophenanthro[9,10-b][1,4]dioxin-2-yl)oxy)phenyl)propanoic acid (0.2 mmol, 1.0 equiv), 1-Ethyl-3-(3-dimethylaminopropyl)carbodiimide (0.4mmol, 2.0 equiv) and *N*-Hydroxysuccinimide (0.24 mmol, 1.2 equiv) were added into a 25 mL round bottom flask. The reaction mixture was stirred overnight under the exclusion of moisture and air and anhydrous DCM (5 mL) was added, resulting in a clear solution. The solvent mixture was removed *in vacuo*. The desired product was obtained by flash chromatography using hexane/ethyl acetate as eluent. An off-white solid is obtained and kept in -80 °C for long term storage.

Yield: 85.2mg (83.6%).^{1}H NMR (500 MHz, CD_3CN) δ 2.78 (s, 4H), 2.93 (tt, J = 6.5, 3.7 Hz, 2H), 3.02–2.96 (m, 2H), 4.96 (d, J = 2.4 Hz, 1H), 5.18 (d, J = 2.4 Hz, 1H), 6.62 (s, 1H), 7.07–7.15 (m, 2H), 7.22–7.31 (m, 2H), 7.59–7.68 (m, 2H), 7.74 (dddd, J = 26.4, 8.4, 7.0, 1.3 Hz, 2H), 8.04–8.12 (m, 1H), 8.29–8.38 (m, 1H), 8.69–8.81 (m, 2H) ppm. $^{13}C\{^1H\}$-NMR (125 MHz, CD_3CN) δ 26.39, 30.13, 30.35, 33.08, 93.09, 94.95, 118.00, 121.25, 121.46, 123.86, 123.95, 125.33, 126.65, 126.83, 127.02, 128.07, 128.23, 128.29, 128.39, 130.42, 130.69, 133.43, 135.47, 149.69, 155.33, 169.47, 171.07 ppm. HRMS (APCI): m/z calculated for $C_{30}H_{24}NO_7$, $[M+H]^+$ = 510.1547, found: 510.1551. Purity (HPLC): > 99%.

Synthesis of Rhodamine-N-piperazine-N-Boc[4]

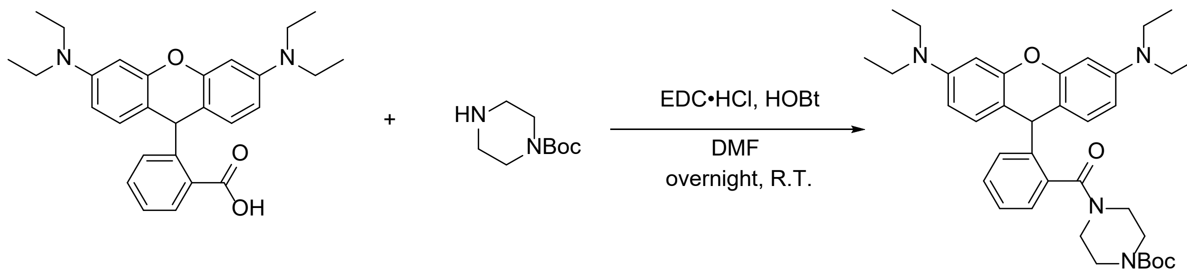

Rhodamine B (2.1 mmol, 1.0 equiv), EDC·HCl (4.2 mmol, 2.0 equiv), HOBt (4.2 mmol, 2.0 equiv) and 10 mL DMF were added to 50 mL round bottom flask. The solution was stirred for 180 min followed by addition of 1-Boc piperazine (3.2 mmol, 1.5 equiv). The reaction mixture was stirred at room temperature overnight. The reaction mixture was concentrated in vacuo. Perform liquid-liquid extraction using $CHCl_3$ and H_2O with ample repeats. The combined organic phase was washed with brine, dried with Na_2SO_4 drying agent and filtrate. The desired Rhodamine-N-piperazine-N-Boc was obtained by flash chromatography using EtOH/DCM (1:15 v/v).

Yield: 862.2mg (67%) ^{1}H NMR (400 MHz, acetone-d_6): δ 1.33 (12H, t, J=7.11 Hz), 1.40 (9H, s), 3.27 (8H, m), 3.78 (8H, q, J=7.13 Hz), 6.96 (2H, d, J=2.48 Hz), 7.18 (1H, d, J=2.44 Hz), 7.21 (1H, d, J=2.48 Hz), 7.32 (1H, s), 7.34 (1H, s), 7.57 (1H, m), 7.75 (3H, m) ppm. ^{13}C{^{1}H}-NMR (125 MHz, $CDCl_3$): δ 12.68, 28.32, 28.47, 41.78, 46.18, 46.94, 47.33, 80.60, 96.51, 109.35, 111.59, 113.76, 114.14, 120.32, 125.27, 125.41, 127.52, 128.98, 130.16, 130.53, 130.70, 132.02, 135.09, 154.41, 155.64, 157.74, 167.68 ppm.

Synthesis of Rho-PQ

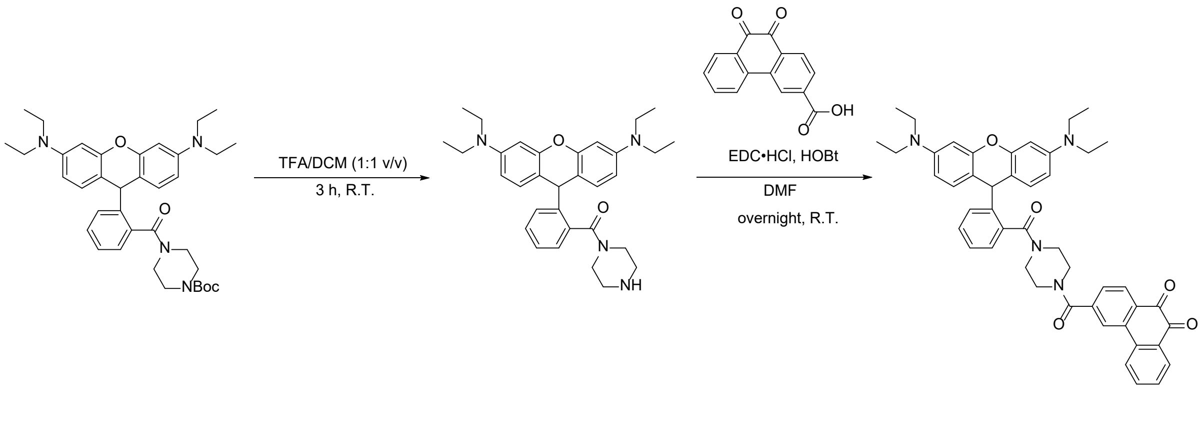

For Boc deprotection, Rhodamine-N-piperazine-N-Boc was added with 10 mL (1:1 v/v trifluoroacetic acid/DCM) and stir at room temperature for 3 h. The reaction mixture was concentrated *in vacuo* to remove the solvent and directly used in the next step without further purification. 9,10-Dioxo-9,10-dihydrophenanthrene-3-carboxylic acid (0.8 mmol, 1.0 equiv), EDC·HCl (1.6 mmol, 2.0 equiv), HOBt (1.6 mmol, 2.0 equiv), 10 mL DMF were added to 25 mL round bottom flask. The solution was stirred for 15 min followed by addition of deprotected Rhodamine-N-piperazine (excess). The reaction mixture was stirred at room temperature overnight. Perform liquid-liquid extraction using $CHCl_3$ thrice. The combined organic phase was washed with brine twice, dried with Na_2SO_4 drying agent and filtrate. The desired **Rho-PQ** was purified using HPLC with H_2O/ACN with 0.1 % TFA additive.

Yield: 383.6mg (64.2 %). ^{1}H NMR (500 MHz, DMSO-d_6): δ 1.23 (12H, t, J=7.10 Hz), 3.67 (8H, q, J=7.05 Hz), 6.94 (2H, d, J=2.30 Hz), 7.10 (2H, t, J=8.70 Hz), 7.17 (1H, s), 7.19 (1H, s), 7.46 (1H, d, J=7.85 Hz), 7.51 (1H, d, J=6.55 Hz), 7.58 (1H, t, J=7.48 Hz), 7.70 (1H, d, J=6.50 Hz), 7.78 (3H, m), 8.06 (1H, s), 8.07 (1H, s), 8.23 (1H, s), 8.30 (1H, d, J=7.95 Hz) ppm. ^{13}C{^{1}H}-NMR (125 MHz, CD_3CN): δ 12.82, 46.70, 96.95, 114.62, 115.11, 123.97, 125.66, 128.53, 130.70, 130.83, 130.93, 131.09, 131.19, 131.87, 132.39, 132.58, 132.96, 136.06, 136.43, 137.05, 137.29, 156.73, 158.79, 168.29, 169.24, 180.66, 180.76 ppm. HRMS (ESI): m/z calculated for $C_{47}H_{45}N_4O_5$, $[M-H]^+$ = 745.3384, found: 745.3391.

Synthesis of N-Boc-piperazyl-4’-TAMRA

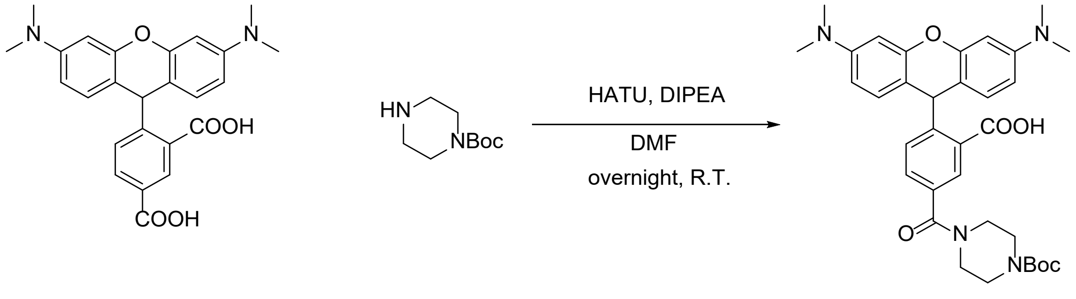

Slight modification was made to the following protocol.[5] 5-carboxy-tetramethylrhodamine (0.25 mmol, 1.0 equiv), HATU (0.25 mmol, 1.0 equiv), DIPEA (1.25 mmol, 5 equiv), 2 mL DMF were added into a 10 mL round bottom flask. The solution was stirred for 10 min followed by addition of 1-Boc piperazine (0.275 mmol,

1.1 equiv). The reaction mixture was stirred at room temperature overnight. The reaction mixture was concentrated in vacuo. The desired product was purified with DCM with 1 % triethylamine to 2 % MeOH in DCM with 1 % triethylamine. The product was reconstituted in 50 mL DCM, washed with aqueous NH_4Cl (3 × 20 mL), aqueous $NaHCO_3$ (5 % w/v, 3 × 20 mL), brine and dried with Na_2SO_4 drying agent and filter to obtain the N-Boc-piperazyl-4'-TAMRA.

Yield: 97.1 mg (64.3 %). ^{1}H NMR (500 MHz, $CDCl_3$): δ 1.48 (9H, s), 3.00 (12H, s), 3.49 (9H, m), 6.42 (1H, d, J=2.50 Hz), 6.43 (1H, d, J=2.50 Hz), 6.50 (2H, d, J=2.45 Hz), 6.63 (1H, s), 6.65 (1H, s), 7.23 (1H, d, J=3.88 Hz), 7.68 (1H, dd, J=1.25, 7.85 Hz), 8.01 (1H, d, J=0.45 Hz) ppm. ^{13}C{^{1}H}-NMR (125 MHz, $CDCl_3$): δ 28.51, 29.84, 40.44, 80.68, 98.48, 106.95, 109.35, 124.09, 125.43, 128.69, 129.13, 133.41, 136.93, 152.80, 153.48, 154.64, 168.80, 169.18 ppm.

Synthesis of TAMRA-PQ[6]

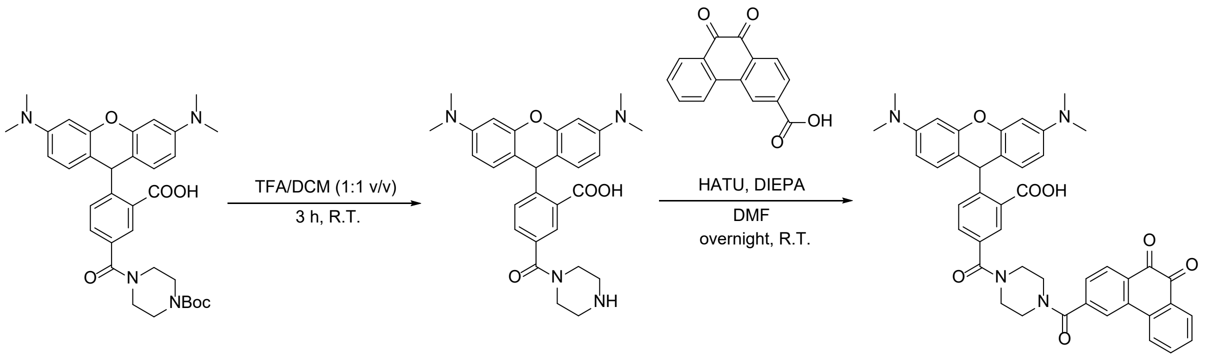

For Boc deprotection, N-Boc-piperazyl-4'-TAMRA was added with 10 mL (1:1 v/v trifluoroacetic acid/DCM) and stir at room temperature for 3 h. The reaction mixture was concentrated *in vacuo* to remove the solvent and directly used in the next step without further purification. 9,10-Dioxo-9,10-dihydrophenanthrene-3-carboxylic acid (0.2 mmol, 1.2 equiv), HATU (0.2 mmol, 1.2 equiv), DIPEA (0.84 mmol, 5 equiv), 1 mL DMF were added to 10 mL round bottom flask. The solution was stirred for 15 min followed by addition of deprotected piperazyl-4'-TAMRA (0.17 mmol, 1.0 equiv). The reaction mixture was stirred at room temperature overnight. The desired **TAMRA-PQ** was purified using HPLC with H_2O/ACN with 0.1 % TFA additive.

Yield: 17.7 mg (14.2 %). ^{1}H NMR (500 MHz, DMSO-d_6): δ 2.98 (12H, s), 3.61 (8H, m), 3.49 (9H, m), 6.57 (5H, bs), 7.35 (1H, bs), 7.57 (2H, t, J=7.47 Hz), 7.80 (2H, t, J=7.02

Hz), 8.07 (3H, m, J=8.01 Hz), 8.35 (1H, s), 8.40 (1H, d, J=7.98 Hz) ppm. HRMS (ESI): m/z calculated for $C_{44}H_{37}N_4O_7$, $[M-H]^+$ = 733.2657, found: 733.2660.

Photoligation between PQ and phenyl vinyl ether: 5 mM phenyl vinyl ether was incubated with 1 mM **PQ** in a H_2O/ACN (1:1 v/v) and subjected to blue LED light irradiation (18 W) for 20, 60 and 120 min. The reaction mixture was filtered using 0.22 μm filter and analyzed on HPLC, C18 column using H_2O/ACN elution system.

Photoligation between PQ and APE: Excess **APE** (10 equiv.) was incubated with 1 mM **PQ** in a H_2O/ACN (1:1 v/v) and subjected to blue LED light irradiation (18 W) for 20 min. The reaction mixture was filtered using 0.22 μm filter and analyzed on HPLC, C18 column using H_2O/ACN elution system.

Optimization studies for photoligation between PQ and APE, stoichiometric amount: 1, 2, 3, 4, 10 mM of **APE** was incubated with 2 mM **PQ** in deuterated D_2O/CD_3CN (1:1 v/v) and subjected to blue LED light irradiation (18 W) for 20 min. ^{1}H NMR was acquired after the reaction. The reaction mixture was filtered using 0.22 μm filter and analyzed on HPLC, C18 column using H_2O/ACN elution system.

Optimization studies for photoligation between PQ and APE, time-based: 1 mM of **APE** was incubated with 2 mM **PQ** in deuterated D_2O/CD_3CN (1:1 v/v) and subjected to blue LED light irradiation (18 W) for various intervals (2, 4, 6, 8, 10, 15 min). ^{1}H NMR was acquired after the reaction. The reaction mixture was filtered using 0.22 μm filter and analyzed on HPLC, C18 column using H_2O/ACN elution system.

Kinetic study of photoligation between PQ and APE: The rate constants of photoclick between **PQ** and **APE** were calculated based on ^{1}H NMR analysis. **PQ** (3 mM), **APE** (30 mM), and internal standard 1,3,5-trimethoxybenzene (1mM) were dissolved in CD_3CN/D_2O (1:1 v/v). The mixture was irradiated with blue LED strips (425-525 nm, 7 mW cm^{-2}). The photoligation kinetics was analysed based on the consumption rate of **PQ**. Pseudo-first order rate constant (k_{obs}) was extrapolated (k_{obs} = 1.49×10^{-2} s^{-1}) by plotting ln([PQ]) versus time. The second order rate constant (k_2) was calculated by k_{obs}/[APE] (k_2 = 0.50 M^{-1} s^{-1}).

Stability studies of allyl-PDO in pH 4-9: The pH of H_2O was tuned to pH 4, 7 and 9 using HCl and NaOH with a pH meter. 1 mM **allyl-PDO** was incubated for 24 h at 37 °C

in respective pH solution of THF/H_2O (1:1 v/v) using a thermomixer. The reaction mixture was filtered using 0.22 µm filter and analyzed on HPLC, C18 column using H_2O/ACN elution system.

Photophysical characterization of allyl-PDO adduct: Purified **allyl-PDO** was dissolved in ACN/H_2O (1:1 v/v) and measured for its absorbance profile using plate reader. The fluorescence emission profile was obtained by 310 nm excitation with steps of 5 nm interval.

Decaging studies on PDO: **PDO** was dissolved in THF (0.4 mM, 1 equiv.) and Na_2PdCl_4 were dissolved in either THF or PBS (0.8 mM, 2 equiv.). 200 µL of **PDO** was mixed with 200 µL of Na_2PdCl_4 species. Addition of respective THF or PBS solvent was introduced to achieve a final 800 µL solution of 1:1 v/v THF:PBS in 1.5 mL Eppendorf tubes. The mixture was incubated in thermomixer for 24 h at 37 °C, 750 rpm. After incubation, 400 µL of reaction mixture was added to 100 µL of 2.5 mM 4-methylumbelliferone as internal standard. The mix was filtered with 0.22 µm filter to remove undissolved solids and analyzed on HPLC with H_2O/ACN elution system.

Optimizing decaging on allyl-PDO through Pd stochiometric amount: **allyl-PDO** was dissolved in THF (0.4 mM) and respective Pd species were dissolved in either THF or PBS (1, 2, 4 equiv.). 200 µL of **allyl-PDO** was mixed with 200 µL of Pd species. Addition of respective THF or PBS solvent was introduced to achieve a final 800 µL solution of 1:1 v/v THF:PBS in 1.5 mL Eppendorf tubes. The mixture was incubated in thermomixer for 24 h at 37 °C, 750 rpm. After incubation, 400 µL of reaction mixture was added to 100 µL of 2.5 mM 4-methylumbelliferone as internal standard. The mix was filtered with 0.22 µm filter to remove undissolved solids and analyzed on UHPLC with H_2O/ACN elution system.

Optimizing decaging on allyl-PDO through additives: **allyl-PDO** was dissolved in THF (0.4 mM) and respective Pd species were dissolved in either THF or PBS/H_2O (2 equiv.). 200 µL of **allyl-PDO** was mixed with 200 µL of Pd species. Addition of morpholine, TPPTS and NaAsc (1 equiv) were supplemented. Addition of respective THF or PBS solvent was introduced to achieve a final 800 µL solution of 1:1 v/v THF:PBS in 1.5 mL Eppendorf tubes. The mixture was incubated in thermomixer for 24 h at 37 °C, 750 rpm. After incubation, 400 µL of reaction mixture was added to 100 µL of 2.5 mM 4-methylumbelliferone as internal standard. The mix was filtered with

0.22 µm filter to remove undissolved solids and analyzed on UHPLC with H_2O/ACN elution system.

Kinetic study of allyl-PDO deallylation mediated by Na_2PdCl_4: **Allyl-PDO** (1 mM), 4-methylumbelliferone (1mM) and Na_2PdCl_4 (1 mM) were dissolved in ACN/H_2O (1:1 v/v). The reaction mixture was incubated at 37 °C using a thermomixer. Samples were taken every 30 min and subjected to HPLC analysis. The deallylation kinetics was analysed based on the consumption rate of allyl-PDO. A linear correlation between ln([allyl-PDO]) and reaction time was observed. The first order rate constant was thus extrapolated ($k = 1.20 \times 10^{-4}\ s^{-1}$).

Cell viability assays of Na_2PdCl_4 and APE-NHS against HeLa cells*:* The cell viability assay was determined using WST8 assay*.* 10X Stock WST-8 (3 mg/mL, 5 mM) and 1-mPMS (0.2 mM) were prepared in PBS and filter sterilized. HeLa cells were seeded at 10,000 cells per well (100 µL) in Cellstar® 96-well plates (Greiner Bio-One) and incubated for 24 h. A control plate was assessed for the cell viability at timepoint t = 0 h where the nutrient media was replaced with 0.5 mM WST8 in RPMI and incubated for 1 h. For Na_2PdCl_4 and **APE-NHS** treatment, The RPMI nutrient media was replaced with Na_2PdCl_4 or **APE-NHS** in RPMI media at varying concentrations for 48 h in 37 °C incubator. After that, the nutrient media was replaced with 0.5 mM WST8 in RPMI and incubated for 1 h. The absorbance measured was 460 nm on plate reader. Each experiment was performed in 3 technical replicates and 3 biological replicates were carried out independently.

Emission studies on the deallylation of allyl-PDO and PDO mediated by Na_2PdCl_4: **Allyl-PDO** (1mM) or **PDO** (1mM) and Na_2PdCl_4 (1mM) were dissolved in ACN/H_2O (1:1 v/v) and incubated for 24 h at 37 °C using a thermomixer. The emission of **allyl-PDO** or **PDO** at 400 nm was monitored using microplate reader with excitation at 302 nm.

Fluorescence perturbation studies, Na_2PdCl_4 with rhodamine B/TAMRA: In a 96-well black plate, a final 200 µL solution of Na_2PdCl_4 concentrations from 10, 50, 100, 200 µM with 5 µM rhodamine B or TAMRA were dissolved in PBS. An additional 10 equivalent of NaAsc was added as necessary. The fluorescence intensity readout was measured after 24h incubation at ambient conditions.

In-gel fluorescence studies, photoligation of PQ with APE-lysozyme: 2 mg/mL **APE**-lysozyme in 1X PBS was added 20 equivalents of **PQ** (1:1 v/v 1X PBS:ACN) and topped with ACN to obtain a final solution mixture of 1:1 v/v 1X PBS:ACN in 6 well plate. Controls such as pure lysozyme, absence of **PQ** and absence of photoirradiation were included. Visible light irradiation was performed for 1 h at ambient conditions. Excess **PQ** was removed by dialysis (D9652-100FT, MWCO = 10,000) in 1X PBS overnight. The protein solution was stored at -20 °C when not in use. 30 µL of protein samples, 2 µL of 2 M DTT and 8 µL of 5 X Laemmli buffer were incubated in 95 °C for 5 min. The protein was analysed by gel electrophoresis using 15% polyacrylamide gels at 175 V and fixed for 30 min. The gel was imaged with GelDoc imaging system using excitation at 302 nm. Protein loading was compared by staining with Coomassie Brilliant Blue.

In-gel fluorescence studies, Pd-mediated decaging of allyl-PDO-lysozyme conjugated with PQ: 30 µL of **allyl-PDO**-lysozyme protein samples, 2 µL of 2 M DTT and 8 µL of 5 X Laemmli buffer were incubated in 95 °C for 5 min. The protein was analysed by gel electrophoresis using 15% polyacrylamide gels at 175 V and fixed for 30 min. The gel was imaged with GelDoc imaging system using excitation at 302 nm. The bands were cut accordingly and treated in conditions (20 mL 1X PBS, 20 mL of 1 mM NaAsc in 1X PBS, 20 mL of 100 µM Na_2PdCl_4 in 1X PBS, 20 mL of 100 µM Na_2PdCl_4 and 1 mM NaAsc in 1X PBS) and shaken for 24 h at room temperature. The gel was imaged with GelDoc imaging system using excitation at 302 nm. Protein loading was compared by staining with Coomassie Brilliant Blue.

In-gel fluorescence studies, photoligation of Rho-PQ with APE-lysozyme: 500 µM **Rho-PQ** (in DMF) was mixed with **APE**-lysozyme (1 mg/mL in PBS) and irradiated with visible light for 60 minutes. The protein solution was stored at -20 °C when not in use. 30 µL of protein samples, 2 µL of 2 M DTT and 8 µL of 5 X Laemmli buffer were incubated in 95 °C for 5 min. The protein was analysed by gel electrophoresis using 15% polyacrylamide gels at 175 V and fixed for 30 min. The gel was imaged using Typhoon at an excitation wavelength of 532 nm. Protein loading was compared by staining with Coomassie Brilliant Blue. Coomassie Brilliant Blue stained gel was imaged with GelDoc.

Photoligation and decaging on peptide model KVFGR: 7.5 mM **APE-NHS** ester or **allyl-PDO**-NHS ester stock solution was prepared in DMSO. Peptide KVFGR sample was dissolved in PBS to make 2 mM stock. 40 µL of NHS ester stock was mixed with 50 µL KVFGR peptide and topped up with PBS to 1 mL. Final concentration was 4%DMSO in PBS, 0.1 mM KVFGR and 0.3 mM NHS ester. The reaction mixture was allowed to stir overnight at room temperature. **APE-KVFGR** and **allyl-PDO-KVFGR** synthesized was analysed by LC-MS and used directly for next step without further purification.

PQ stock solution was prepared in DMSO at 7 mM. Ligation reaction was performed by mixing 500 µL **APE-KVFGR**, 50 µL **PQ** stock solution, and 450 µL acetonitrile. Final concentration was 0.05 mM **APE-KVFGR**, 0.35 mM **PQ** in ACN/PBS (1:1 v/v). The mixture was stirred and irradiated with blue LED strips for 60 mins.

0.8 mM Na_2PdCl_4 stock solution was prepared in PBS. Decage reaction was performed by mixing 500 µL Na_2PdCl_4 stock with 500 µL **allyl-PDO-KVFGR** solution. Final concentration was 2% DMSO in PBS, 0.4 mM Na_2PdCl_4 with 0.05 mM **allyl-PDO-KVFGR.** Reaction tube was put in shaking incubator for 24 h at 37 °C. All reaction mixtures were filtered with 0.22µm hydrophilic filter prior to LCMS analysis.

Confocal laser scanning microscopy, photoligation: A treated cover slip was put on the bottom of each well for confocal microscopy. HeLa cells were seeded at 200,000 cells per well (2 mL) in Cellstar® 6-well plates (Greiner Bio-One) and incubated for 24 h. Afterwards, the media was removed and the cells were washed with 1X PBS twice. The 5 mM **APE-NHS** stock solution in DMSO was diluted into a 50 µM solution (1 % DMSO in HBSS with Mg^{2+} and Ca^{2+} ions) for cell treatment at 37 °C for 30 min. The treated cells were washed with 1X PBS twice. The stock solution of 2 mM **Rho-PQ** in DMF was diluted into 20 µM (1 % DMF in RPMI) for cell treatment. Cells were exposed to visible light (380-800 nm, 15 mW cm^{-2}) for 1 h, followed by washing with 1X PBS twice. Afterwards, the cells were fixed with 1 mL 4% paraformaldehyde for 10 min and washed with 1X PBS twice. Hoechst 33342 (1 µg mL^{-1} in 1X PBS) was administered to the cells for 10 min as the control fluorophore staining. The treated cells were washed with 1X PBS twice. The cover slip was mounted and samples were stored at 4 °C overnight in the dark before imaging with Olympus FV1000. **Rho-PQ** was imaged with the red channel (λ_{ex} 559 nm/λ_{em} 577 nm)

while Hoechst 33342 was imaged with the blue channel (λ_{ex} 405 nm/λ_{em} 461 nm). Quantification of fluorescence intensity was performed using ImageJ. Unpaired T test was performed using GraphPad Prism 10 software (GraphPad Software Inc., CA) with $p < 0.05$ considered as significant (* $p < 0.05$, ** $p < 0.01$, *** $p < 0.001$, ns – not significant).

Confocal laser scanning microscopy, Pd-mediated decaging: A treated cover slip was put on the bottom of each well for confocal microscopy. HeLa cells were seeded at 200,000 cells per well (2 mL) in Cellstar® 6-well plates (Greiner Bio-One) and incubated for 24 h. Afterwards, the media was removed and the cells were washed with 1X PBS twice. The 5 mM **APE-NHS** stock solution in DMSO was diluted into a 50 µM solution (1 % DMSO in HBSS with Mg^{2+} and Ca^{2+} ions) for cell treatment at 37 °C for 30 min. The treated cells were washed with 1X PBS twice. Stock solutions of 2 mM **Rho-PQ** or **TAMRA-PQ** in DMF was diluted into 20 µM solution (1 % DMF in RPMI) for cell treatment. The cells with exposed with visible light (380-800 nm, 15 mW cm^{-2}) for 1 h followed by washing with 1X PBS twice. Rhodamine 123 (5 µM in 1X PBS) was administered to the cells for 10 min as the control fluorophore staining. The stained cells were washed with 1X PBS twice and fixed with 1 mL 4% paraformaldehyde for 10 min. The cells were washed with 1X PBS twice and incubated with Na_2PdCl_4 (100 µM) with or without NaAsc (1.0 mM) for 24 h at 37 °C. The treated cells were washed with 1X PBS twice. The cover slip was mounted and samples were stored at 4 °C overnight in the dark before imaging with Olympus FV1000. **Rho-PQ** and **TAMRA-PQ** were imaged with the red channel (λ_{ex} 559 nm/λ_{em} 577 nm). Rhodamine 123 was imaged with the green channel (λ_{ex} 473 nm/λ_{em} 519 nm). Quantification of fluorescence intensity was performed using ImageJ. Unpaired T test was performed using GraphPad Prism 10 software (GraphPad Software Inc., CA) with $p < 0.05$ considered as significant (* $p < 0.05$, ** $p < 0.01$, *** $p < 0.001$, ns – not significant).

Table S1. Selected X-ray Crystallographic Data for **allyl-PDO**

Complex	**allyl-PDO**
formula	$C_{23}H_{16}O_3$
formula weight	340.36
Temperature [K]	100(2)
wavelength [Å]	1.54178
Crystal size [mm^3]	0.188 × 0.067 × 0.062
Crystal system	Monoclinic
Space group	$P2_1/c$
a [Å]	4.5771(2)
b [Å]	17.6569(6)
c [Å]	40.1390(13)
a [deg]	90
β [deg]	90.928(2)
γ [deg]	90
V [$Å^3$]	3243.5(2)
Z	8
D_c [Mg/m3]	1.394
μ [mm^{-1}]	0.738
final R indices [[$I > 2\sigma(I)$]	*R*1 = 0.0358, *wR*2 = 0.0852
R indices (all data)	*R*1 = 0.0440, *wR*2 = 0.0905
goodness-of-fit on F^2	1.038
peak/hole [e Å-3]	0.189 and -0.239

[a]$R = \Sigma // F_o| - |F_c // /\Sigma|F_o|$, $wR2 = \Sigma\{[w(F_o^2 - F_c^2)^2]/\Sigma[w(F_o^2)^2]\}^{1/2}$. Goodness-of-fit (GOF) = $[\Sigma[w(F_o^2 - F_c^2)^2]/(n - p)]^{1/2}$, where *n* is the number of data and *p* is the number of parameters refined.

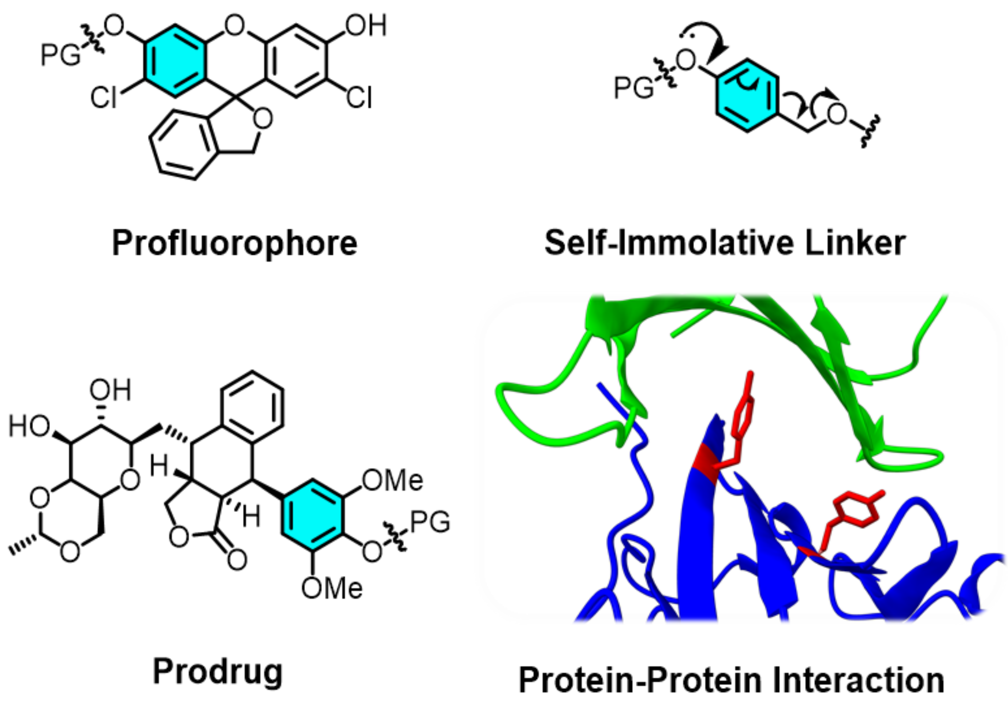

Figure S1. Essential phenolic motifs are ubiquitous in many scaffolds, for example, Pittsburgh Green, etoposide, p-hydroxyl benzyl linker, and PD-L1/PD-1 protein-protein interactions.

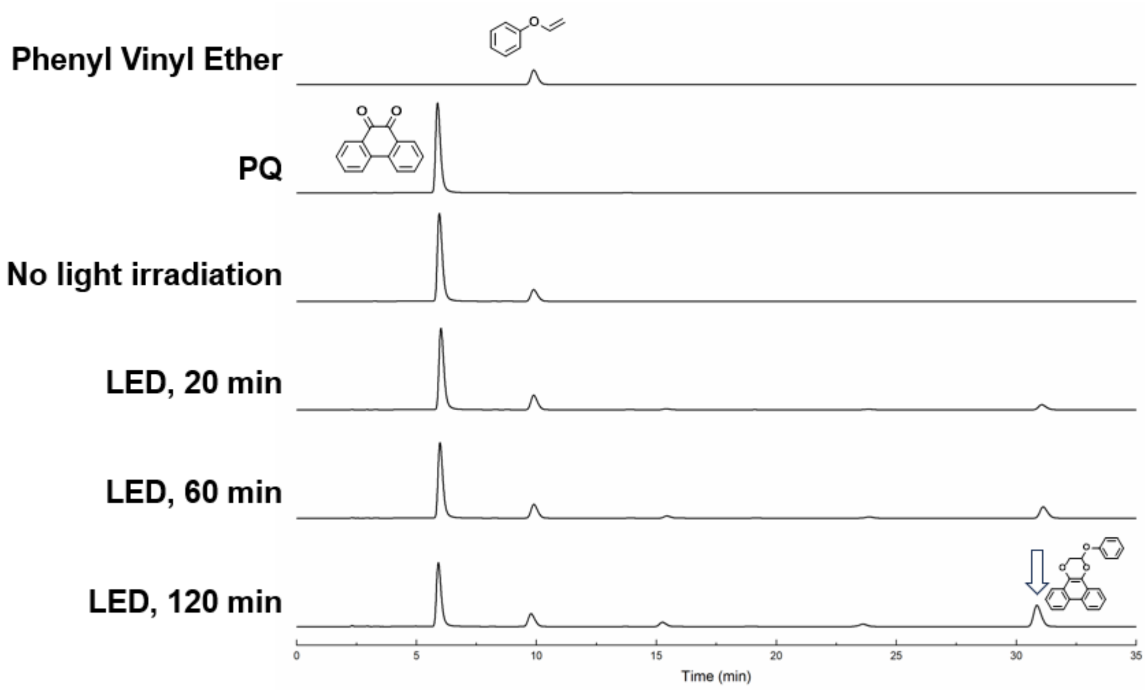

Figure S2. 2-phenoxyphenanthrodioxine (**PDO**) cycloadduct characterised by HPLC.

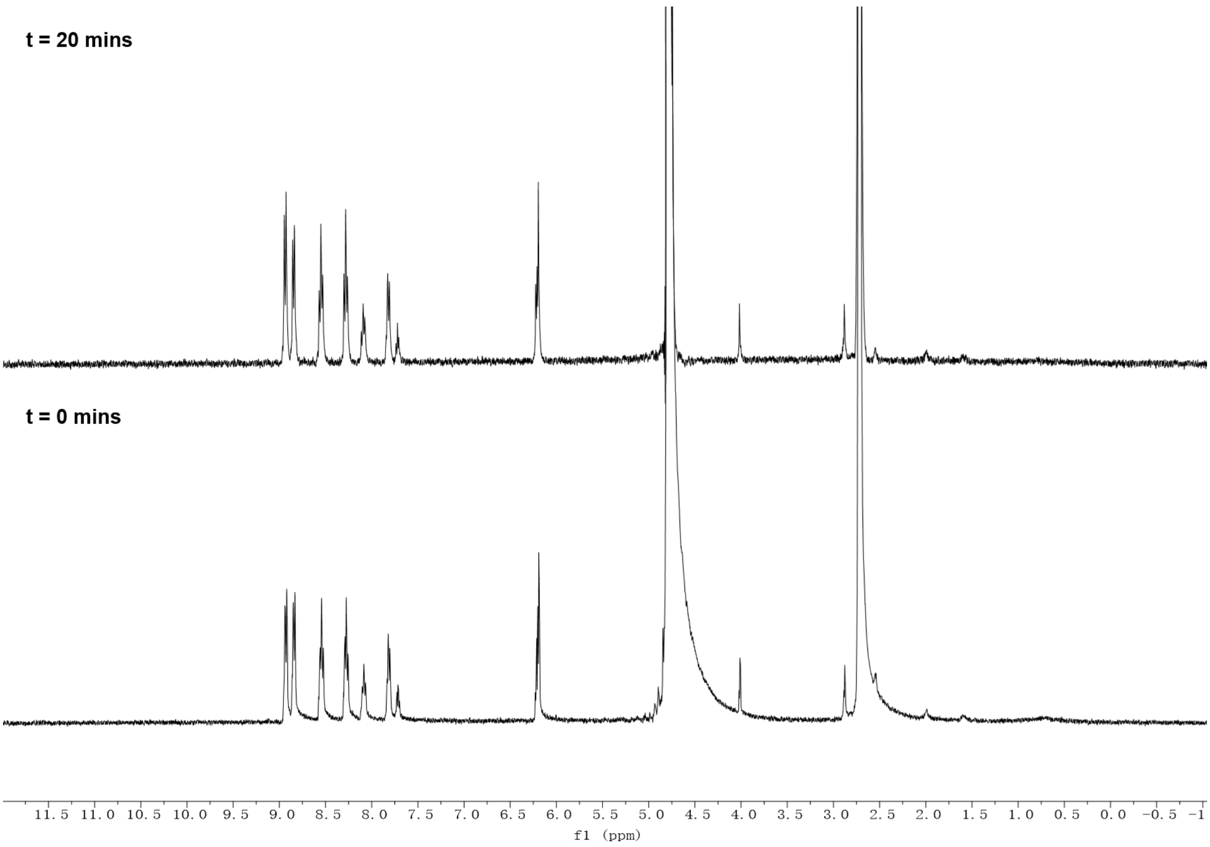

Figure S3. ^{1}H NMR analysis of photoligation between 2 mM **PQ** and 1 mM **APE** in 1:1 v/v CD_3CN/D_2O without light irradiation.

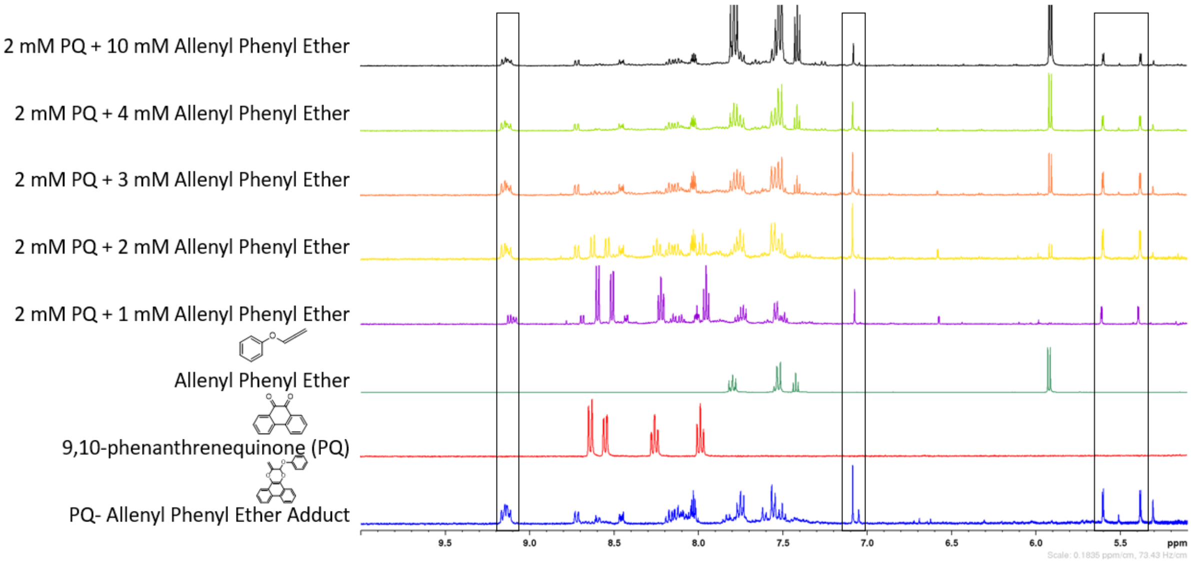

Figure S4. Photoligation of **PQ** with different loadings of **APE**.

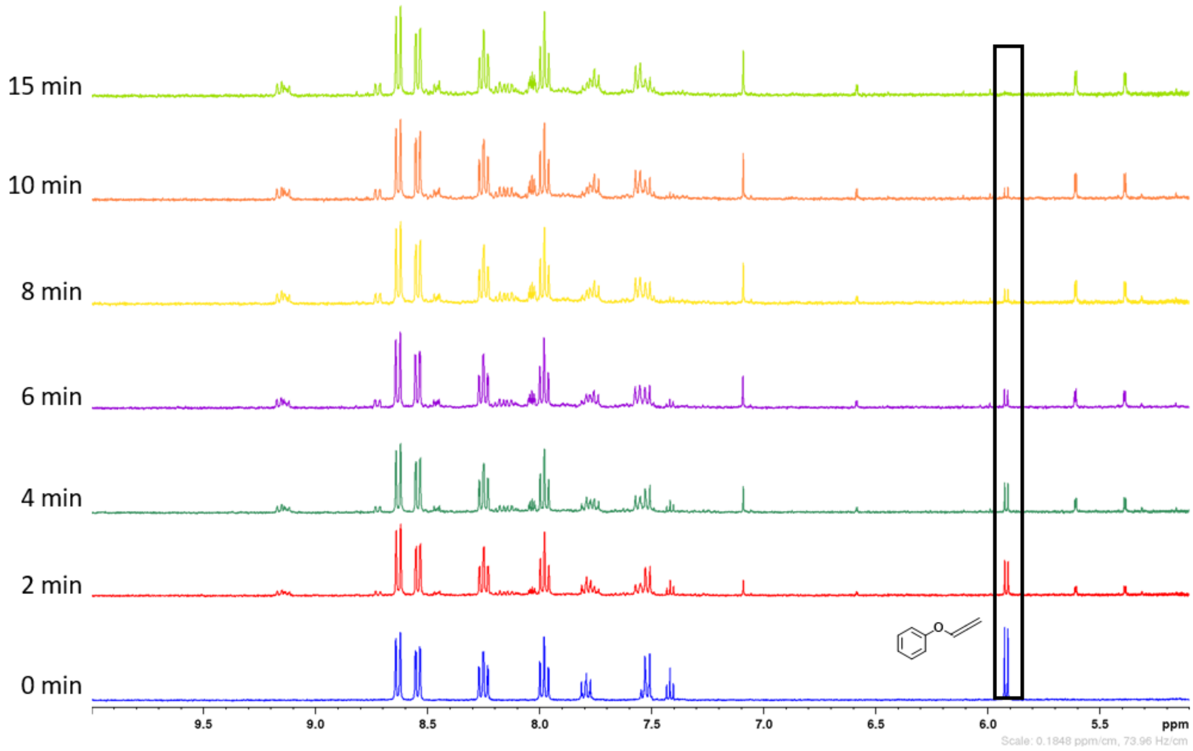

Figure S5. Time-dependent ^{1}H NMR analysis of the photoligation reaction between **APE** and **PQ** in 1:1 v/v CD_3CN/D_2O.

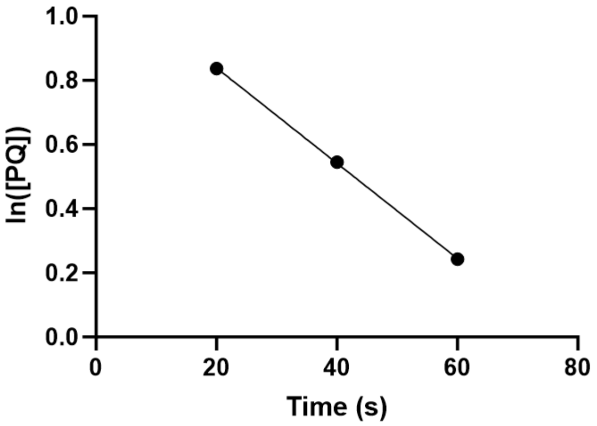

Figure S6. A linear correlation between ln([**PQ**]) and reaction time in the photoligation between **PQ** and **APE**, determined by ^{1}H NMR.

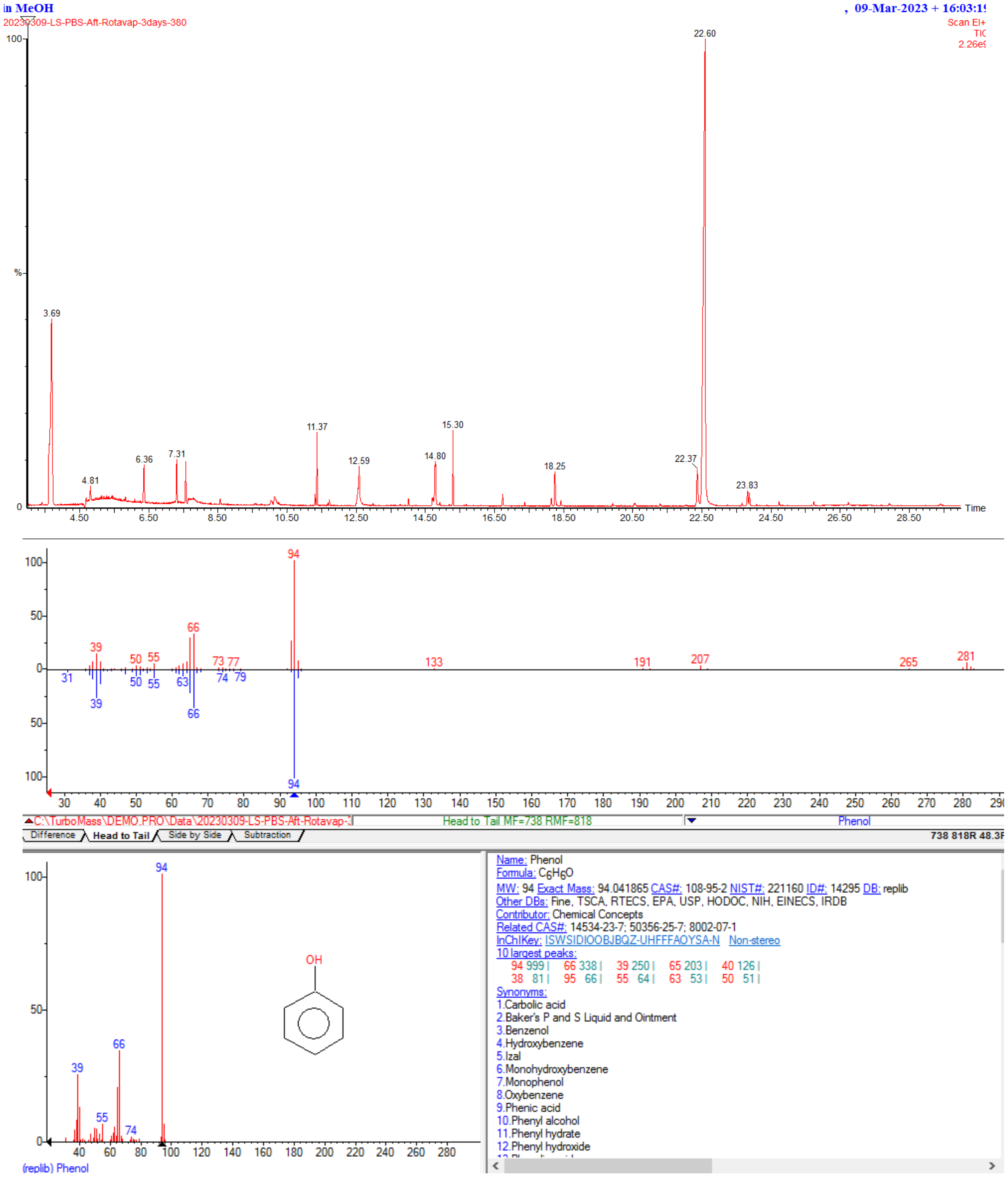

Figure S7. GC-MS analysis of the release of phenol when subjecting **allyl-PDO** to Na_2PdCl_4.

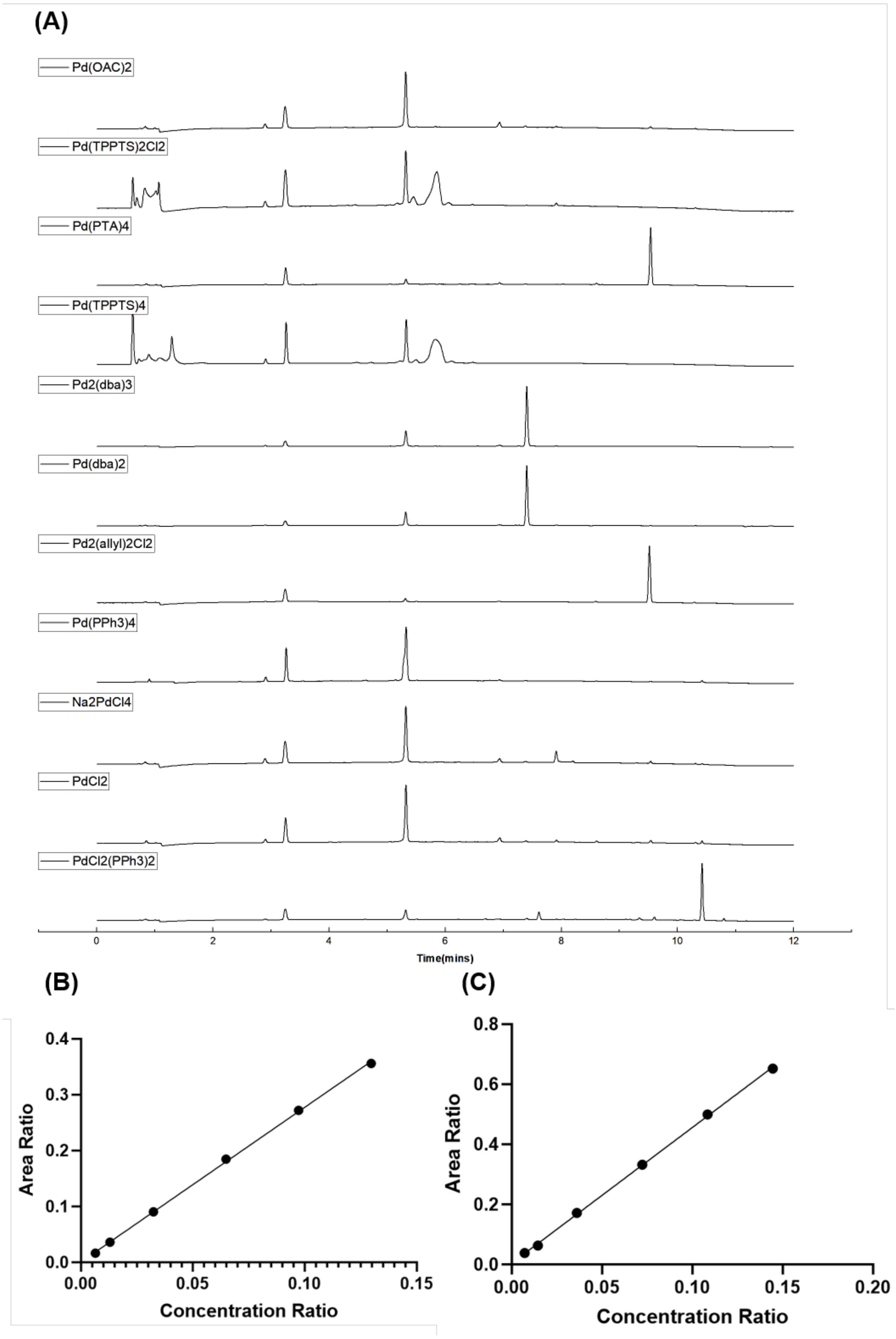

Figure S8. (A) HPLC analysis of **allyl-PDO** deallylation with different Pd-based catalysts. (B) Standard curve for phenol yield determination in THF/H_2O (1:1 v/v). (C) Standard curve for phenol yield determination in THF/PBS (1:1 v/v).

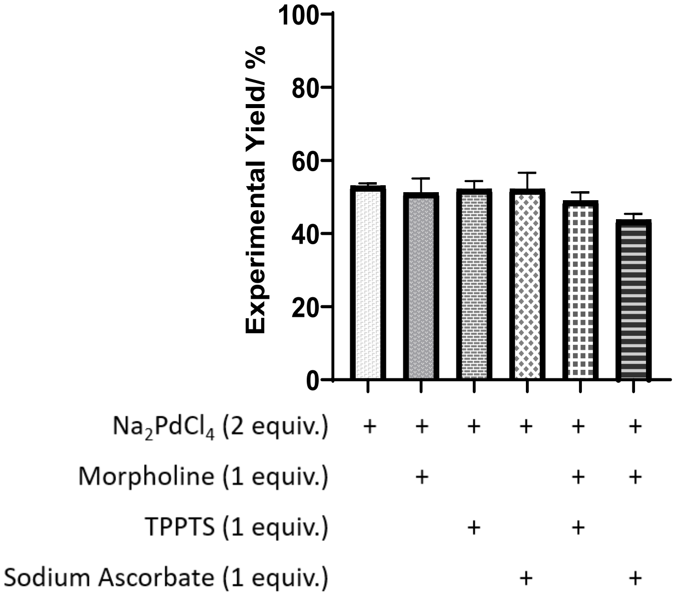

Figure S9. Yield of released phenol from **allyl-PDO** with corresponding additives co-incubated with Na_2PdCl_4.

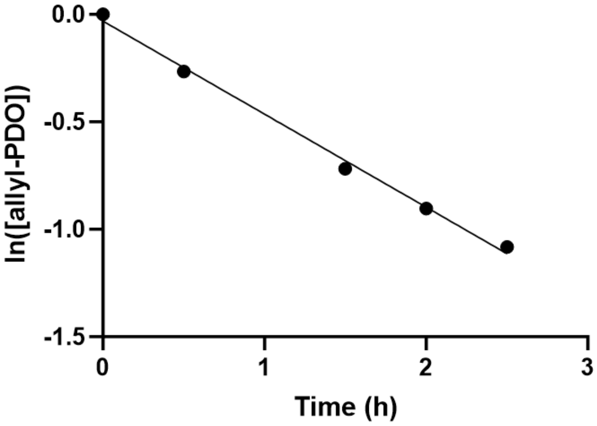

Figure S10. A linear correlation between ln([**allyl-PDO**]) and reaction time in the deallylation of **allyl-PDO** mediated by Na_2PdCl_4, determined by HPLC.

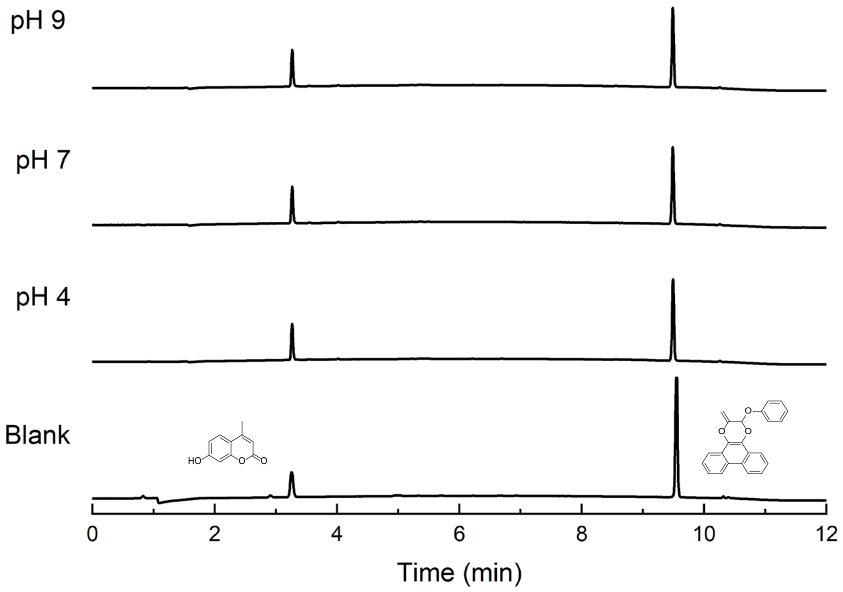

Figure S11. Stability test of **allyl-PDO** at different pH.

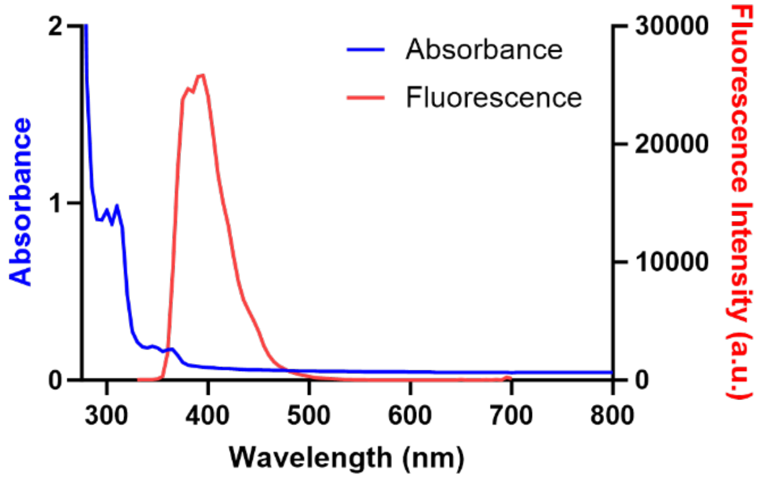

Figure S12. Absorption and Emission profiles of **allyl-PDO**.

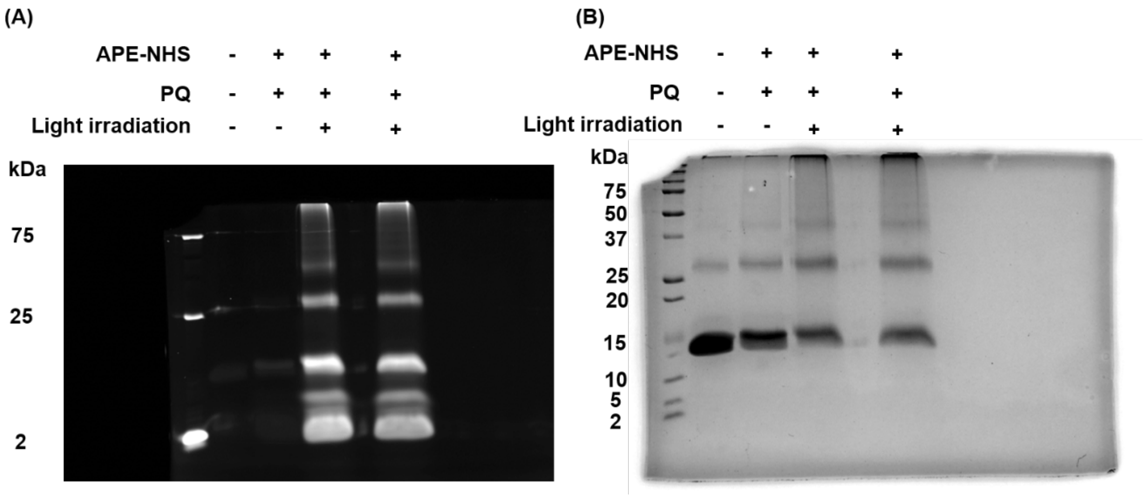

Figure S13. Uncropped (A) In-gel fluorescence and (B) Coomassie Brilliant Blue Stain for main text **Figure 3B**.

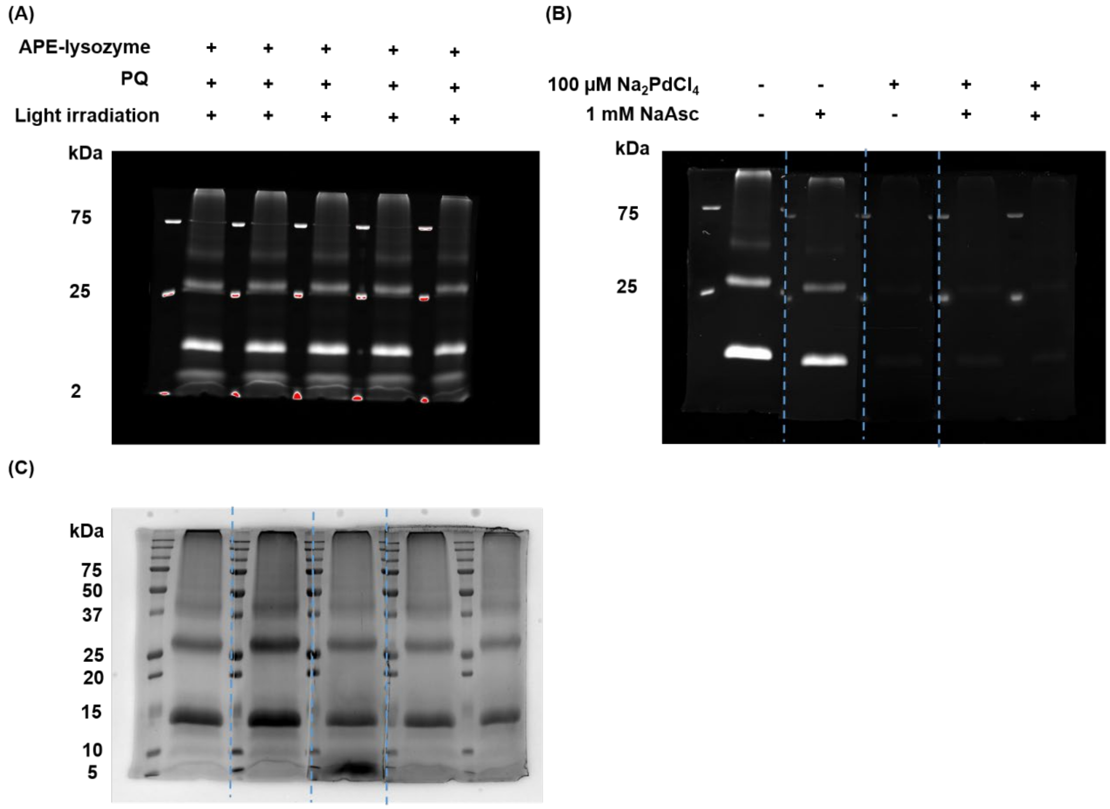

Figure S14. Uncropped images for main text **Figure 3C.** (A) In-gel fluorescence analysis of photoligation between **APE**-lyzozyme and **PQ**. (B) In-gel fluorescence analysis of **allyl-PDO-**lysozyme treated with/without Na_2PdCl_4 and/or NaAsc. As indicated by the blue dotted lines, the gel in (A) was cut into four pieces, each treated under different deallylation conditions, and then placed side by side for imaging. (C) Coomassie Brilliant Blue Stain.

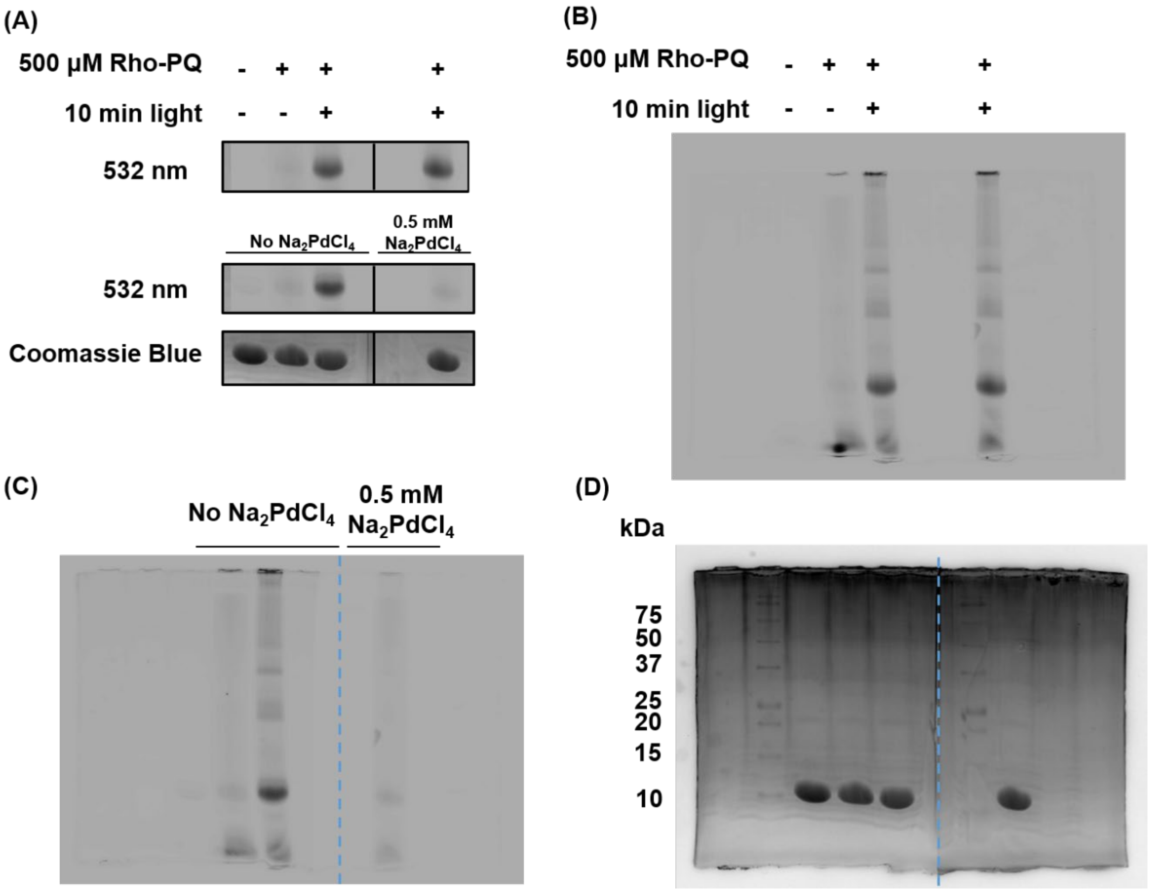

Figure S15. (A) In-gel fluorescence analysis of the photoligation and deallylation reaction. **Rho-PQ** was mixed with **APE**-lysozyme to form **allyl-PDO** under light irradiation, imaged using GelDoc at 532 nm excitation channel. The gel was cut into two pieces and subjected to further deallylation reaction with Na_2PdCl_4. Equal protein loading was confirmed by Coomassie-stained gel. (B) Uncropped in-gel fluorescence analysis of photoligation reaction. (C) Uncropped in-gel fluorescence analysis of deallylation reaction. As indicated by the blue dotted lines, the gel in (B) was cut into two pieces, treated with or without Na_2PdCl_4 and then placed side by side for imaging. (D) Coomassie Brilliant Blue Stain.

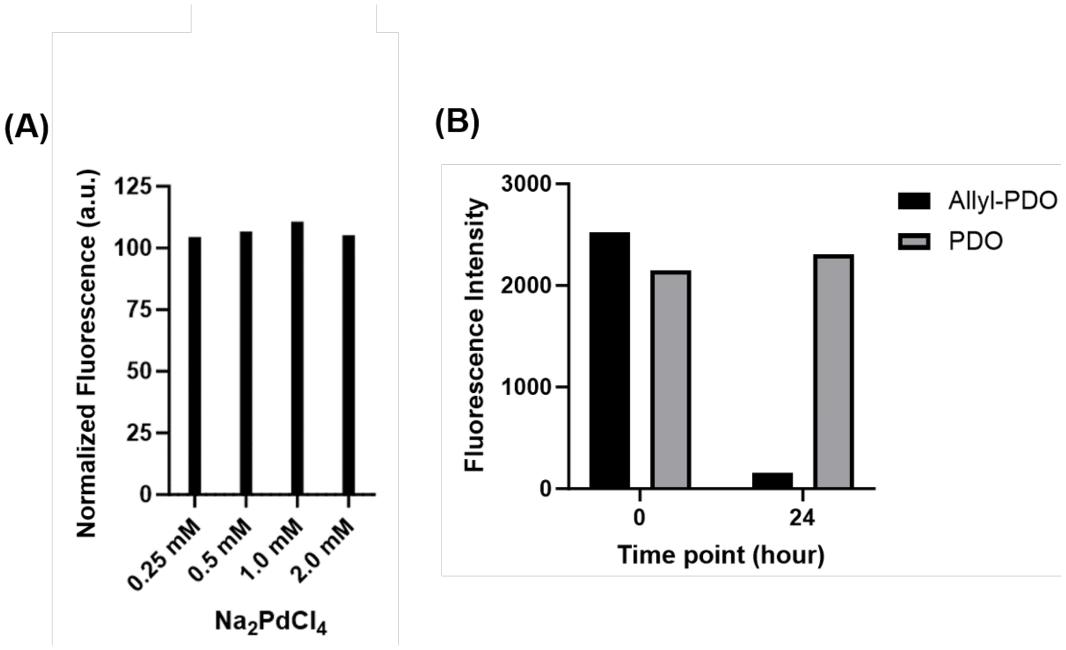

Figure S16. (A) Stability test of **PDO** (1 mM) treated with 0.25-2 mM Na_2PdCl_4. Fluorescence was measured by microplate reader at 400 nm channel with excitation at 302 nm. Data was normalized to blank with no addition of Na_2PdCl_4. (B) Emission study on the deallylation of **allyl-PDO** (1mM) and **PDO** (1mM) using Na_2PdCl_4 (1mM) for 24 h incubation using microplate reader at 400 nm channel with excitation at 302 nm.

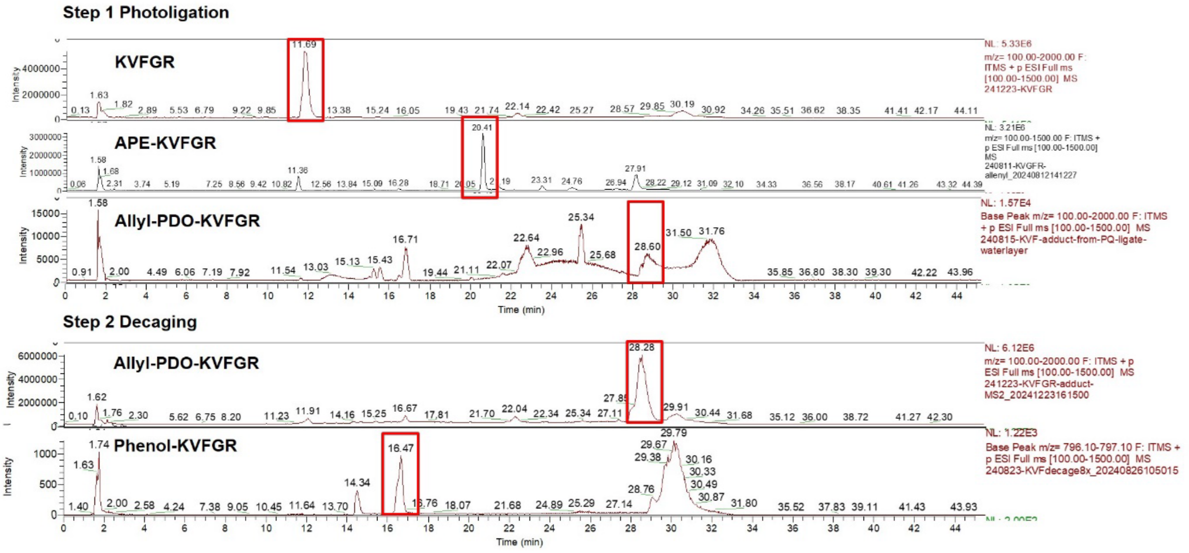

Figure S17. LC-MS results of click and release on peptide **KVFGR**. Peaks corresponding to each modified **KVFGR** were marked with red boxes.

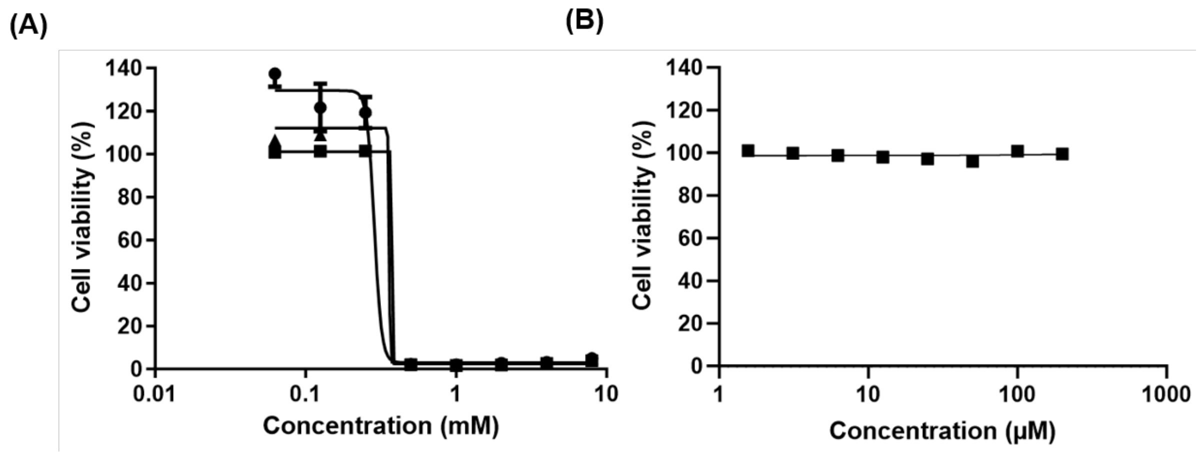

Figure S18. Cell viability assays of (A) Na_2PdCl_4 and (B) **APE-NHS** against HeLa cells.

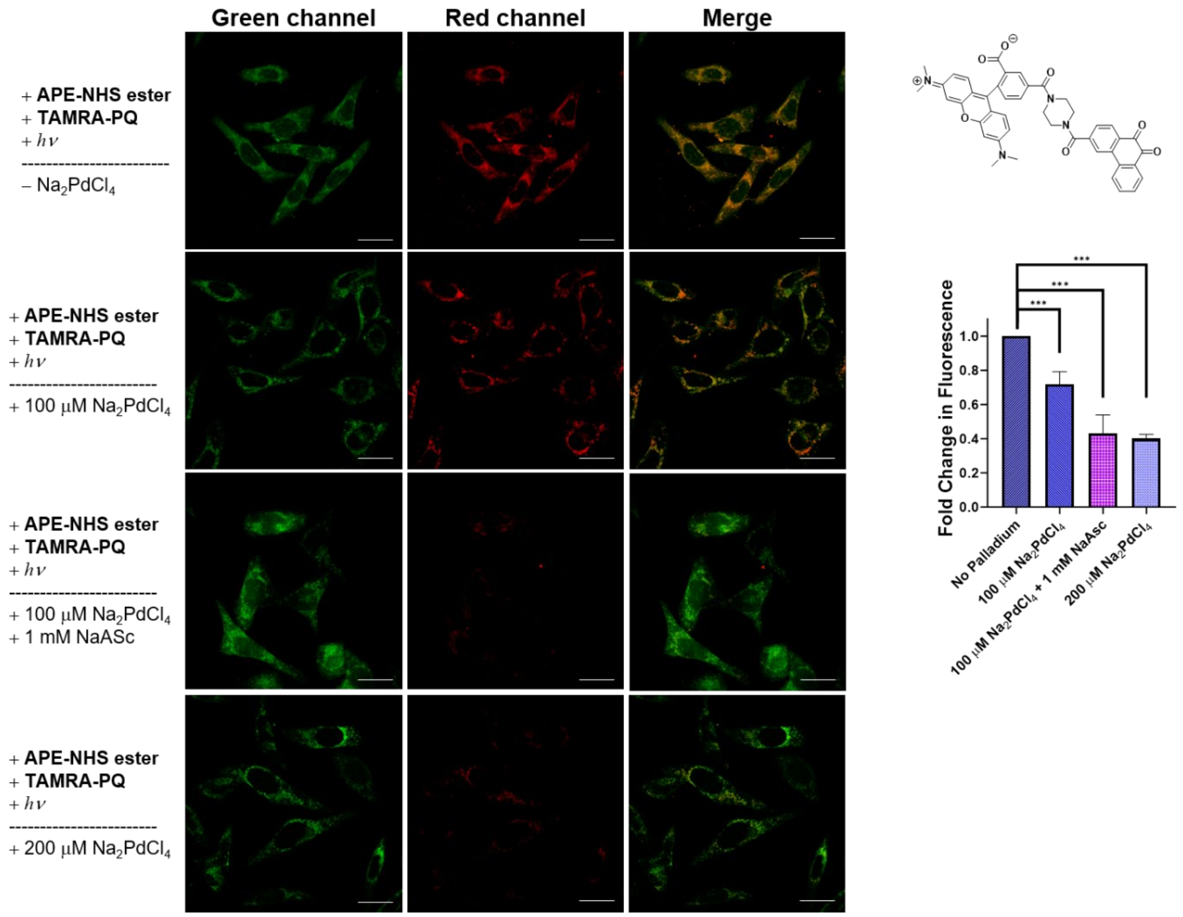

Figure S19. CLSM images of cellular deallylation when adducts of TAMRA-conjugated **PQ** (**TAMRA-PQ**) and **APE** treated with 100 μM Na_2PdCl_4, 100 μM Na_2PdCl_4 and 1 mM NaAsc, 200 μM Na_2PdCl_4. Mean fold-change in fluorescence intensities upon Pd administration to HeLa cells quantified by ImageJ software. Control staining using rhodamine 123 (λ_{ex} 473 nm/λ_{em} 519 nm) was viewed with the green channel. **TAMRA-PQ** (λ_{ex} 559 nm/λ_{em} 577 nm) was viewed with the red channel. Scale bar: 20 μm.

Unpaired T test was performed using GraphPad Prism 10 software with $p < 0.05$ considered as significant (* $p < 0.05$, ** $p < 0.01$, *** $p < 0.001$).

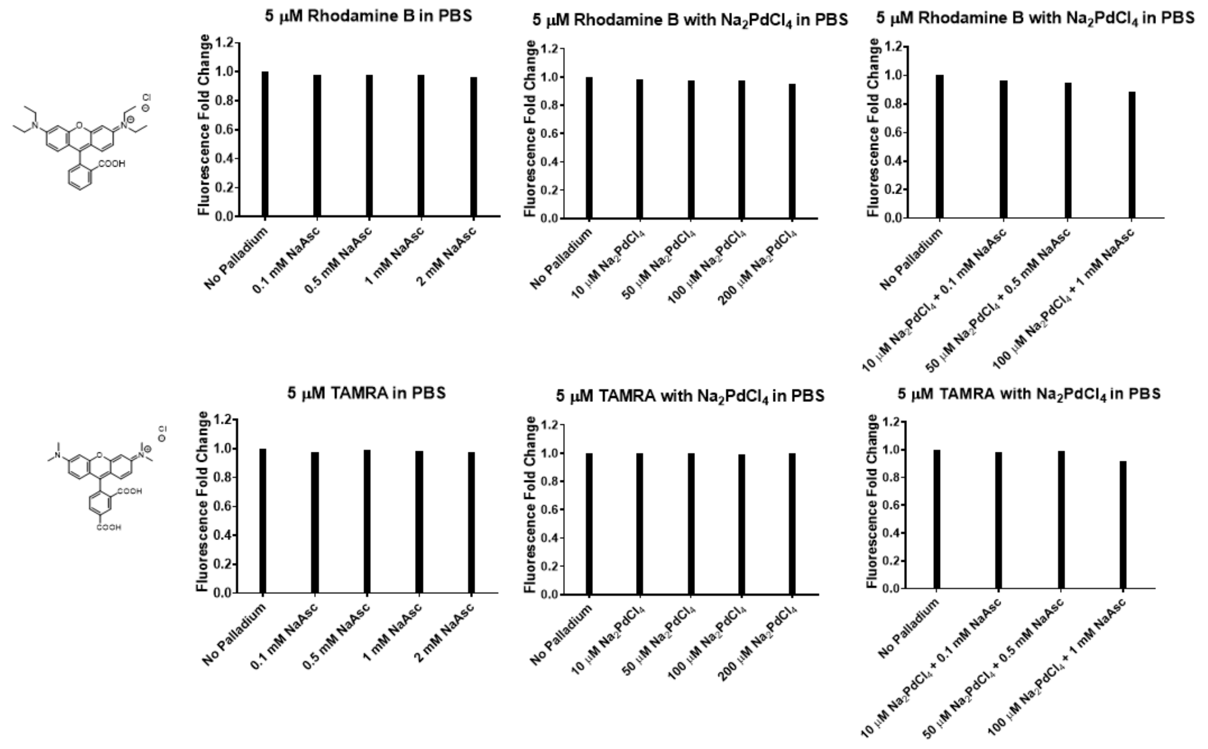

Figure S20. Stability test of **Rhodamine B** and **TAMRA** (5 μM) treated with 10-200 μM Na_2PdCl_4 and 0.1-2 mM NaAsc for 24 h at ambient conditions. Fluorescence was measured by microplate reader at 532 nm channel with excitation at 302 nm. Data was normalized to blank with no addition of Na_2PdCl_4 or NaAsc.

^{1}H NMR and $^{13}C\{^{1}H\}$ NMR spectra of synthesized compounds

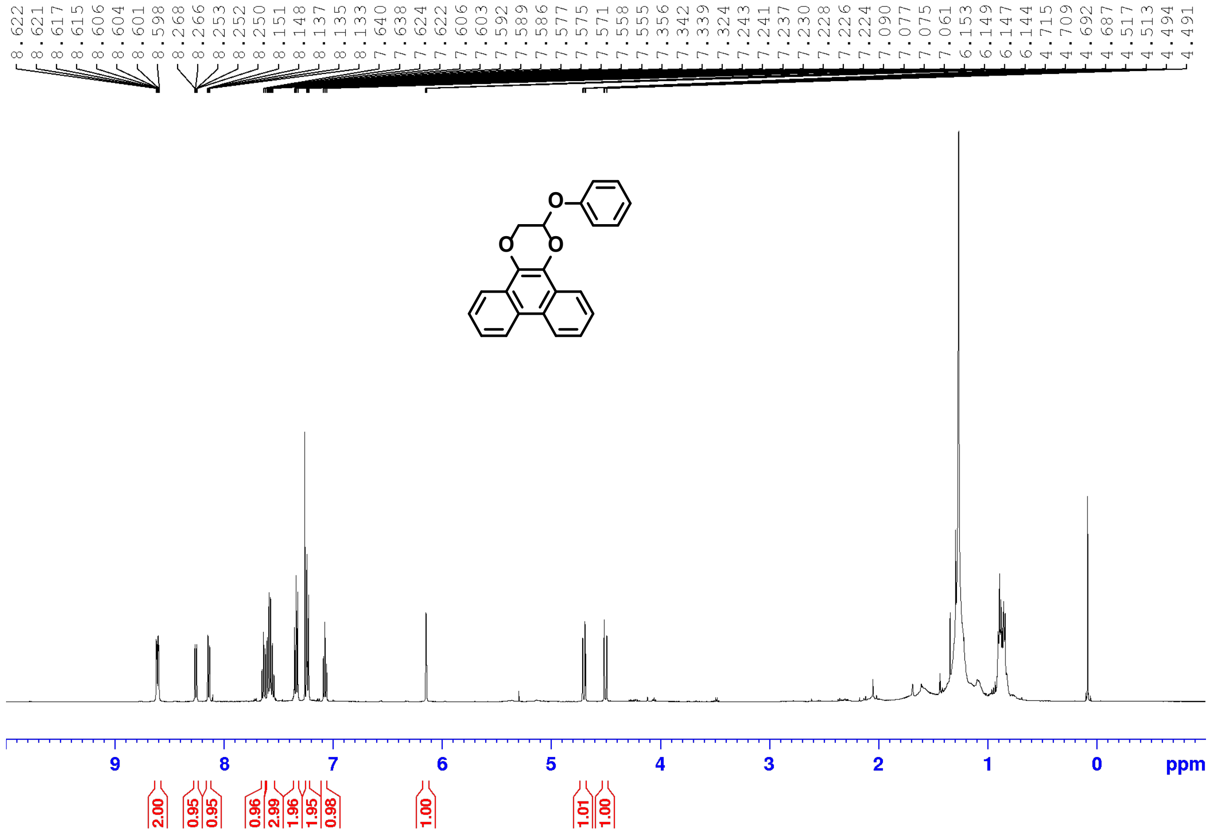

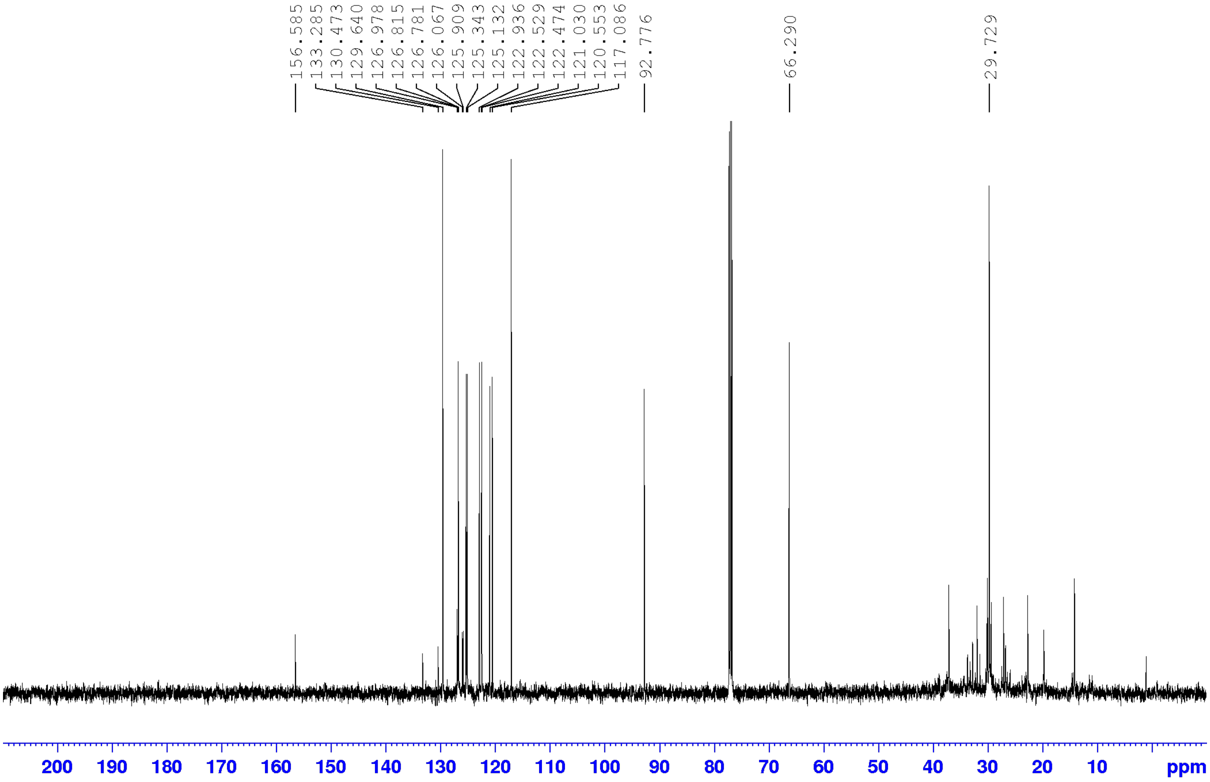

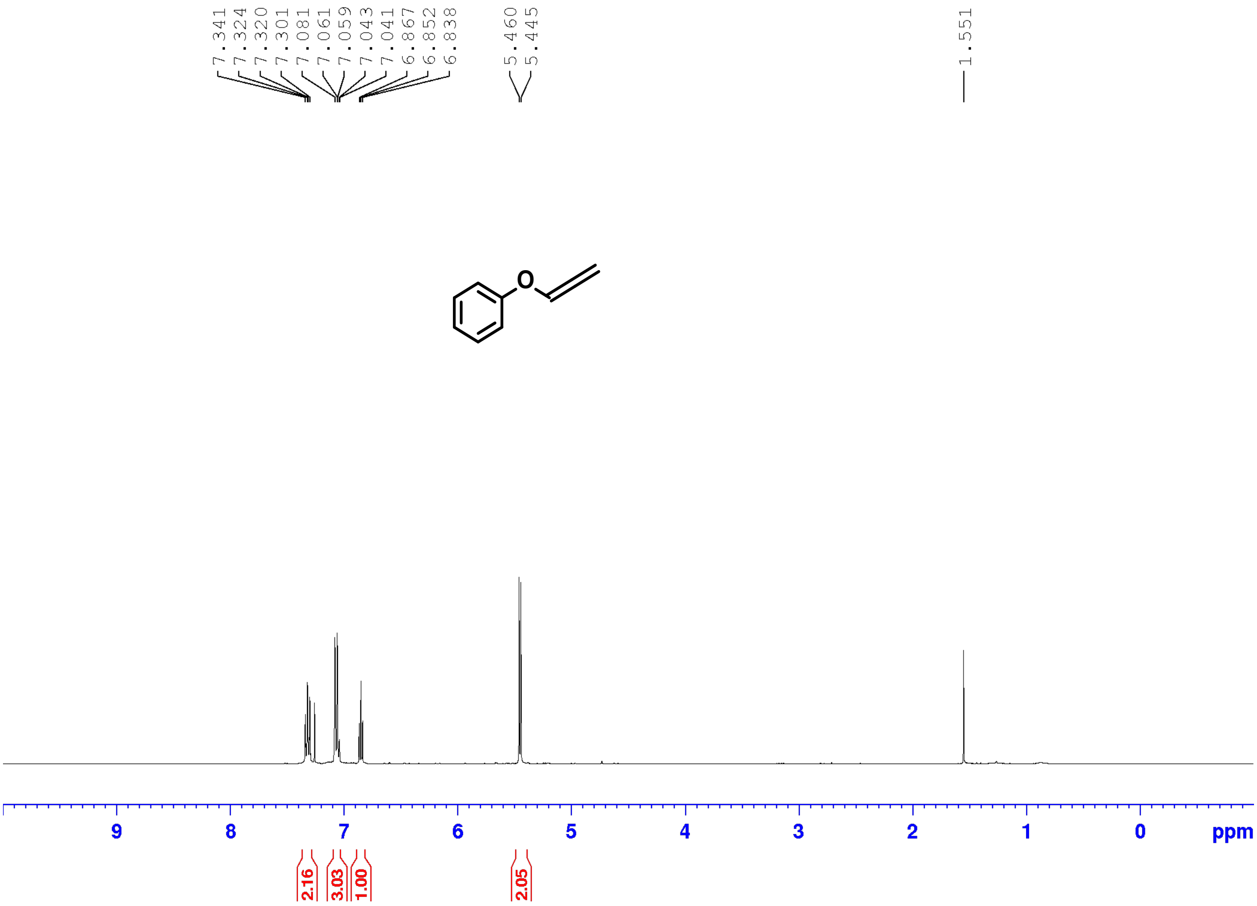
7.341
7.324
7.320
7.301
7.081
7.061
7.059
7.043
7.041
6.867
6.852
6.838
5.460
5.445
1.551
9
8
7
6
5
4
3
2
1
0
ppm
2.16
3.03
1.00
2.05
O

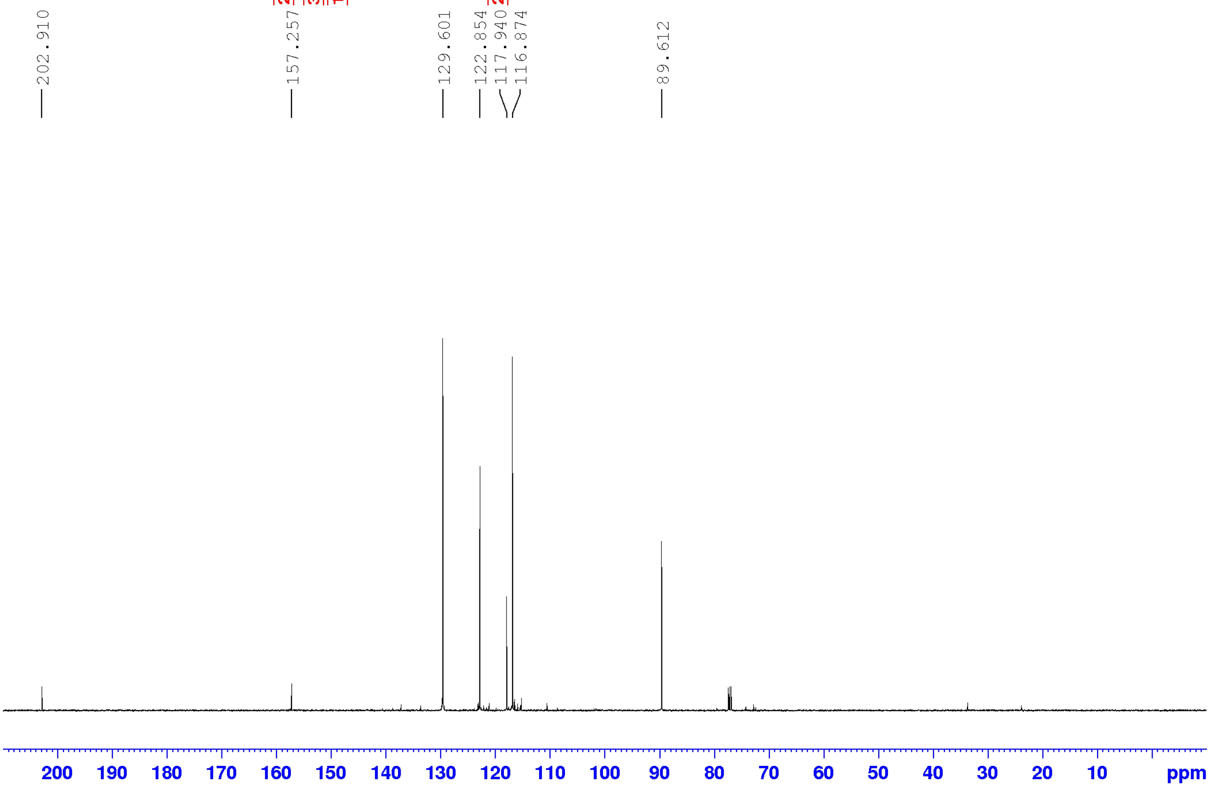
202.910
157.257
129.601
122.854
117.940
116.874
89.612
200
190
180
170
160
150
140
130
120
110
100
90
80
70
60
50
40
30
20
10
ppm

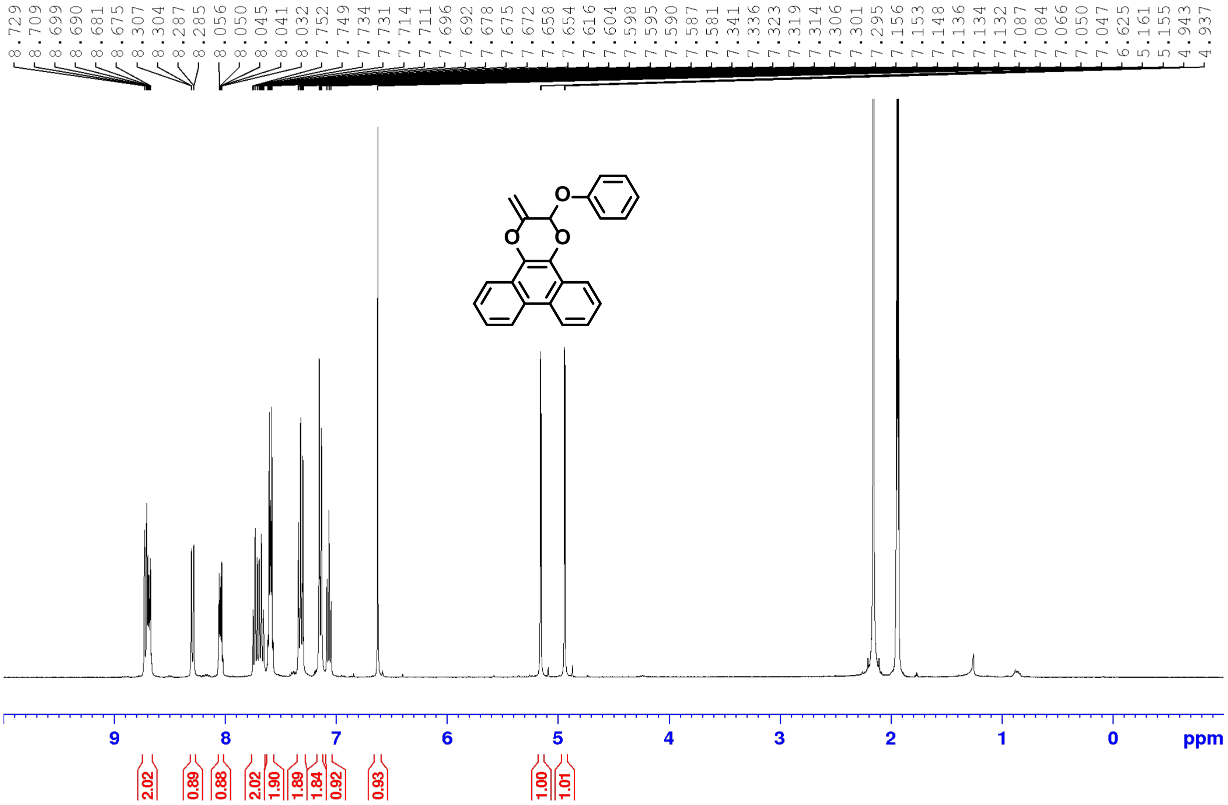
8.729 8.709 8.699 8.690 8.681 8.675 8.307 8.304 8.287 8.285 8.056 8.050 8.045 8.041 8.032 7.752 7.749 7.734 7.731 7.714 7.711 7.696 7.692 7.678 7.675 7.672 7.658 7.654 7.616 7.604 7.598 7.595 7.590 7.587 7.581 7.341 7.336 7.323 7.319 7.314 7.306 7.301 7.295 7.156 7.153 7.148 7.136 7.134 7.132 7.087 7.084 7.066 7.050 7.047 6.625 5.161 5.155 4.943 4.937
O
O
O
9
8
7
6
5
4
3
2
1
0
ppm
2.02
0.89
0.88
2.02
1.90
1.89
1.84
0.92
0.93
1.00
1.01

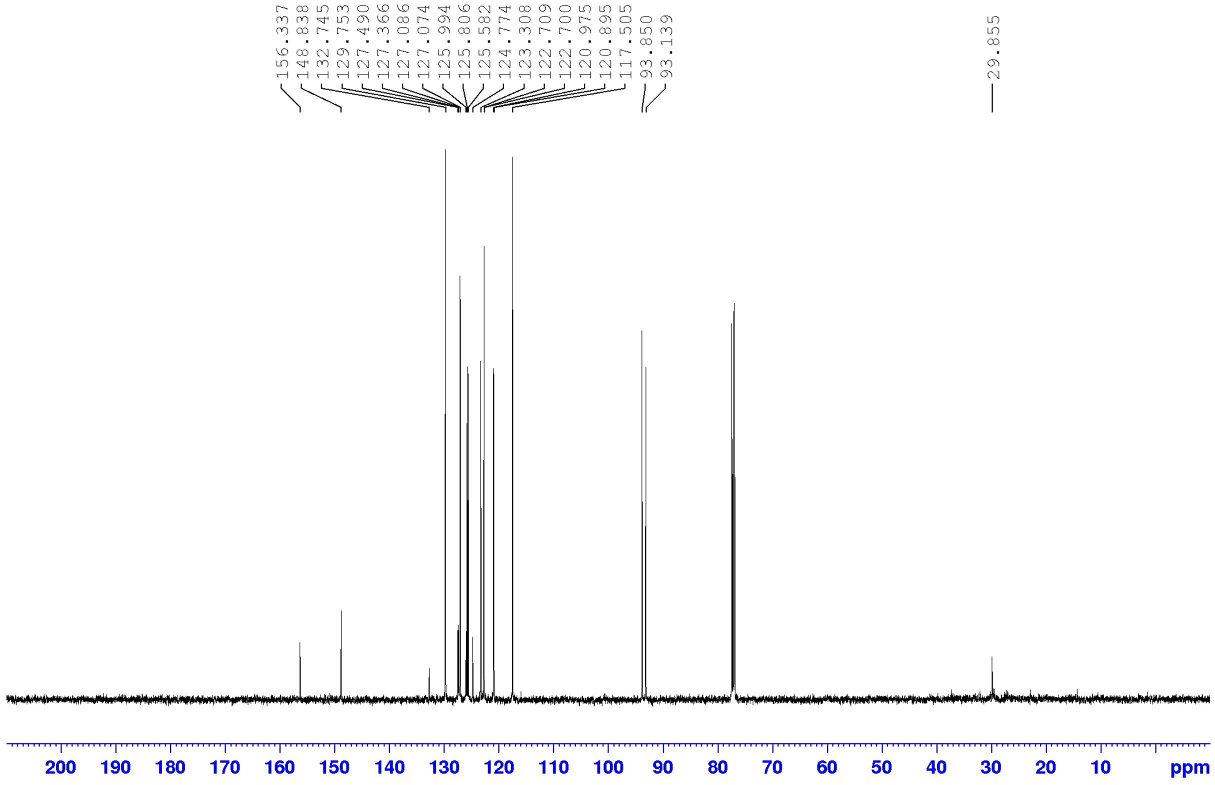
156.337 148.838 132.745 129.753 127.490 127.366 127.086 127.074 125.994 125.806 125.582 124.774 123.308 122.709 122.700 120.975 120.895 117.505 93.850 93.139
29.855
200
190
180
170
160
150
140
130
120
110
100
90
80
70
60
50
40
30
20
10
ppm

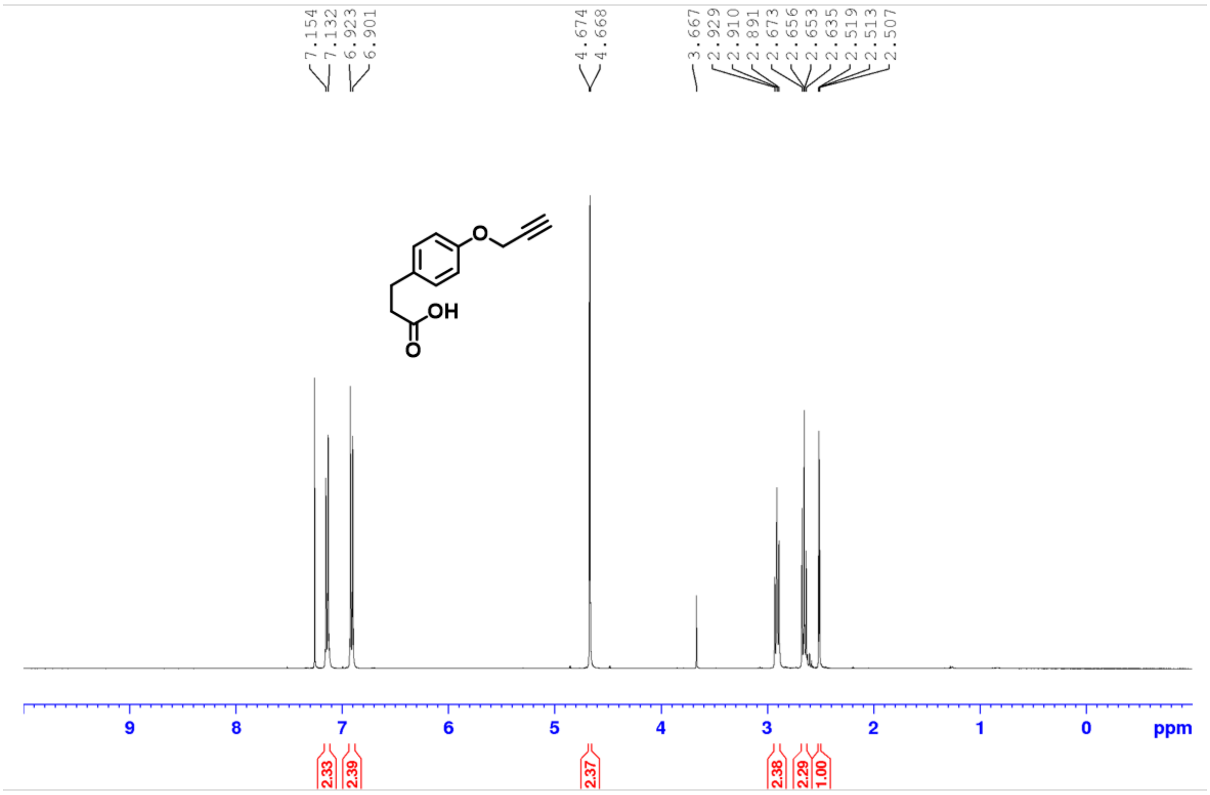
7.154
7.132
6.923
6.901
4.674
4.668
3.667
2.929
2.910
2.891
2.673
2.656
2.653
2.635
2.519
2.513
2.507
OH
O
O
9 8 7 6 5 4 3 2 1 0 ppm
2.33
2.39
2.37
2.38
2.29
1.00

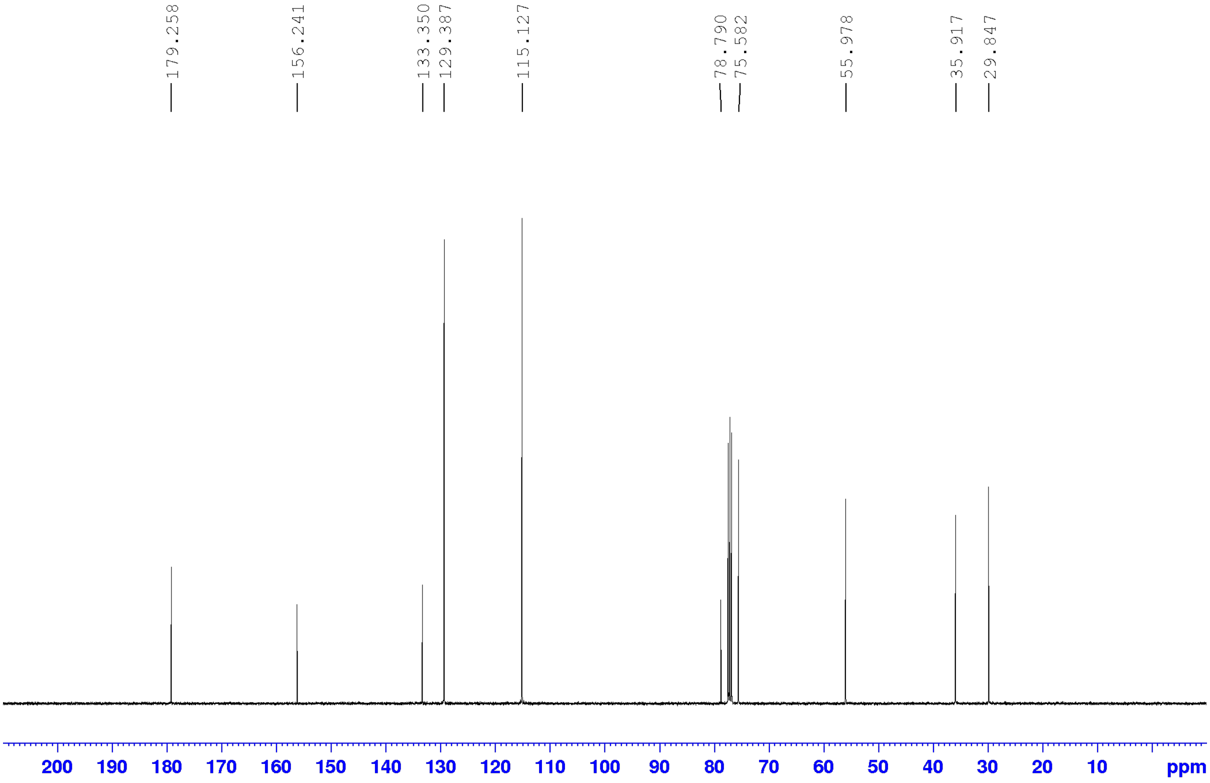
179.258
156.241
133.350
129.387
115.127
78.790
75.582
55.978
35.917
29.847
200 190 180 170 160 150 140 130 120 110 100 90 80 70 60 50 40 30 20 10 ppm

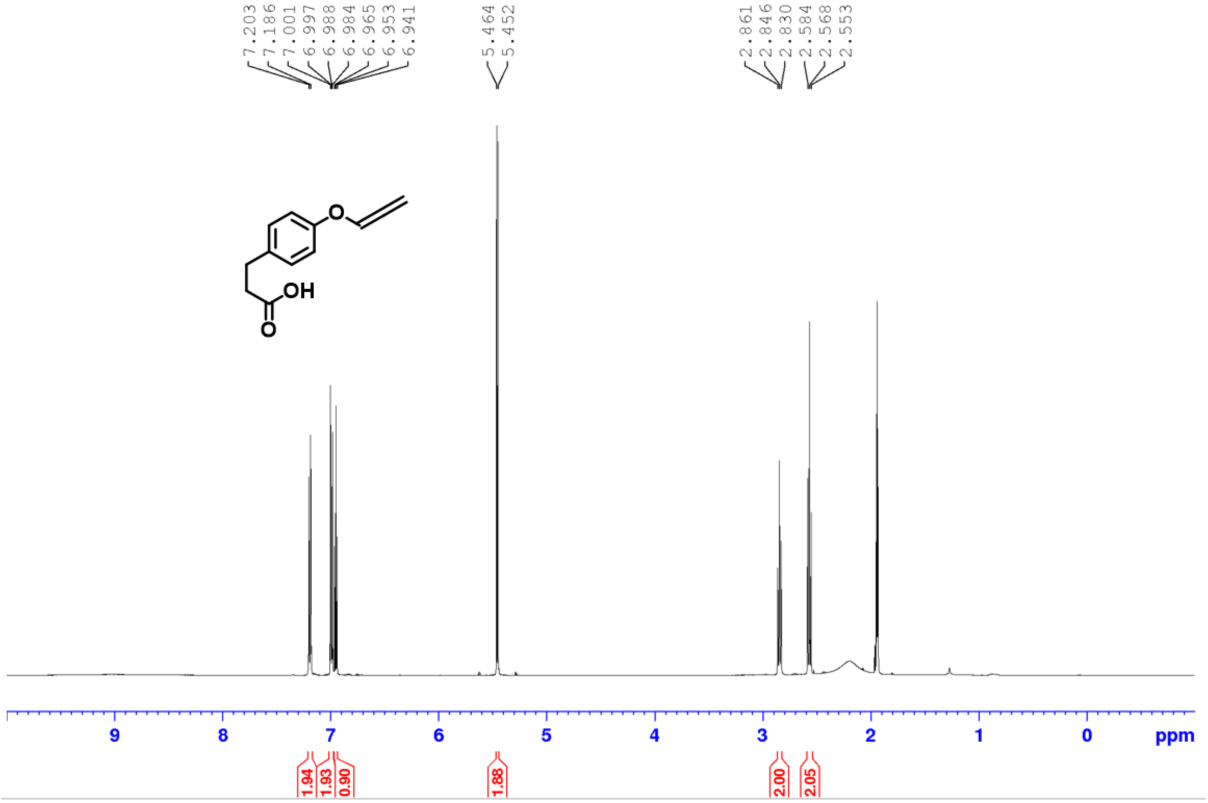
7.203
7.186
7.001
6.997
6.988
6.984
6.965
6.953
6.941
5.464
5.452
2.861
2.846
2.830
2.584
2.568
2.553
OH
O
O
9
8
7
6
5
4
3
2
1
0
ppm
1.94
1.93
0.90
1.88
2.00
2.05

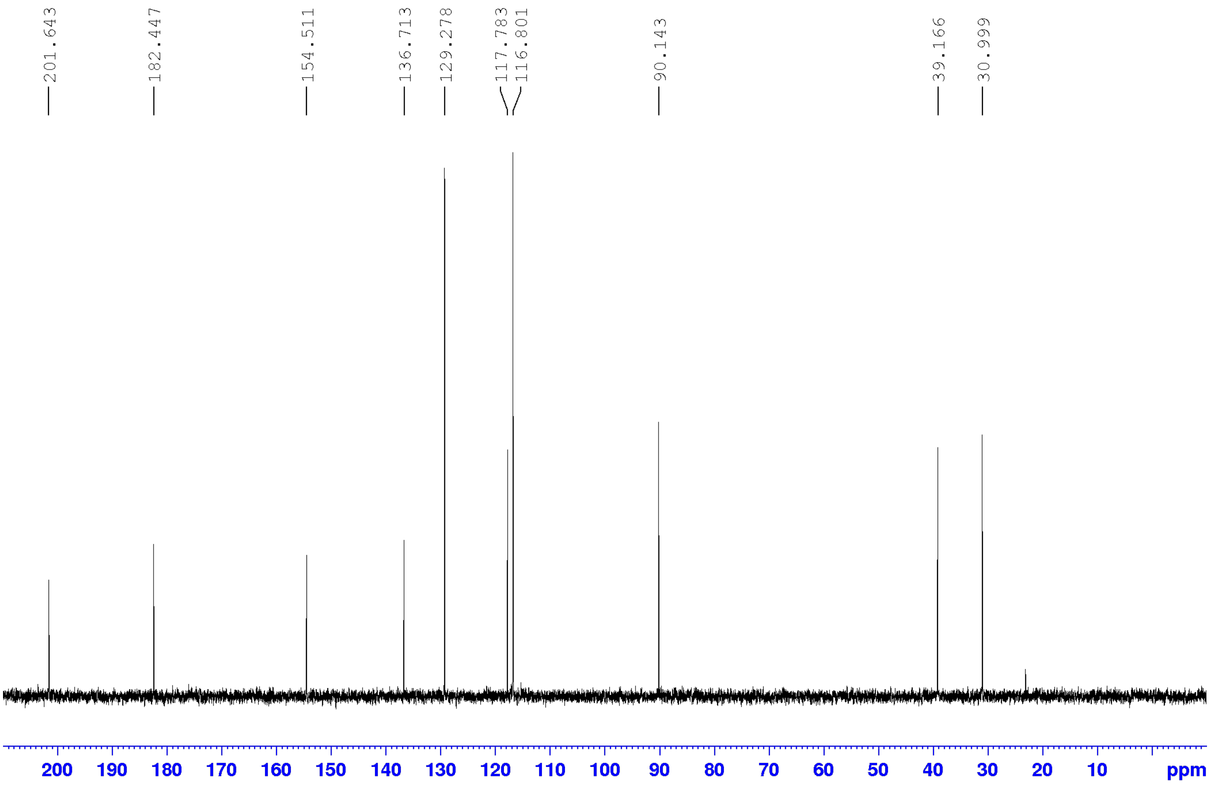
201.643
182.447
154.511
136.713
129.278
117.783
116.801
90.143
39.166
30.999
200
190
180
170
160
150
140
130
120
110
100
90
80
70
60
50
40
30
20
10
ppm

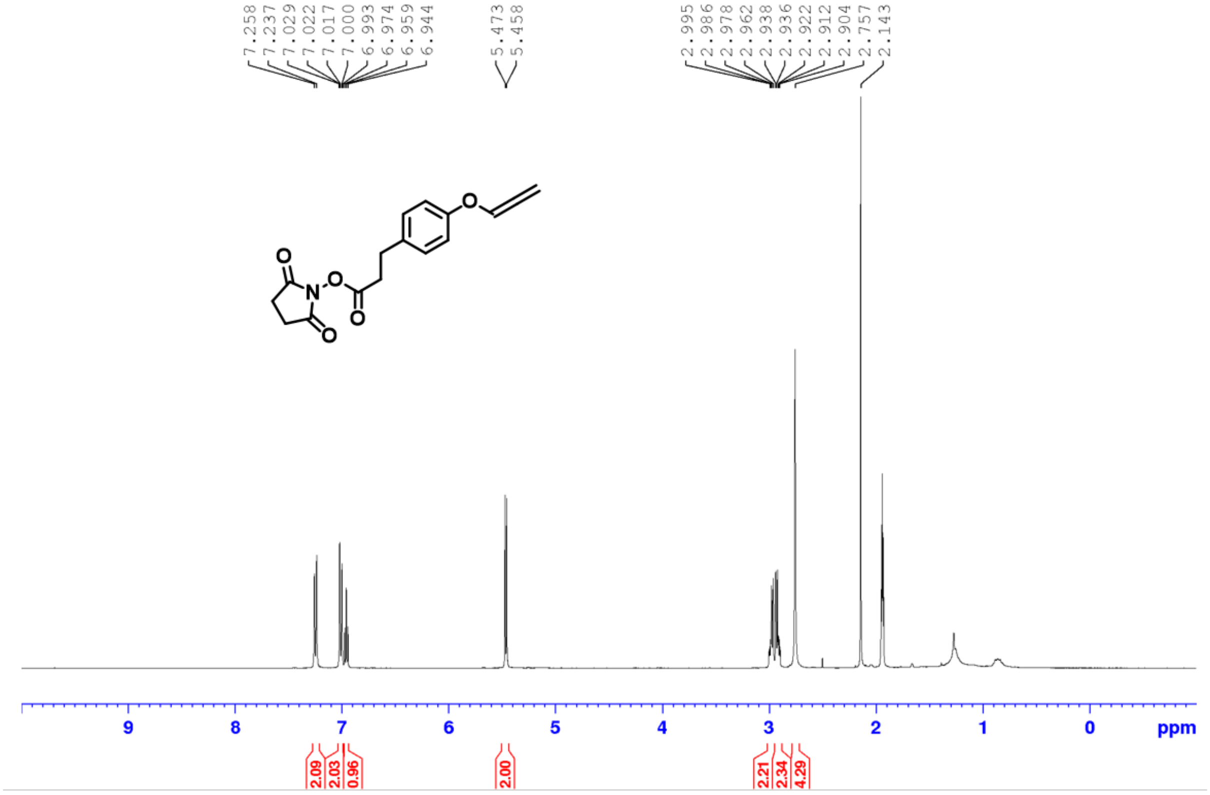
7.258
7.237
7.029
7.022
7.017
7.000
6.993
6.974
6.959
6.944
5.473
5.458
2.995
2.986
2.978
2.962
2.938
2.936
2.922
2.912
2.904
2.757
2.143
O
N
O
O
O
O
9
8
7
6
5
4
3
2
1
0
ppm
2.09
2.03
0.96
2.00
2.21
2.34
4.29

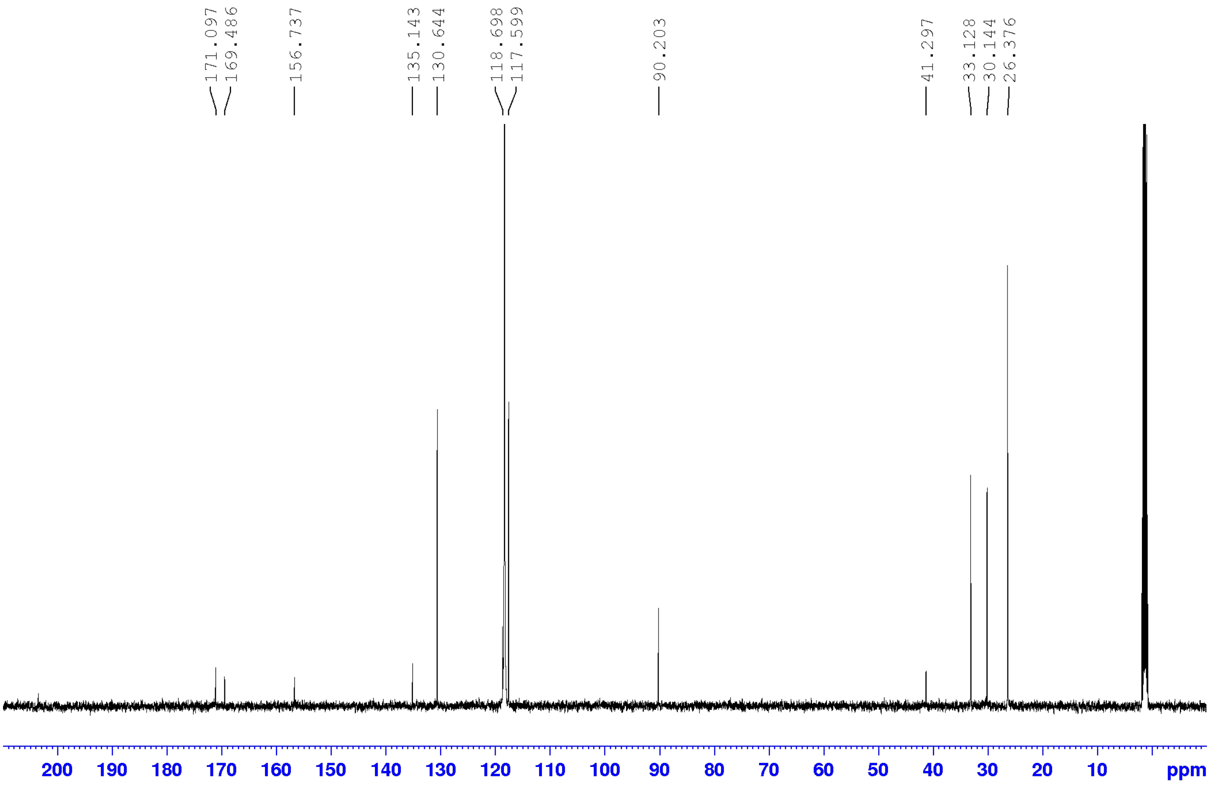
171.097
169.486
156.737
135.143
130.644
118.698
117.599
90.203
41.297
33.128
30.144
26.376
200
190
180
170
160
150
140
130
120
110
100
90
80
70
60
50
40
30
20
10
ppm

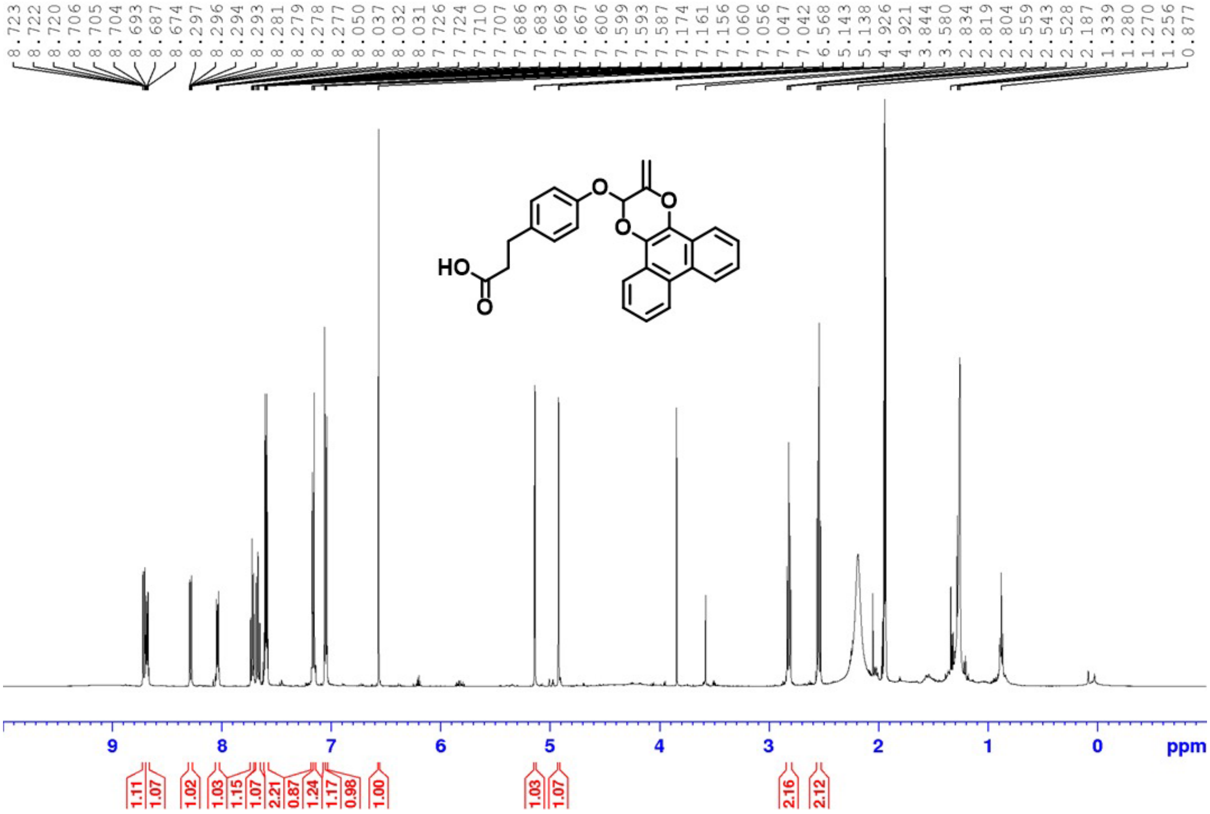
8.723 8.722 8.720 8.706 8.705 8.704 8.693 8.687 8.674 8.297 8.296 8.294 8.293 8.281 8.279 8.278 8.277 8.050 8.037 8.032 8.031 7.726 7.724 7.710 7.707 7.686 7.683 7.669 7.667 7.606 7.599 7.593 7.587 7.174 7.161 7.156 7.060 7.056 7.047 7.042 6.568 5.143 5.138 4.926 4.921 3.844 3.580 2.834 2.819 2.804 2.559 2.543 2.528 2.187 1.339 1.280 1.270 1.256 0.877
HO
O
9 8 7 6 5 4 3 2 1 0 ppm
1.11 1.07 1.02 1.03 1.15 1.07 2.21 0.87 1.24 1.17 0.98 1.00 1.03 1.07 2.16 2.12

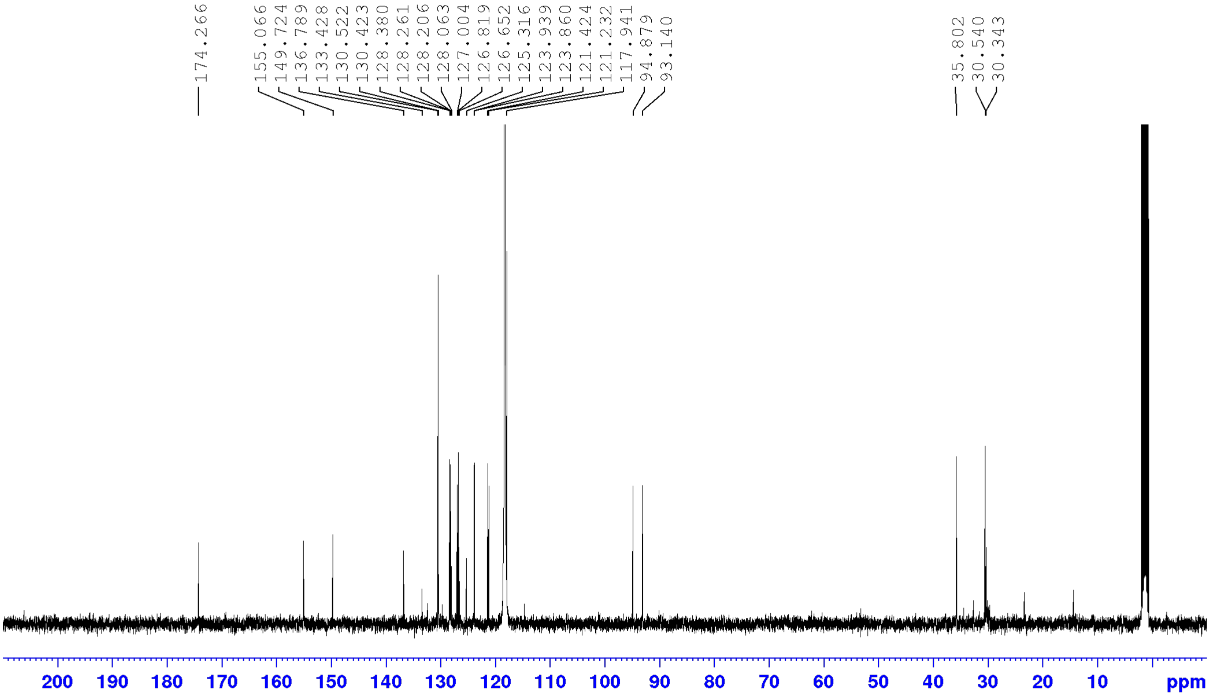
174.266
155.066 149.724 136.789 133.428 130.522 130.423 128.380 128.261 128.206 128.063 127.004 126.819 126.652 125.316 123.939 123.860 121.424 121.232 117.941 94.879 93.140
35.802 30.540 30.343
200 190 180 170 160 150 140 130 120 110 100 90 80 70 60 50 40 30 20 10 ppm

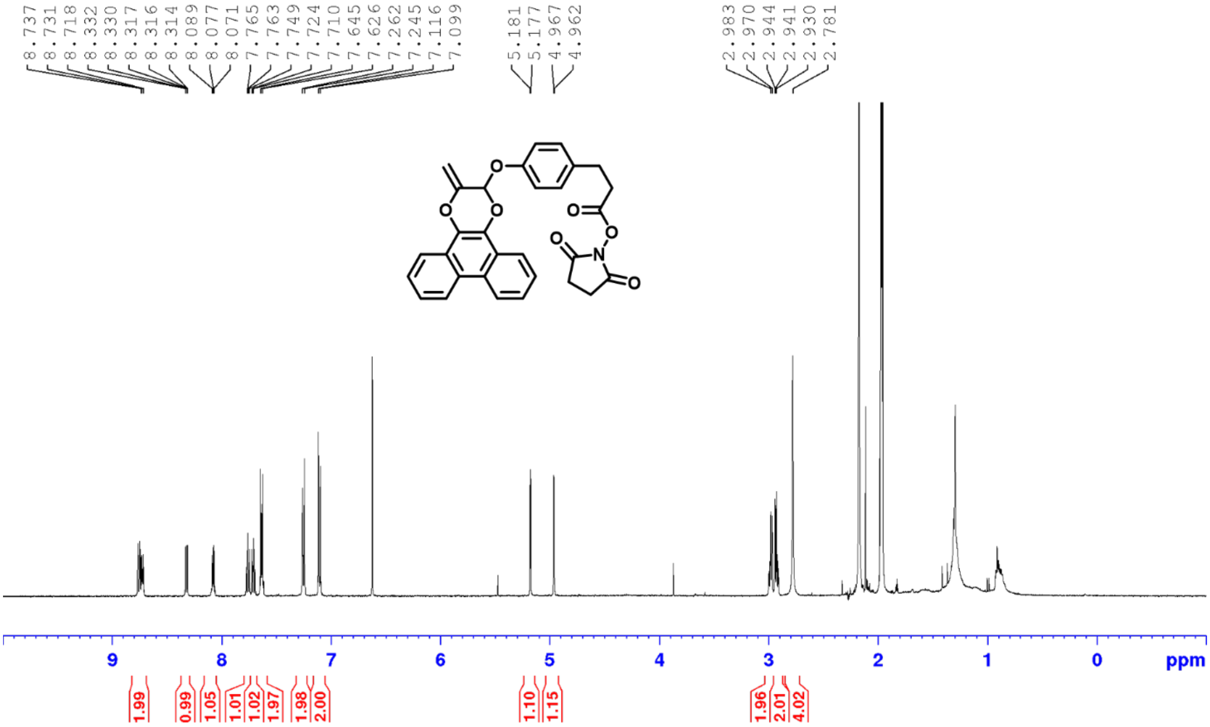
8.737
8.731
8.718
8.332
8.330
8.317
8.316
8.314
8.089
8.077
8.071
7.765
7.763
7.749
7.724
7.710
7.645
7.626
7.262
7.245
7.116
7.099
5.181
5.177
4.967
4.962
2.983
2.970
2.944
2.941
2.930
2.781
O
O
O
O
O
O
N
O
O
9
8
7
6
5
4
3
2
1
0
ppm
1.99
0.99
1.05
1.01
1.02
1.97
1.98
2.00
1.10
1.15
1.96
2.01
4.02

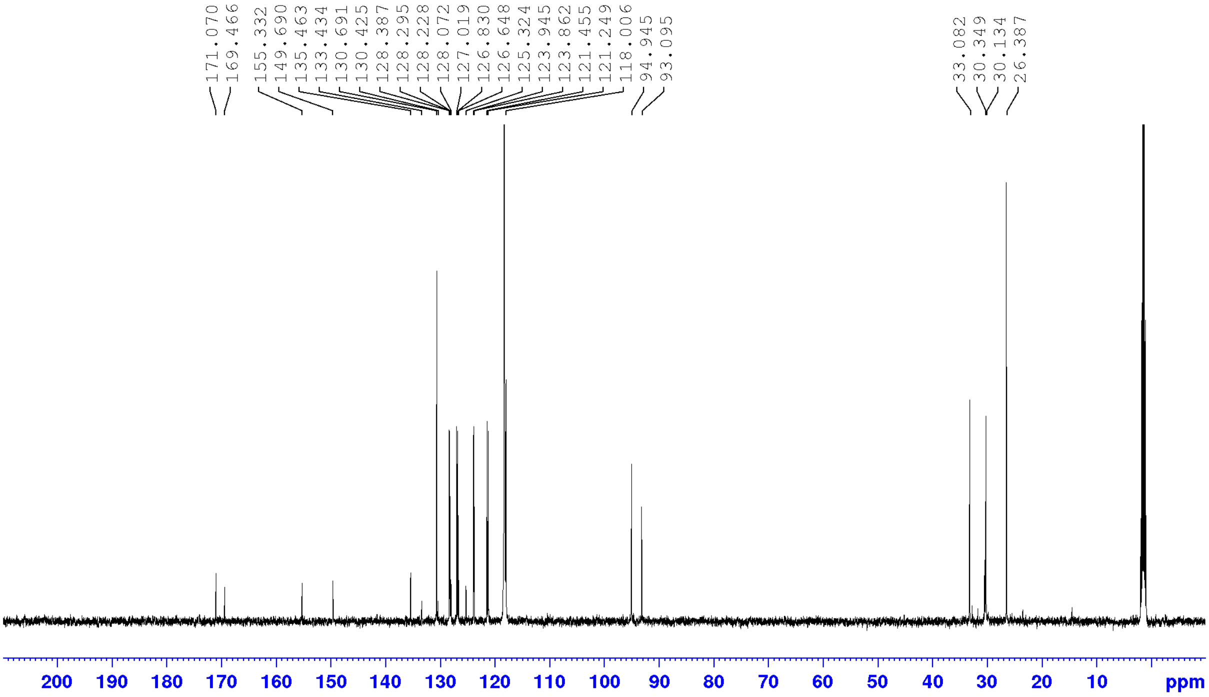
171.070
169.466
155.332
149.690
135.463
133.434
130.691
130.425
128.387
128.295
128.228
128.072
127.019
126.830
126.648
125.324
123.945
123.862
121.455
121.249
118.006
94.945
93.095
33.082
30.349
30.134
26.387
200
190
180
170
160
150
140
130
120
110
100
90
80
70
60
50
40
30
20
10
ppm

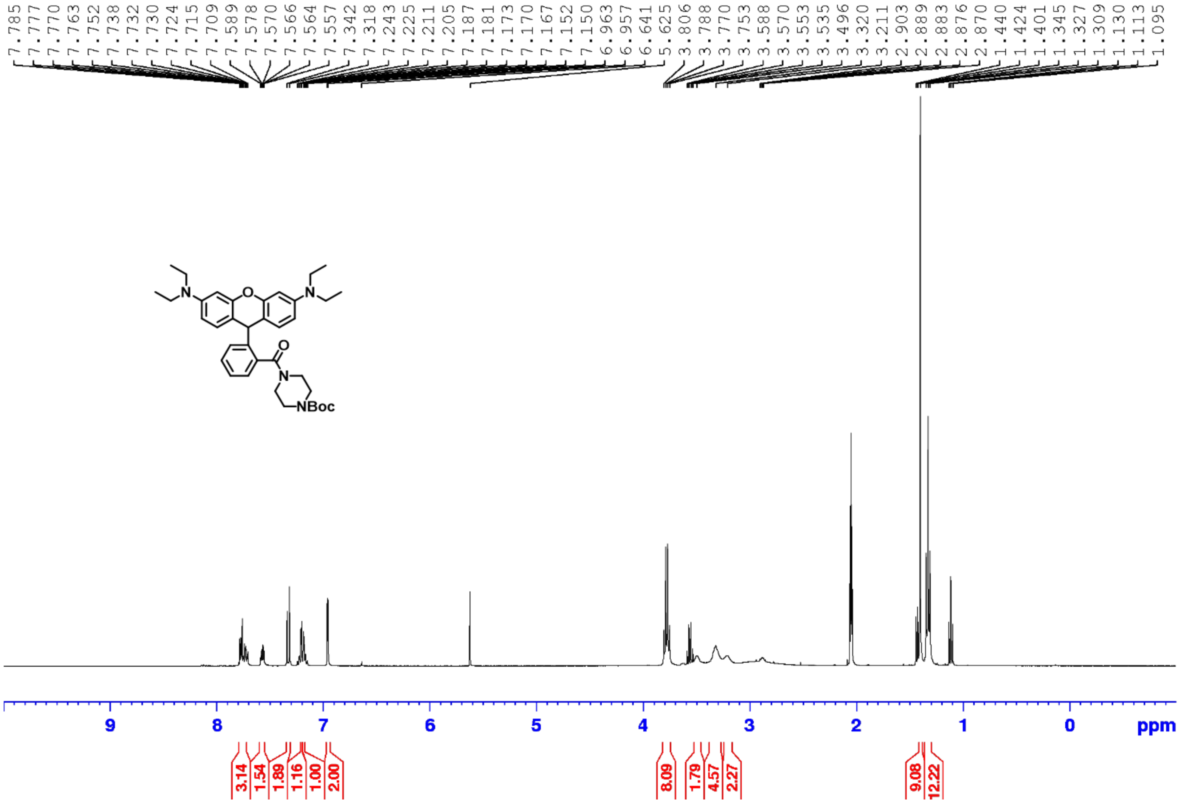
7.785 7.777 7.770 7.763 7.752 7.738 7.732 7.730 7.724 7.715 7.709 7.589 7.578 7.570 7.566 7.564 7.557 7.342 7.318 7.243 7.225 7.211 7.205 7.187 7.181 7.173 7.170 7.167 7.152 7.150 6.963 6.957 6.641 5.625 3.806 3.788 3.770 3.753 3.588 3.570 3.553 3.535 3.496 3.320 3.211 2.903 2.889 2.883 2.876 2.870 1.440 1.424 1.401 1.345 1.327 1.309 1.130 1.113 1.095
N
O
N
O
N
NBoc
9
8
7
6
5
4
3
2
1
0
ppm
3.14
1.54
1.89
1.16
1.00
2.00
8.09
1.79
4.57
2.27
9.08
12.22

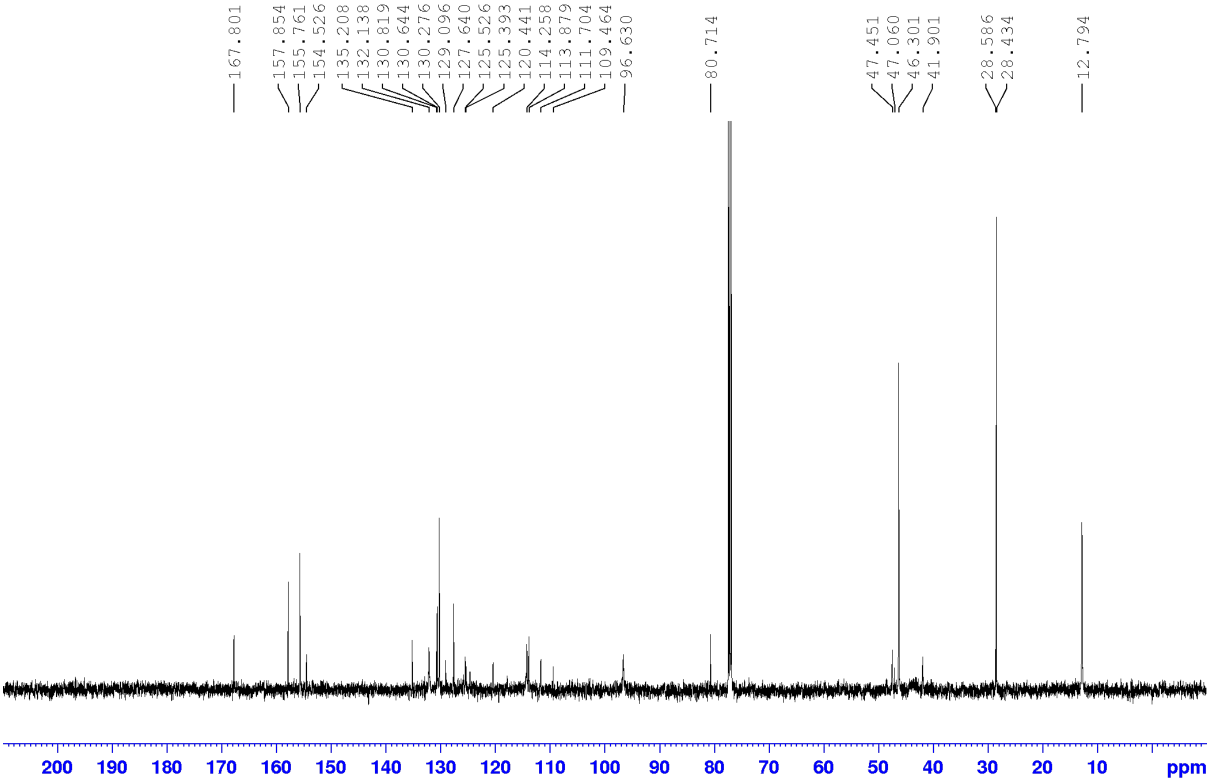
167.801
157.854 155.761 154.526
135.208 132.138 130.819 130.644 130.276 129.096 127.640 125.526 125.393 120.441 114.258 113.879 111.704 109.464 96.630
80.714
47.451 47.060 46.301 41.901
28.586 28.434
12.794
200
190
180
170
160
150
140
130
120
110
100
90
80
70
60
50
40
30
20
10
ppm

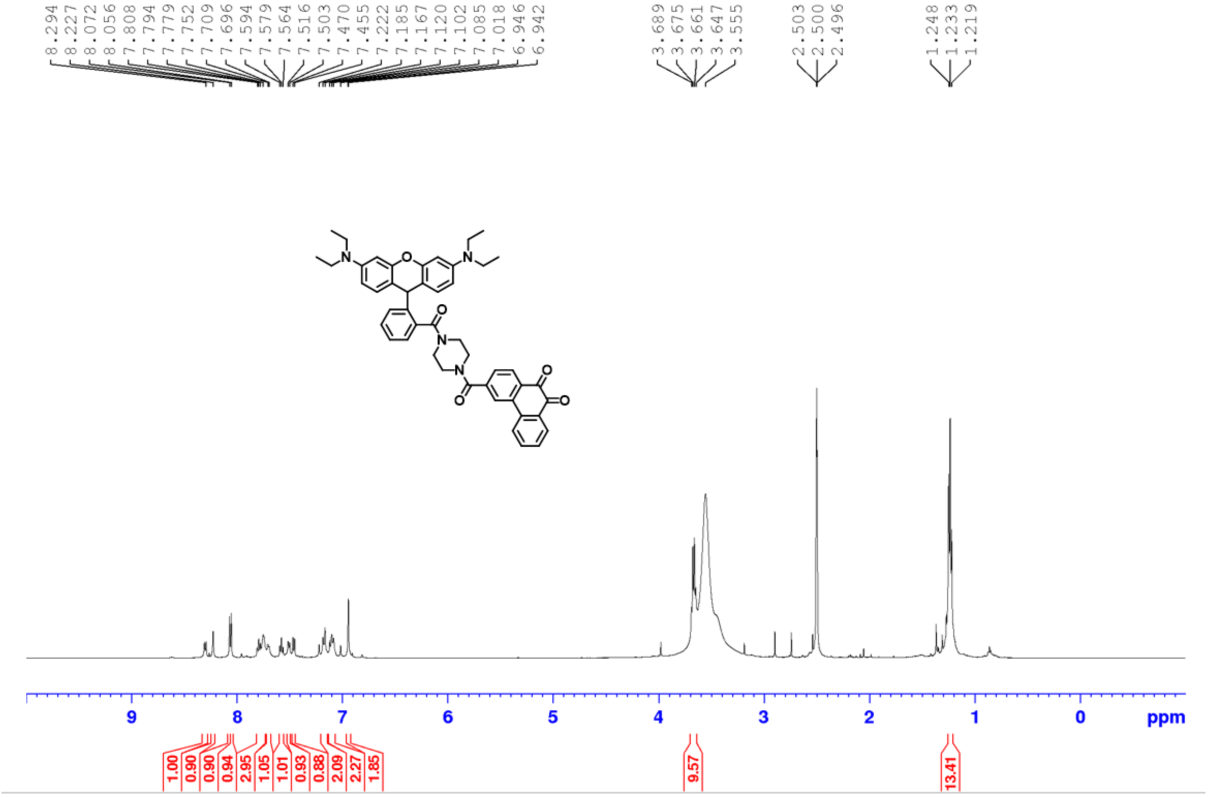
8.294
8.227
8.072
8.056
7.808
7.794
7.779
7.752
7.709
7.696
7.594
7.579
7.564
7.516
7.503
7.470
7.455
7.222
7.185
7.167
7.120
7.102
7.085
7.018
6.946
6.942
3.689
3.675
3.661
3.647
3.555
2.503
2.500
2.496
1.248
1.233
1.219
N
O
N
O
N
N
O
O
O
9 8 7 6 5 4 3 2 1 0 ppm
1.00
0.90
0.90
0.94
2.95
1.05
1.01
0.93
0.88
2.09
2.27
1.85
9.57
13.41

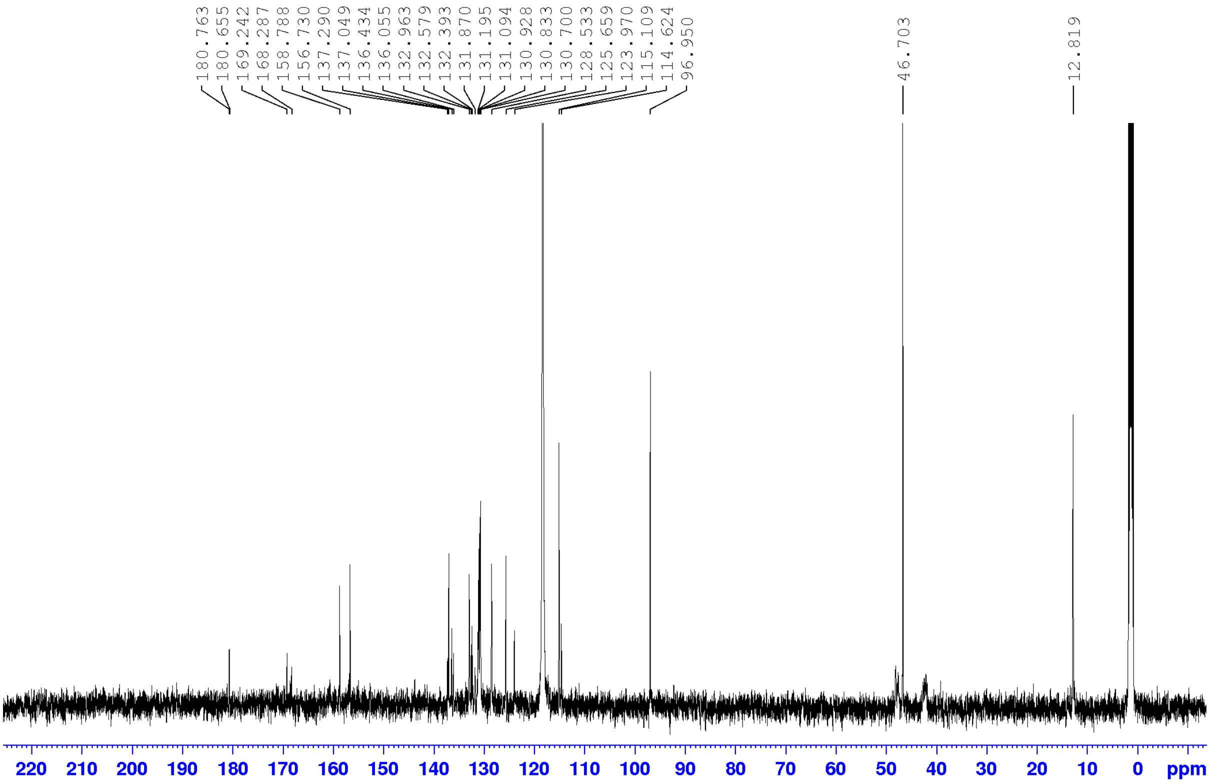
180.763
180.655
169.242
168.287
158.788
156.730
137.290
137.049
136.434
136.055
132.963
132.579
132.393
131.870
131.195
131.094
130.928
130.833
130.700
128.533
125.659
123.970
115.109
114.624
96.950
46.703
12.819
220 210 200 190 180 170 160 150 140 130 120 110 100 90 80 70 60 50 40 30 20 10 0 ppm

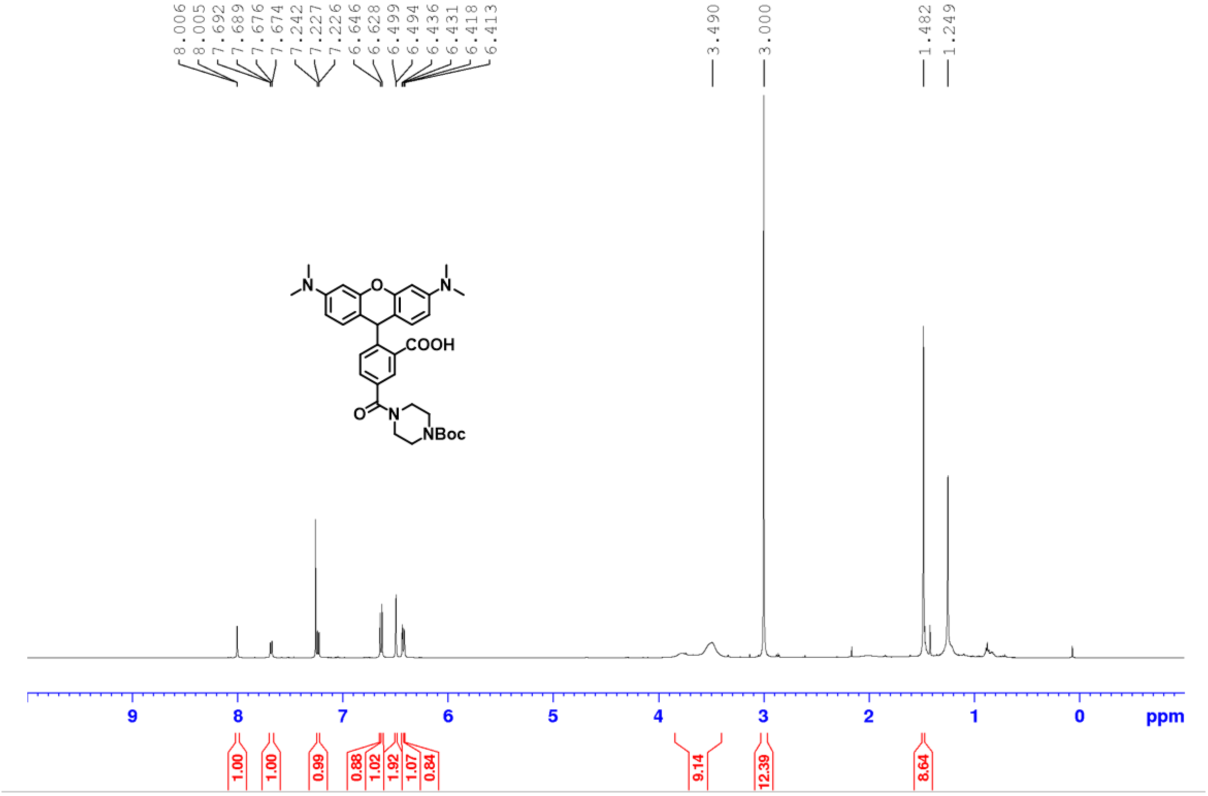
8.006
8.005
7.692
7.689
7.676
7.674
7.242
7.227
7.226
6.646
6.628
6.499
6.494
6.436
6.431
6.418
6.413
3.490
3.000
1.482
1.249
COOH
O
N
NBoc
9
8
7
6
5
4
3
2
1
0
ppm
1.00
1.00
0.99
0.88
1.02
1.92
1.07
0.84
9.14
12.39
8.64

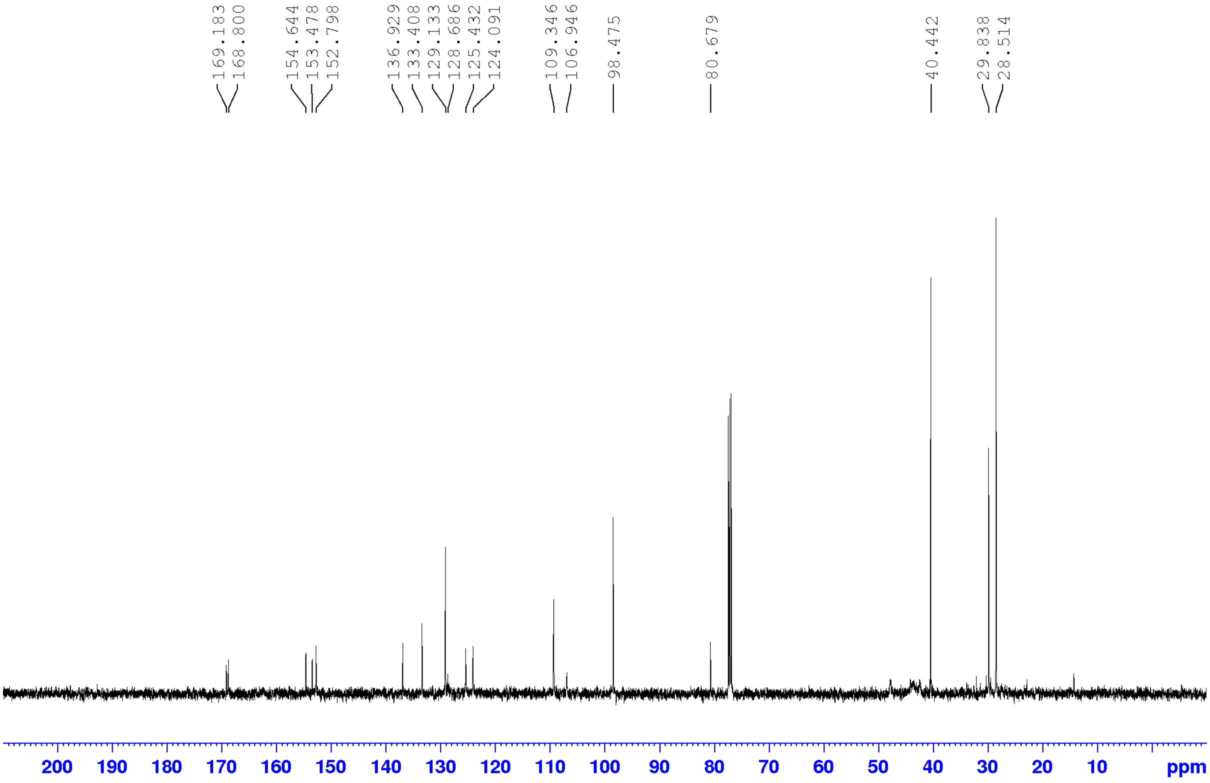
169.183
168.800
154.644
153.478
152.798
136.929
133.408
129.133
128.686
125.432
124.091
109.346
106.946
98.475
80.679
40.442
29.838
28.514
200
190
180
170
160
150
140
130
120
110
100
90
80
70
60
50
40
30
20
10
ppm

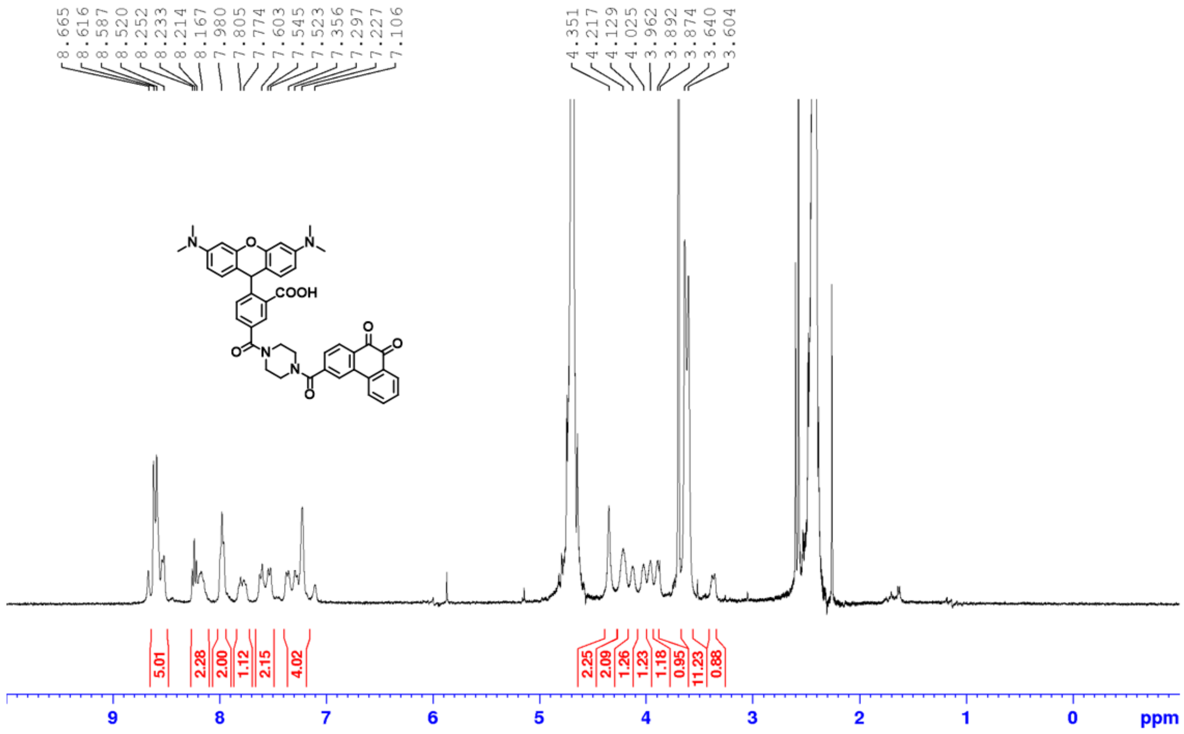

8.665
8.616
8.587
8.520
8.252
8.233
8.214
8.167
7.980
7.805
7.774
7.603
7.545
7.523
7.356
7.297
7.227
7.106
4.351
4.217
4.129
4.025
3.962
3.892
3.874
3.640
3.604
COOH
5.01
2.28
2.00
1.12
2.15
4.02
2.25
2.09
1.26
1.23
1.18
0.95
11.23
0.88
9
8
7
6
5
4
3
2
1
0
ppm

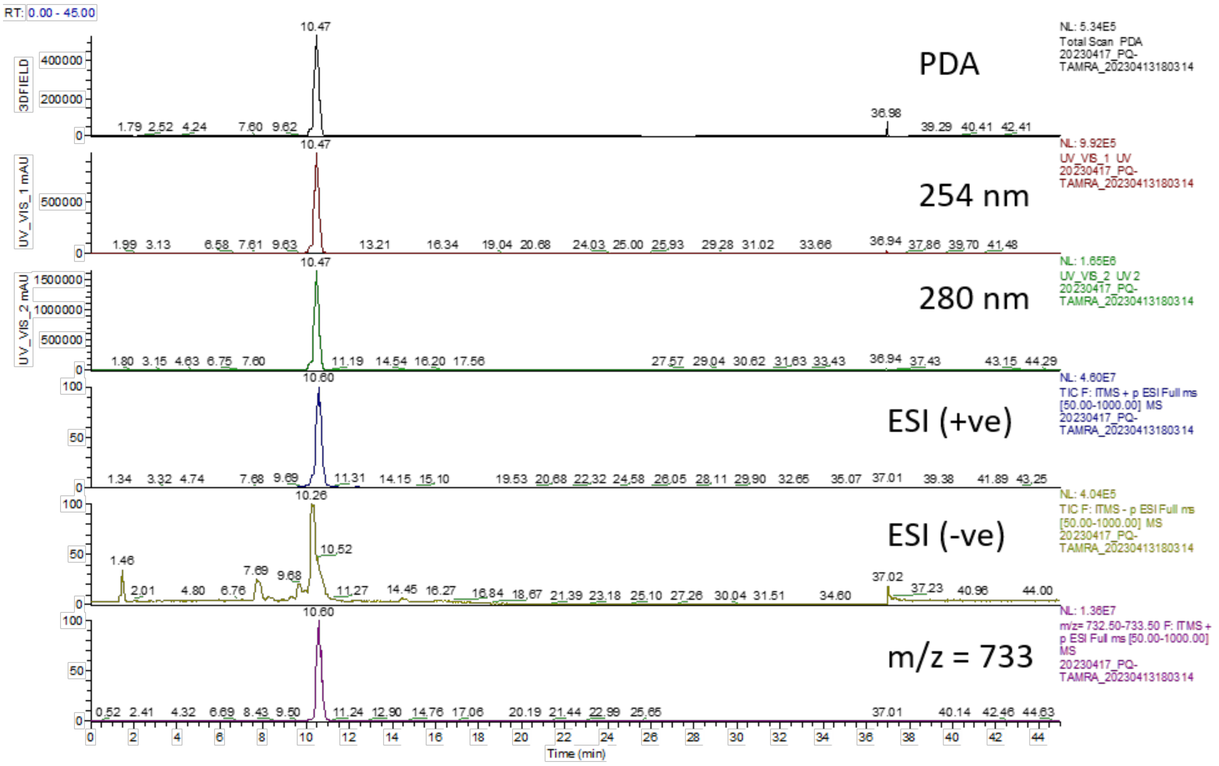

RT: 0.00 - 45.00
PDA
254 nm
280 nm
ESI (+ve)
ESI (-ve)
m/z = 733
NL: 5.34E5
Total Scan PDA
20230417_PQ-TAMRA_20230413180314
NL: 9.92E5
UV_VIS_1 UV
20230417_PQ-TAMRA_20230413180314
NL: 1.65E6
UV_VIS_2 UV 2
20230417_PQ-TAMRA_20230413180314
NL: 4.60E7
TIC F: ITMS + p ESI Full ms [50.00-1000.00] MS
20230417_PQ-TAMRA_20230413180314
NL: 4.04E5
TIC F: ITMS - p ESI Full ms [50.00-1000.00] MS
20230417_PQ-TAMRA_20230413180314
NL: 1.36E7
m/z= 732.50-733.50 F: ITMS + p ESI Full ms [50.00-1000.00] MS
20230417_PQ-TAMRA_20230413180314
3DFIELD
UV_VIS_1 mAU
UV_VIS_2 mAU
10.47
10.60
10.26
Time (min)

HPLC chromatograms of synthesized compounds

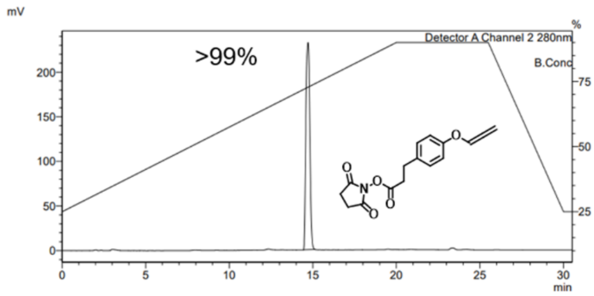

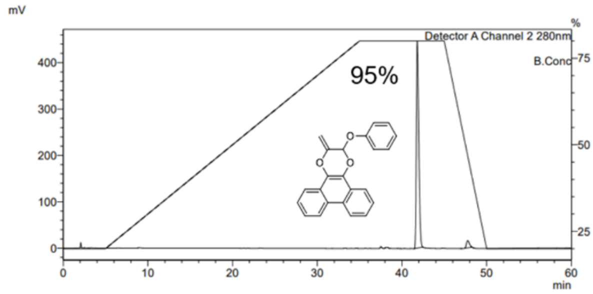

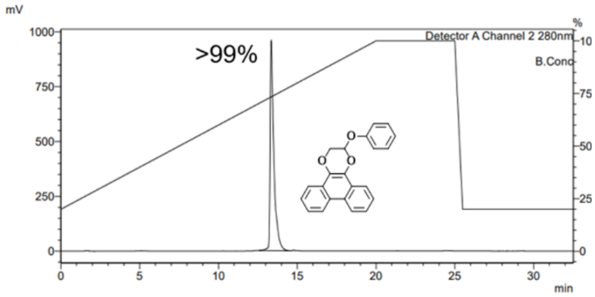

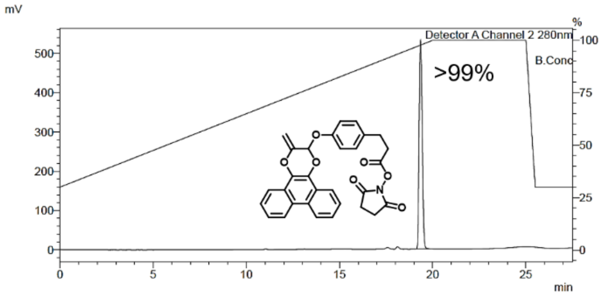

High-Resolution Mass Spectrometry Spectra

Mass Spectrum SmartFormula Report

**Sample Name**	PHQ8	**Data File**	D:\01_Chem\2025\202502\20250218\PHQ8.D
**Instrument Name**	Agilent 7200 GC-QTOF	**IRM Calibration Status**	Success
**Acq Method**	EIHR_CalValve.ei.M	**Acquired Time**	18/2/2025 12:32:14 PM (UTC+08:00)
**Comment**	Prof Ang Wee Han	**Operator**	SY

Meas. m/z	#	Formula	Calc. Mass	Err [ppm]
132.0565	1	C9 H8 O	132.057	3.79

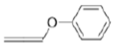

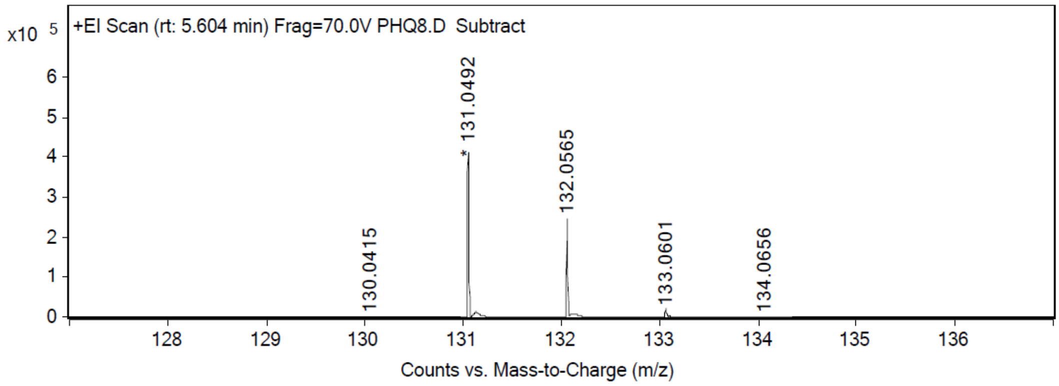

Mass Spectrum SmartFormula Report

**Sample Name**	PHQ-3	**Data File**	D:\01_Chem\2025\202502\20250212\PHQ-3.D
**Instrument Name**	Agilent 7200 GC-QTOF	**IRM Calibration Status**	Success
**Acq Method**	EIHR_CalValve.ei.M	**Acquired Time**	12/2/2025 1:11:35 PM (UTC+08:00)
**Comment**	Prof Ang Wee Han	**Operator**	SY

Meas. m/z	#	Formula	Calc. Mass	Err [ppm]
328.1092	1	C22 H16 O3	328.1094	0.61

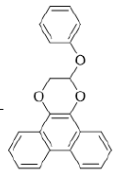

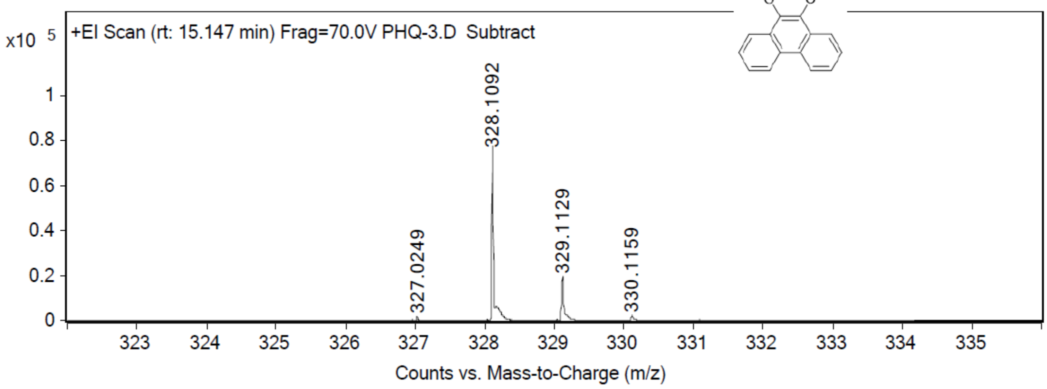

Mass Spectrum SmartFormula Report

**Sample Name**	PHQ-4	**Data File**	D:\01_Chem\2025\202502\20250212\PHQ-4.D
**Instrument Name**	Agilent 7200 GC-QTOF	**IRM Calibration Status**	Success
**Acq Method**	EIHR_CalValve.ei.M	**Acquired Time**	12/2/2025 2:01:58 PM (UTC+08:00)
**Comment**	Prof Ang Wee Han	**Operator**	SY

Meas. m/z	#	Formula	Calc. Mass	Err [ppm]
340.1091	1	C23 H16 O3	340.1094	0.88

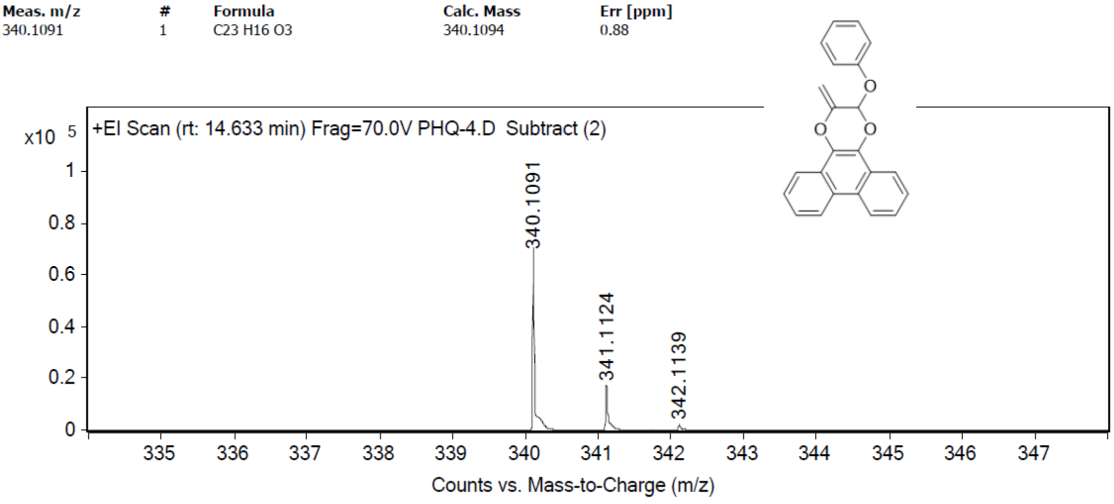

Page 1 of 1 Printed at 9:29 AM on 13-Feb-2025

Mass Spectrum SmartFormula Report

**Sample Name**	PHQ6	**Data File**	D:\01_Chem\2025\202502\20250218\PHQ6.D
**Instrument Name**	Agilent 7200 GC-QTOF	**IRM Calibration Status**	Success
**Acq Method**	EIHR_CalValve.ei.M	**Acquired Time**	18/2/2025 11:38:30 AM (UTC+08:00)
**Comment**	Prof Ang Wee Han	**Operator**	SY

Meas. m/z	#	Formula	Calc. Mass	Err [ppm]
204.0779	1	C12 H12 O3	204.0781	0.98

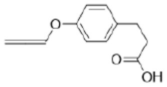

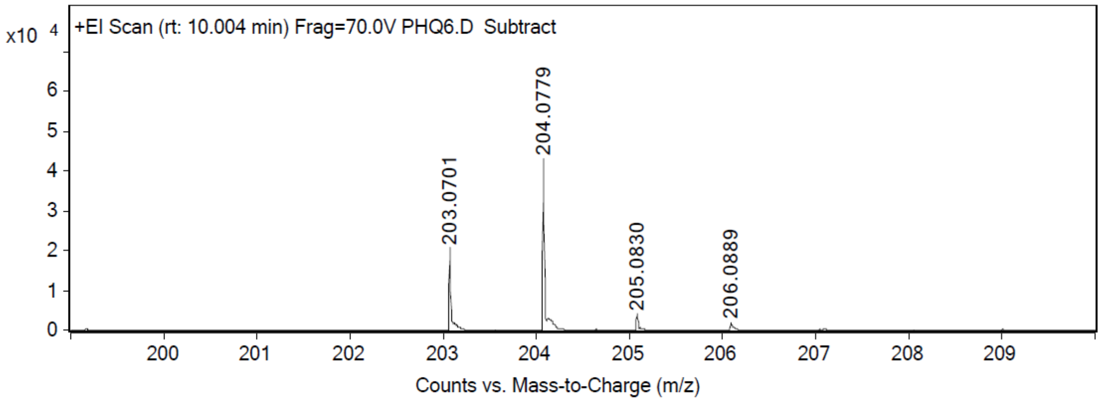

Page 1 of 1 Printed at 2:52 PM on 18-Feb-2025

Mass Spectrum SmartFormula Report

**Sample Name**	PHQ7	**Data File**	D:\MassHunter\Data\Chemistry\2025\202502\20250221-apci\PHQ7-1.d
**Instrument Name**	Agilent 6546 LC-QTOF	**IRM Calibration Status**	Some Ions Missed
**Acq Method**	MS Scan_union_APCI-3.m	**Acquired Time**	21/2/2025 11:38:31 AM (UTC+08:00)
**Comment**	Prof Ang Wee Han	**Operator**	WLK

**Meas. m/z**	**#**	**Formula**	**Calc. Mass**	**Err [ppm]**
412.1311	1	C26 H20 O5	412.1305	1.46

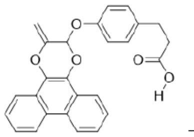

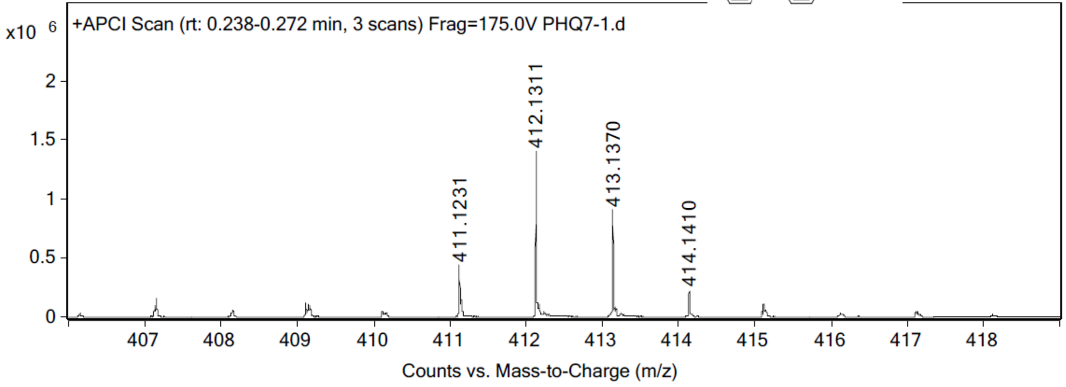

Mass Spectrum SmartFormula Report

**Sample Name**	PHQ-2	**Data File**	D:\01_Chem\2025\202502\20250212\PHQ-2-1.D
**Instrument Name**	Agilent 7200 GC-QTOF	**IRM Calibration Status**	Success
**Acq Method**	EIHR_CalValve.ei.M	**Acquired Time**	13/2/2025 11:58:42 AM (UTC+08:00)
**Comment**	Prof Ang Wee Han	**Operator**	SY

**Meas. m/z**	**#**	**Formula**	**Calc. Mass**	**Err [ppm]**
301.0944	1	C16 H15 N O5	301.0945	0.33

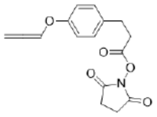

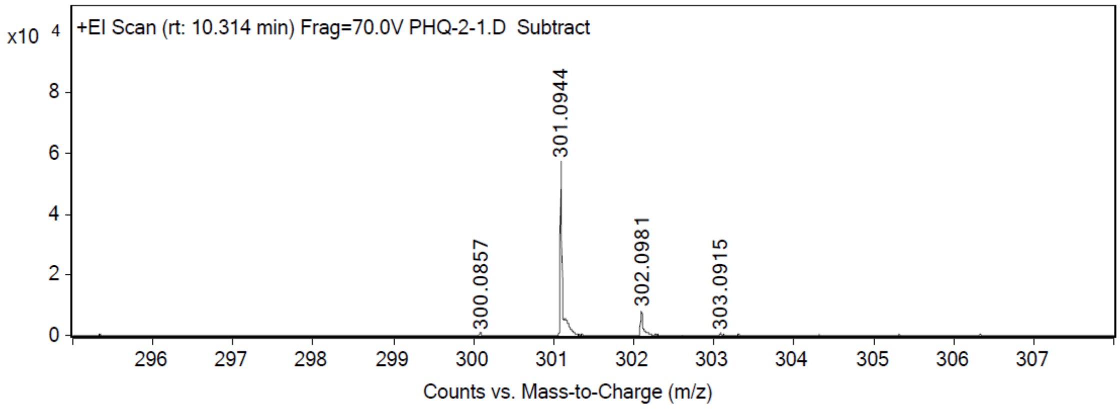

Mass Spectrum SmartFormula Report

**Sample Name**	PHQ1	**Data File**	D:\MassHunter\Data\Chemistry\2025\202502\20250214\PHQ1.d
**Instrument Name**	Agilent 6546 LC-QTOF	**IRM Calibration Status**	Some Ions Missed
**Acq Method**	MS Scan_union_APCI-3.m	**Acquired Time**	14/2/2025 11:01:10 AM (UTC+08:00)
**Comment**	Prof Ang Wee Han	**Operator**	WLK

Meas. m/z	#	Formula	Calc. Mass	Err [ppm]
510.1551	1	C30 H24 N O7	510.1547	0.78

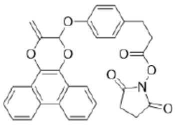

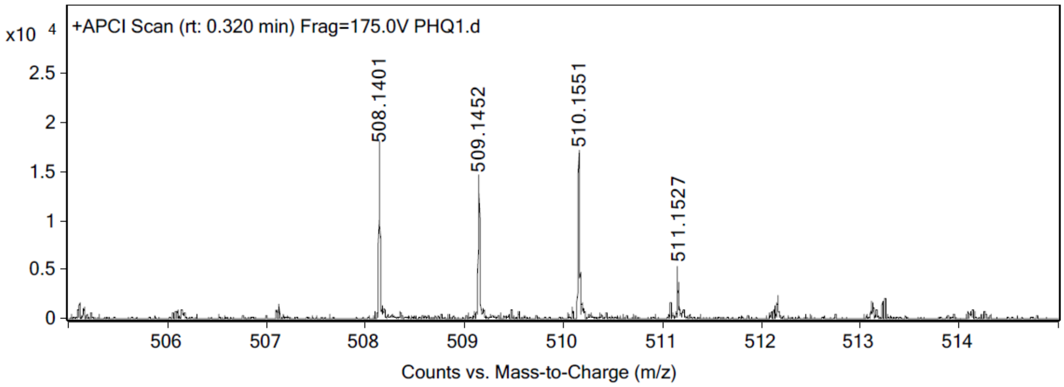

Mass Spectrum SmartFormula Report

**Sample Name**	Rho-PQ	**Data File**	D:\MassHunter\Data\Chemistry\2024\202401\20240123\Rho-PQ.d
**Instrument Name**	Agilent 6546 LC-QTOF	**IRM Calibration Status**	All Ions Missed
**Acq Method**	MS Scan_union-1.m	**Acquired Time**	23/1/2024 9:40:27 AM (UTC+08:00)
**Comment**	A/P ang Wee Han	**Operator**	WLK

Meas. m/z	#	Formula	Calc. Mass	Err [ppm]
745.3391	1	C47 H45 N4 O5	745.3384	0.94

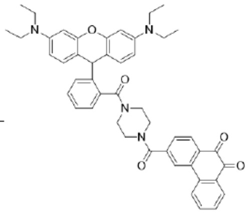

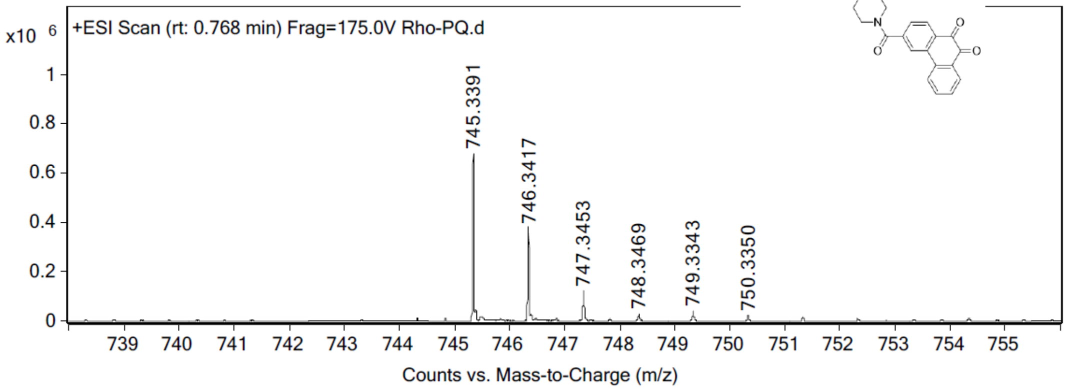

Mass Spectrum SmartFormula Report

**Sample Name**	TAMRA-PQ	**Data File**	D:\MassHunter\Data\Chemistry\2024\202401\20240123\TAMRA-PQ.d
**Instrument Name**	Agilent 6546 LC-QTOF	**IRM Calibration Status**	Success
**Acq Method**	MS Scan_union-1.m	**Acquired Time**	23/1/2024 9:49:09 AM (UTC+08:00)
**Comment**	A/P ang Wee Han	**Operator**	WLK

Meas. m/z	#	Formula	Calc. Mass	Err [ppm]
733.266	1	C44 H37 N4 O7	733.2657	0.41

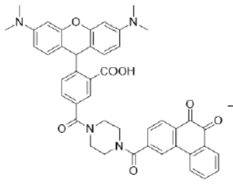

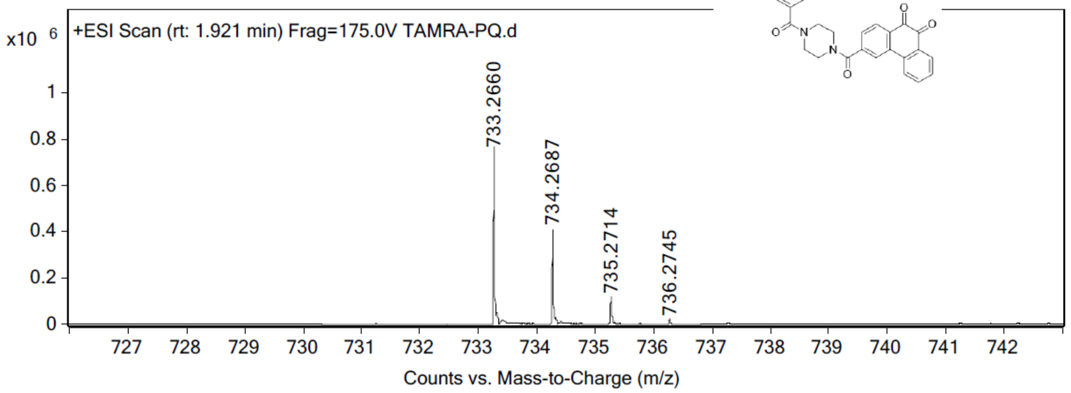

References

[1] Y. Li, J. Chen, R. Qiu, X. Wang, J. Long, L. Zhu, C.-T. Au, X. Xu, *Tetrahedron Lett.* **2015**, *56*, 5504-5507.

[2] G. Zong, Z. Hu, S. O'Keefe, D. Tranter, M. J. Iannotti, L. Baron, B. Hall, K. Corfield, A. O. Paatero, M. J. Henderson, P. Roboti, J. Zhou, X. Sun, M. Govindarajan, J. M. Rohde, N. Blanchard, R. Simmonds, J. Inglese, Y. Du, C. Demangel, S. High, V. O. Paavilainen, W. Q. Shi, *J. Am. Chem. Soc.* **2019**, *141*, 8450-8461.

[3] J. V. Staros, R. W. Wright, D. M. Swingle, *Anal. Biochem.* **1986**, *156*, 220-222.

[4] P. Dong, J. Stellmacher, L. M. Bouchet, M. Nieke, A. Kumar, E. R. Osorio-Blanco, G. Nagel, S. B. Lohan, C. Teutloff, A. Patzelt, M. Schäfer-Korting, M. Calderón, M. C. Meinke, U. Alexiev, *Angew. Chem. Int. Ed.* **2021**, *60*, 14938-14944.

[5] M. Baalmann, M. J. Ziegler, P. Werther, J. Wilhelm, R. Wombacher, *Bioconjugate Chem.* **2019**, *30*, 1405-1414.

[6] J. Li, H. Kong, L. Huang, B. Cheng, K. Qin, M. Zheng, Z. Yan, Y. Zhang, *J. Am. Chem. Soc.* **2018**, *140*, 14542-14546.
